# Chalcone and *Trans*-Chalcone Induce Transcriptomic Changes in *Caenorhabditis elegans* Compatible with a Novel Cumulative Damage Mode of Action

**DOI:** 10.3390/molecules31091411

**Published:** 2026-04-24

**Authors:** Giulio Galli, Carl S. Bruun, Carlos García-Estrada, Rafael Balaña-Fouce, María Martinez-Valladares, Tina V. A. Hansen

**Affiliations:** 1 Departamento de Ciencias Biomédicas, Facultad de Veterinaria, Universidad de León, Campus de Vegazana s/n, 24071 León, Spain; ggal@unileon.es (G.G.); cgare@unileon.es (C.G.-E.); 2 Department of Veterinary and Animal Sciences, University of Copenhagen, 1870 Frederiksberg C, Denmark; casb@sund.ku.dk; 3 Instituto de Biomedicina (IBIOMED), Universidad de León, Campus de Vegazana s/n, 24071 León, Spain; 4 Departamento Sanidad Animal, Instituto de Ganadería de Montaña, Conesjo Superior de Invesitgaciones Científicas (CSIC)-Universidad de León, Grulleros, 24346 León, Spain; mmarva@csic.es

**Keywords:** anthelmintic, chalcone, *trans*-chalcone, RNA-seq, mode of action, *Caenorhabditis elegans*

## Abstract

Chalcones, a subclass of flavonoid-derived phenolic compounds, have demonstrated promising anthelmintic activity against parasitic nematodes. This study aimed to obtain insights into the biological effects a *cis*/*trans* mixture of chalcone and its geometric isomer, *trans*-chalcone, using RNA sequencing in the model organism *Caenorhabditis elegans*. Fourth-stage larvae (L4) were exposed to *cis*/*trans*-chalcone or *trans*-chalcone for 3 h, and total RNA was extracted for high-throughput sequencing. Transcriptomic analysis revealed that exposure to *cis*/*trans*-chalcone and *trans*-chalcone induced pronounced modulation of genes involved in lipid metabolism and repression of collagen and structural genes, potentially leading to defective extracellular matrix maintenance, thereby suggesting these combined effects as potential mechanisms underlying their anthelmintic activity. Also, metabolic and stress response pathways, with several genes implicated in detoxification and cellular defense, were markedly upregulated. These findings provide new insights into the molecular mechanisms affected by chalcones, advancing our understanding of their anthelmintic potential and supporting future drug development efforts.

## 1. Introduction

**Figure 1:**
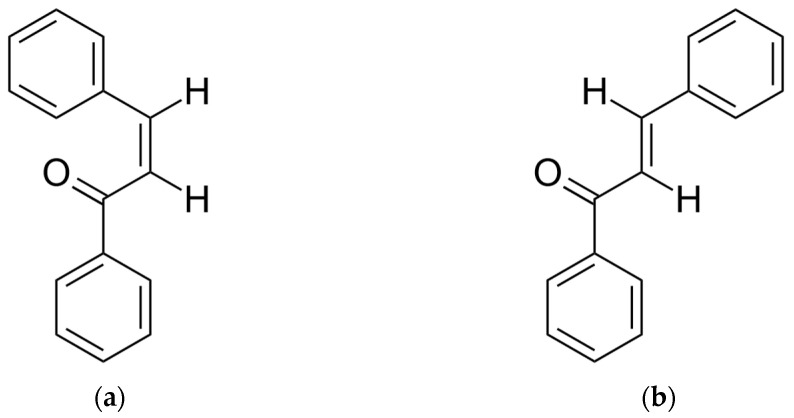
Chemical structure of the compounds studied: (**a**) *cis*-chalcone; (**b**) *trans*-chalcone. *Cis*-chalcone was only present in the *cis/trans* mixture.

Chalcones, a subclass of phenolic compounds within the flavonoid group, occur both naturally and synthetically. Structurally, they are defined by a 1,3-diaryl-2-propen-1-one backbone, a feature that underpins their diverse biological activities [1]. Extensive research has attributed a multitude of pharmacological properties to these compounds, including anticancer, antibiotic, antioxidant, anti-inflammatory and antimalarial effects, as well as ultraviolet radiation absorption [2,3,4,5]. Furthermore, the effect of chalcone derivatives as potential anthelmintic agents has been explored [6,7,8,9,10,11]. In a recent drug discovery campaign based on the screening of several natural product libraries, we identified the larvicidal potential of *cis*/*trans*-chalcone and its geometric isomer, *trans*-chalcone (Figure 1), against the free-living nematode *Caenorhabditis elegans*. Additionally, both compounds exhibited potent ovicidal activity against parasitic nematodes of ruminants, such as *Haemonchus contortus* and *Teladorsagia circumcincta* [12].

*Cis/trans*-chalcone was primarily isolated from the Chinese licorice plant *Glycyrrhiza inflata* and serves as a scaffold for the chemical synthesis of various chalcone derivatives. *Trans*-chalcone is sourced from the skin of *Aronia melanocarpa*, commonly known as black chokeberry, and represents a biphenolic core structure that is a precursor to flavonoids. Both isomers are characterized by relatively low molecular weights and moderate lipophilicity, properties that likely facilitate cellular uptake and enhance their overall bioactivity profiles [13].

In the context of anthelmintic research, *C. elegans* serves as a well-established model organism that does not require a host and is favored for its rapid generation time, ease of propagation, and well-annotated genome. Its metabolic and genetic landscapes are exceptionally well characterized; furthermore, many core metabolic, signaling, and genetic pathways are conserved across nematodes, including numerous parasitic species [14,15,16]. These characteristics facilitate the discovery of new anthelmintic compounds [17,18,19] including those whose mechanisms of action (MoAs) are not yet elucidated. In this regard, *C. elegans* has proven instrumental in deciphering the MoAs of all major classes of commercial anthelmintics [20,21,22,23,24,25,26,27]. Additionally, comparative studies indicate that compounds showing lethality in *C. elegans* often display similar effects against parasitic nematodes, suggesting a high degree of conserved metabolic pathways and genetic homology across species [28,29,30].

Despite the demonstrated anthelmintic potential of chalcones, their molecular MoA remains unexplored. Identifying the MoA of novel molecules is a critical milestone in drug development and is essential for advancing promising candidates toward commercialization. While some studies have reported the nematocidal activity of chalcones in *C. elegans*, these investigations have predominantly focused on phenotypic outcomes such as reduced motility, paralysis, and lethality, providing limited insight into the underlying molecular mechanisms [8]. Qian et al. used RNA-seq to characterize the transcriptomic response of *Cryptococcus neoformans*-infected *C. elegans* exposed to isobavachalcone. In their study, they found the chalcone derivative improved redox balance by increasing NADP^+^/NADPH and GSH/GSSG ratios, reducing intracellular Reactive Oxygen Species (ROS) and Fe^2+^ concentrations, ultimately leading to the inhibition of ferroptosis and an increase in the *C. elegans* survival rate after infection [31]. However, systems-level analyses investigating the effects of chalcone are currently lacking. Therefore, the present study aims to build upon our previous findings by providing deeper characterization of the biological effects of chalcones at the transcriptomic level. To achieve this, we compared the expression profiles of the *C. elegans* N2 strain exposed to *cis/trans*-chalcone, *trans*-chalcone or dimethyl sulfoxide (DMSO), the last serving as the negative control. This approach enables identification of the biological and metabolic processes affected by these compounds, thereby generating evidence that can guide subsequent functional studies aimed at further elucidating their mechanisms of action.

## 2. Results

### 2.1. Chalcone Isomers Induce Distinct Global Transcriptomic Responses in C. elegans

To obtain deeper insights into the biological effects of *cis/trans*-chalcone and *trans*-chalcone in the *C. elegans* N2 strain, pools of 2000 L4 larvae were exposed separately to a final concentration of 50 µM of each compound, or 0.05% DMSO as the negative control. Total isolated RNA exhibited high integrity, confirming its suitability for subsequent library preparation. The resulting RNA-seq libraries met stringent quality criteria, achieving Q30 scores above 96%, high mapping ratios, and 99.9% read accuracy, as confirmed by BMKGENE quality control analysis (Supplementary Figure S1). Sequencing saturation curves demonstrated comprehensive transcriptome coverage, enabling the reliable detection of mature mRNAs and alternative splicing events. These metrics confirm the high quality and robustness of the cDNA libraries utilized in this study (Supplementary Figures S2 and S3).

**Figure 2:**
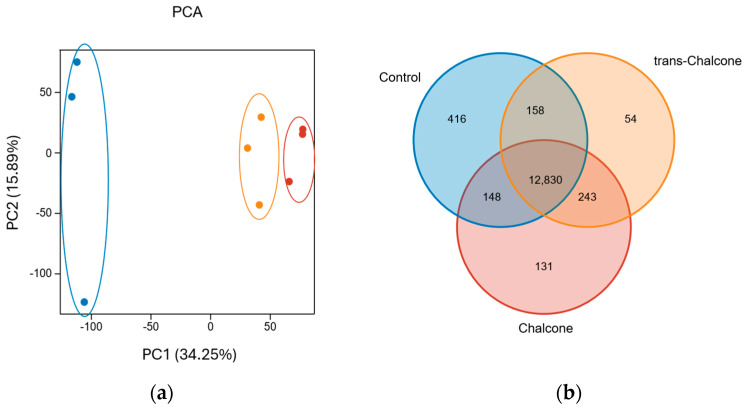
Transcriptomic landscapes of *C. elegans* following DMSO, *cis/trans*-chalcone or *trans*-chalcone exposure: (**a**) PCA plots showing the distinct segregation of DMSO-exposed controls (blue) from *cis/trans*-chalcone-exposed (red) and *trans*-chalcone-exposed (orange) samples (*n* = three biological replicates); (**b**) Venn diagram illustrating the distribution of shared and unique transcripts among experimental groups. While a core set of 12,830 genes was co-expressed across all conditions, a small subset of transcripts remained unique to the control group (3.2%) and to each respective chalcone exposure (1.0% and 0.4%).

Exposure of *C. elegans* L4 larvae to *cis*/*trans*-chalcone and *trans*-chalcone for a period of 3 h induced distinct transcriptional profiles, as evidenced by principal component analysis (PCA), which showed clear separation of control worms from both exposed groups along PC1 and PC2 (Figure 2a). These findings were further corroborated by Pearson correlation analysis (Supplementary Figure S4). Notably, the transcriptional profiles elicited by worms exposed to the *cis*/*trans* mix or the *trans* isomer were highly similar, indicating minimal divergence in their impact on gene expression. The Venn diagram in Figure 2b reveals a core set of 12,830 transcripts shared across all experimental groups. Furthermore, 416 transcripts (3.2%) were expressed exclusively in the control group, whereas 131 (1.0%) and 54 (0.4%) were uniquely detected following *cis*/*trans*-chalcone and *trans*-chalcone exposure, respectively.

**Figure 3:**
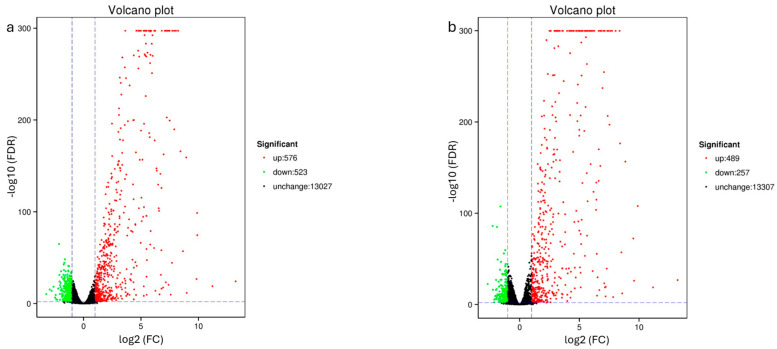
Volcano plot showing differentially expressed genes (DEGs) in *C. elegans*: (**a**) *cis*/*trans*-chalcone-exposed and (**b**) *trans*-chalcone-exposed worms versus negative controls. The *Y*-axis represents statistical significance (−log_10_ q-value), while the *X*-axis represents the magnitude of differential expression (log_2_ fold change). Black dots indicate genes that do not meet the significance thresholds; in contrast, green and red dots represent significantly downregulated or upregulated genes, respectively.

*Cis/trans*-chalcone induced a stronger transcriptional response than the *trans* isomer alone, as shown by the volcano plot analysis (Figure 3). Specifically, 1099 significantly modulated genes (FDR < 0.01) were identified following *cis*/*trans*-chalcone exposure—576 upregulated and 523 downregulated transcripts—out of the 14,126 detected. In contrast, *trans*-chalcone exposure yielded 746 differentially expressed genes (489 upregulated and 257 downregulated) among 14,033 transcripts. Genes upregulated in both exposure conditions displayed higher expression increases and higher statistical support under *cis*/*trans*-chalcone exposure, underscoring the more extensive transcriptional reprogramming elicited by the isomer mix.

### 2.2. Chalcone Isomers Promote Detoxification and Lipid Remodeling in C. elegans

**Figure 4:**
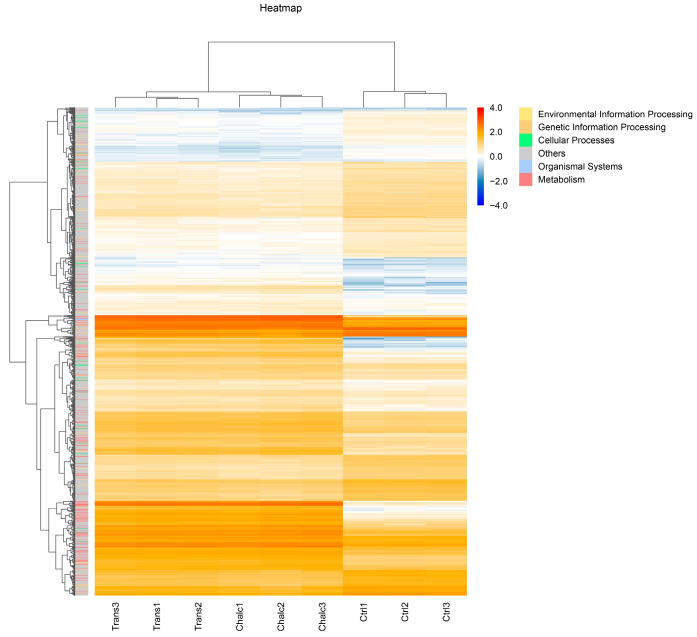
Heatmap of differential transcript expression and hierarchical clustering. The heatmap illustrates distinct expression profiles that clearly separate DMSO-exposed *C. elegans* L4 larvae (Ctrl) from larvae exposed to *cis*/*trans*-chalcone (Chalc) or *trans*-chalcone (Trans). Only minimal transcriptomic variations are detected between the Chalc and Trans isoforms, which exhibit high similarity in their expression patterns. The color scale represents relative abundance levels, ranging from low (blue) to high (red) expression. Dendrograms at the top and side reflect hierarchical clustering based on sample similarity and gene co-expression, respectively. The color-coded bar on the right categorizes transcripts into functional groups, including Metabolism, Genetic Information Processing, and Cellular Processes.

Hierarchical clustering analysis of the differential expression heatmap further confirmed a robust and exposure-specific transcriptional shift, with biological replicates exhibiting high consistency (R > 0.988), and both *cis*/*trans*-chalcone and *trans*-chalcone forming expression profiles that diverged significantly from the control group (Figure 4). Notably, in both cases a coordinated upregulation of gene clusters associated with the functional groups was induced, namely, Metabolism, Genetic Information Processing, and Organismal Systems, whereas the control group maintained a unique signature of 416 transcripts linked to the functional group Cellular Processes, which were strongly suppressed upon exposure. Collectively, these findings suggest that both *cis*/*trans*-chalcone and *trans*-chalcone trigger a specific metabolic shift in *C. elegans*, prioritizing the activation of metabolic pathways over baseline cellular maintenance.

Gene Ontology (GO) enrichment analysis revealed a highly similar transcriptional response in *C. elegans* exposed to 50 µM *cis*/*trans*-chalcone or *trans*-chalcone (Supplementary Figure S5). Upregulated differentially expressed genes (DEGs) were mainly associated with metabolic and cellular processes and were localized within intracellular components, particularly reflecting catalytic and binding activities (Supplementary Figure S5). In contrast, transcripts related to structural molecule activity were consistently downregulated.

**Figure 5:**
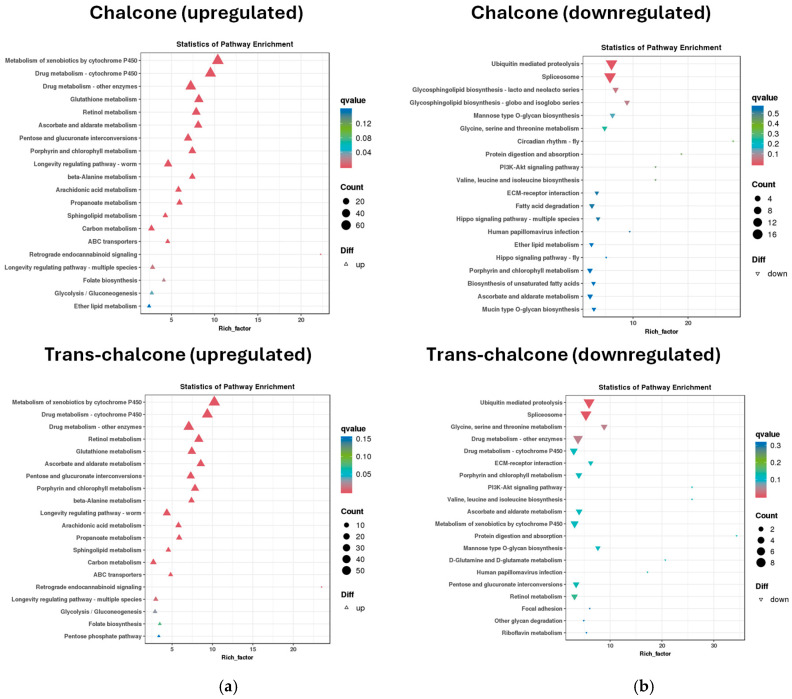
KEGG pathway enrichment analysis of *C. elegans* L4 larvae exposed with 50 μM *cis*/*trans*-chalcone and *trans*-chalcone: (**a**) enrichment of upregulated transcripts (enrichment factor > 7); (**b**) enrichment of downregulated transcripts. The *X*-axis represents the enrichment factor, indicated by triangles. Node size corresponds to the number of differentially expressed genes (DEGs), and node color indicates the statistical significance (q-value). All data are compared against the transcripts from DMSO-exposed *C. elegans* L4 larva.

KEGG enrichment analysis further showed that these upregulated genes particularly clustered in detoxification pathways (Figure 5a), including metabolic pathways for xenobiotics, retinol, ascorbate, and arachidonic acid (enrichment factor > 7). Inspection of the ten most highly upregulated genes (Table 1) revealed genes encoding a phase I cytochrome P450 isozyme (*cyp-14A3*) and a phase II glutathione S-transferase (*gst-37*), underscoring transcriptional activation of multiple tiers of the xenobiotic detoxification system. In parallel, several additional genes were markedly upregulated, including the oxidoreductase-encoding gene Y73C8C.10 and the short-chain dehydrogenase encoding gene *stdh-2*, pointing to substantial transcriptional remodeling of cellular redox metabolism.

Conversely, the KEGG enrichment analysis also showed that both *cis*/*trans*-chalcone and *trans*-chalcone exposure induced a significant downregulation of transcripts associated with structural molecule activity, particularly within the categories structural constituent of cuticle, extracellular space and integral component of membrane (Supplementary Figures S6 and S7). Consistent with this, six of the ten most strongly downregulated genes were involved in collagen metabolism (Table 2), a functional category classified under extracellular matrix in KEGG. This repression suggests a substantial suppression of cuticle-related structural maintenance, potentially leading to weakening of the nematode cuticle. KEGG enrichment analysis further revealed a coordinated inhibition of ubiquitin-mediated proteolysis and spliceosome pathways, which are involved in normal protein homeostasis, by respectively tagging protein for elimination and maturing pre-mRNA to mRNA [8,31]. This may indicate a suppression of protein turnover and RNA processing (Figure 5b). These findings suggest that chalcone isomers modulate genes associated with structural and proteolytic cellular components in *C. elegans*.

By examining the KEGG pathway maps, it appears that *cis*/*trans*-chalcone and *trans*-chalcone exposure affects several genes involved in cellular stress response. Pathways linked to ROS metabolism, including NADH:flavin oxidoreductase activity, were enriched, consistent with oxidative stress induction. This was accompanied by changes in RNAPII-associated transcriptional regulation, indicating a broader transcriptional reprogramming, perhaps in response to ROS. Importantly, downstream effects were also apparent, as extracellular matrix (ECM) and receptor interaction pathways, particularly those involving collagen-related transcripts, were significantly altered, indicating possible structural remodeling of the ECM. Despite the overlapping effect, *trans*-chalcone uniquely induced a pronounced activation of the peroxisomal matrix, suggesting an increase in peroxisome-dependent oxidative metabolism not observed with *cis*/*trans*-chalcone (Supplementary Figures S6–S9). Taken together, these data suggest a systemic metabolic reallocation in which cellular resources are shifted toward detoxification and stress adaptation at the expense of structural maintenance and routine proteostasis.

Lipid metabolic pathways were significantly modulated in both conditions (Figure 5, Supplementary Figures S8 and S9). This included upregulation of genes involved in sphingolipid metabolism, fatty acid (FA) biosynthesis and elongation, such as the FA synthase (FASN) gene, as well as enrichment of arachidonic acid, retinol and ether lipid metabolic pathways (Figures S8 and S9). The impact on FASN is particularly significant considering that this multienzyme complex catalyzes lipids synthesis from acetyl-CoA and malonyl-CoA, suggesting an upregulation of the entire lipid biosynthesis pathway. The upregulation of F12E12.12 (Table 1), a gene encoding an enoyl-reductase, further highlights the profound impact of these compounds on lipid metabolism, as this enzyme catalyzes the last step of the FA elongation process. Since both FASN and enoyl-reductase are NADPH-dependent enzymes, their upregulation may lead to depletion of these important cofactors, potentially resulting in oxidative stress. However, isomer-specific differences were observed: *cis*/*trans*-chalcone induced a downregulation or mixed regulation of FA degradation and unsaturated fatty acid (UFA) biosynthesis, while *trans*-chalcone promoted clear upregulation of FA degradation pathways and stronger induction of UFA biosynthesis. Furthermore, the highly upregulated gene, *gba-2* (Table 1)*,* suggests increased sphingolipid degradation and a compensatory response to lipid or oxidative stress [32]. In addition, *trans*-chalcone exerted a greater effect on genes related to glycerophospholipid metabolism, while *cis*/*trans*-chalcone showed comparatively stronger modulation of ether lipid pathways. The simultaneous activation of genes related to the lipid metabolic pathways and antioxidant systems indicates coordinated regulation of lipid-derived signaling molecules and redox homeostasis under chemical stress.

## 3. Discussion

Elucidating the MoAs of candidate anthelmintic compounds is a prerequisite for rational drug development. In the present study, we characterized the early transcriptional response of *C. elegans* L4 larvae following short-term exposure to *cis*/*trans*-chalcone and *trans*-chalcone, two flavonoid-related molecules previously shown to exert ovicidal and larvicidal activity against parasitic nematodes [12]. The 3 h exposure time was selected to capture early transcriptional responses to chalcone treatment while avoiding lethality. Global transcriptomic profiling revealed a robust and highly conserved response to both isomers. Principal component and hierarchical clustering analyses demonstrated a clear separation between DMSO-exposed control worms and worms exposed to either *cis*/*trans*-chalcone or *trans*-chalcone, whereas the chalcone *trans* isomers and the *cis*/*trans* mix clustered closely together, indicating that they elicit a largely shared transcriptional response. Nevertheless, *cis*/*trans*-chalcone induced a higher number of differentially expressed genes and greater fold-change amplitudes, suggesting a quantitatively stronger transcriptional impact, probably attributable to the presence of *cis*-chalcone, despite a lower toxicity reported in our previous study [12].

A central finding of this study is the strong upregulation of genes involved in the metabolism of xenobiotics. Among the most upregulated genes were *cyp-14A3*, a phase I cytochrome P450 enzyme, and *gst-37*, which encodes a glutathione S-transferase that catalyzes glutathione conjugation. Together, these genes represent major components of the xenobiotic detoxification machinery and protection against lipid peroxidation [33,34,35,36,37]. The marked overexpression of the *gst-37* and *cyp-14A3* genes indicates that chalcone is recognized as a chemical stressor that rapidly activates canonical defense programs. This transcriptional pattern is consistent with redox imbalance, further supported by the upregulation of oxidoreductases and NADH:flavin oxidoreductase-encoding gene in locus Y73C8C.10, which participates in cellular redox homeostasis [38]. Collectively, these data suggest that oxidative and electrophilic stress represent early molecular events following exposure. Such redox imbalance is likely to impair essential cellular functions, including mitochondrial activity and energy production, thereby contributing to the reduced motility and physiological decline observed in treated *Caenorhabditis elegans*. The rapid activation of detoxification pathways further indicates that chalcones are sensed as xenobiotic threats, triggering a protective response which may, however, become energetically costly and unsustainable over time.

Concomitantly, both *cis*/*trans*-chalcone and *trans*-chalcone induced a broad remodeling of the expression of genes involved in lipid metabolic pathways. Enrichment of FA biosynthesis, elongation, sphingolipid metabolism, and arachidonic acid metabolism suggest a systemic reorganization of membrane lipid homeostasis. These changes are reflected by the upregulation of F12E12.12, encoding an enoyl-reductase, a key reductive enzyme within fatty acid-modifying pathways, whose impairment is known to disrupt FA synthesis and compromise mitochondrial function [39,40,41,42] as well as *stdh-2* encoding short-chain dehydrogenase 2, which suggest increased oxidoreductase activity under chemical stress [43]. Such changes may reflect compensatory membrane repair mechanisms triggered by structural damage or altered lipid bilayer integrity. Notably, *trans*-chalcone preferentially upregulated FA degradation and peroxisomal-associated genes, suggesting enhanced β-oxidation and peroxisome-dependent oxidative metabolism. In contrast, *cis*/*trans*-chalcone exerted a comparatively stronger modulation of ether lipid pathways. These differences, although subtle at the global level, indicate isomer-specific metabolic rewiring that may influence the kinetics or severity of downstream damage. Although the function of K12D9.1 ORF (Table 1) remains poorly characterized, its overexpression has been associated with detoxification processes, such as the metabolism of thiol-reducing agents, suggesting a role in xenobiotic handling [44,45]. In addition, *trans*-chalcone only was able to induce transcripts related to glycosylceramidases. Disfunction of this group of enzymes is known to lead to lysosomal damage and accumulation of FA and sphingolipids, consistent with the peroxisomal matrix remodeling observed in pathway analyses [46]. Beyond structural remodeling, disruption of lipid homeostasis is expected to directly affect membrane fluidity, organelle integrity, and signaling processes. Altered mitochondrial membrane composition may compromise oxidative phosphorylation, further exacerbating energy deficits. The preferential activation of β-oxidation and peroxisomal pathways by *trans*-chalcone suggests increased lipid catabolism, which may initially compensate for the energy imbalance but ultimately contributes to elevated ROS production and cellular damage.

In contrast, downregulated genes displayed greater divergence between the isomer mix and the *trans*-chalcone and predominantly comprised transcripts with unknown functions. Notably, both *cis*/*trans*-chalcone and *trans*-chalcone consistently downregulated the transcription of several structural genes involved in collagen metabolism, including *col-43*, *col-127*, *col-130*, *col-145*, *col-157*, and *col-183* [47,48,49,50,51]. This coordinated repression may compromise cuticle integrity, potentially contributing to the lethality observed in *C. elegans*. In nematodes, the collagen-rich cuticle constitutes an essential protective barrier that ensures mechanical stability and maintains osmotic homeostasis [52,53,54]. Alterations in the cuticle of *H. contortus* have been described in the presence of aminochalcones [7], though the MoA remains unknown. Similarly, plant extracts rich in chalcone derivatives have been shown to cause cuticle detachment in the plant parasitic nematode *Meloidogyne incognita* [55]. Therefore, chalcone-induced transcriptional repression, combined with inhibition of ubiquitin-mediated proteolysis and spliceosome-associated pathways, may indicate disrupted protein turnover and impaired maintenance of the extracellular matrix in *C. elegans*. This coordinated repression is expected to compromise cuticle integrity, reducing mechanical resistance and increasing susceptibility to environmental stress. In nematodes, disruption of the collagen-rich cuticle can lead to impaired locomotion, osmotic imbalance, and increased vulnerability to external damage, all of which may directly contribute to organismal death. Activation of antioxidant and detoxification systems, combined with lipid metabolic remodeling, is consistent with a stress-adaptive state that attempts to counteract cumulative cellular damage. However, our previous phenotypic data demonstrated that lethality becomes evident around 24 h [12], indicating that compensatory responses are ultimately insufficient.

**Figure 6:**
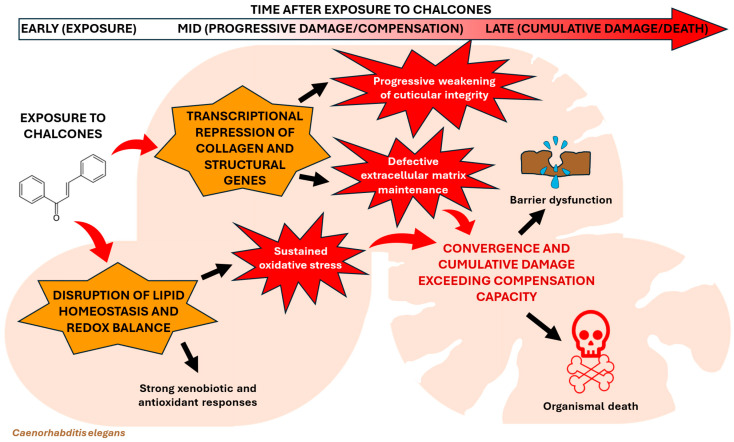
Proposed cumulative damage model of chalcone lethality in worms.

Integrating these observations, we propose a possible model (Figure 6) in which both *cis*/*trans*-chalcone and *trans*-chalcone initially disrupt lipid homeostasis and redox balance, triggering a strong xenobiotic and antioxidant response. Simultaneously, transcriptional repression of collagen and structural genes progressively weakens cuticular integrity. Over time, sustained oxidative stress and defective extracellular matrix maintenance likely converge, surpassing the worm’s compensatory capacity and culminating in barrier dysfunction and organismal death. The delayed onset of lethality is therefore compatible with a cumulative damage model rather than an acute neurotoxic mechanism, distinguishing chalcones from fast-acting ion channel-targeting anthelmintics [20,24,25].

The internal consistency of the transcriptomic dataset and the clear biological alignment of the affected pathways underscore the reliability of our results. Although targeted validation via RT-qPCR remains a valuable complementary approach for future research, the current systems-level analysis offers a comprehensive and valid overview of the molecular response to chalcones. A key limitation of this study is that the conclusions are primarily based on short-term transcriptomic responses rather than direct functional validation of the affected pathways. RNA sequencing after 3 h of exposure captures early gene expression changes but does not demonstrate whether the identified pathways, such as lipid metabolism remodeling, oxidative stress responses, or collagen repression, are causally responsible for the observed lethality. The use of an early time point was deliberate to isolate primary mechanisms from secondary lethal noise; as our model suggests, these early alterations pave the way for downstream structural impairment and subsequent pathways linked to lethality observed at later stages. In addition, transcript levels do not necessarily correlate with protein abundance or enzymatic activity, meaning that the physiological impact of these regulatory changes remains uncertain without proteomic or biochemical confirmation. For these reasons, future investigations should focus on experimentally validating the transcriptomic signatures identified and determining the primary cellular targets of chalcone exposure. Oxidative stress should be confirmed by measuring intracellular Reactive Oxygen Species using fluorescent probes, such as DCFH-DA or MitoSOX, combined with fluorescence microscopy or flow cytometry, alongside biochemical assays for lipid peroxidation, antioxidant enzyme activities, and glutathione redox balance. The lipid metabolic remodeling suggested by RNA-seq should be characterized through lipidomics, using liquid chromatography–mass spectrometry (LC-MS/MS) or gas chromatography–mass spectrometry (GC-MS) to quantify fatty acids, sphingolipids, and glycerophospholipids, complemented by Nile Red or BODIPY staining to visualize lipid accumulation in worms. Third, the proposed structural damage to the nematode cuticle should be verified using scanning or transmission electron microscopy and cuticle permeability assays with fluorescent dyes to detect extracellular matrix disruption. Lastly, functional validation of candidate genes implicated in detoxification and structural maintenance (e.g., collagen genes) should be performed using RNA interference or CRISPR-based gene knockouts, allowing assessment of whether perturbation of these pathways modifies susceptibility to chalcone and thereby clarifies the molecular mechanism underlying its anthelmintic activity. The framework proposed in Figure 6 establishes a robust foundation for future investigations into the molecular mechanisms of *cis/trans*-chalcone and *trans*-chalcone. By integrating these early transcriptomic shifts, our model provides a targeted roadmap for subsequent functional validation studies to confirm their translation into specific biological outcomes.

## 4. Materials and Methods

### 4.1. Test Compounds

*Cis*/*trans*-chalcone (cat. no. HY-121054) and *trans*-chalcone (cat. no. HY-Y0598) were sourced by MCE^®^ MedChemExpres (Monmouth Junction, NJ, USA). A 100 mM stock solution of the test compound was freshly prepared in 100% DMSO (Fisher Scientific^TM^, Waltham, MA, USA, D/4125/PB08) prior to use.

### 4.2. C. elegans Culture

The *C. elegans* N2 strain was obtained from the Caenorhabditis Genetics Center (CGC), University of Minnesota (Minneapolis, MN, USA). Worms were maintained under standard laboratory conditions on Nematode Growth Medium (NGM) plates and fed with *Escherichia coli* OP450. Synchronization of worm populations was carried out according to established protocols with hypochlorite to ensure uniform developmental staging [56]. Briefly, after bleaching adult worms, eggs were hatched overnight in M9 buffer to yield L1 larvae, which were transferred to *E. coli* OP50-seeded NGM plates and grown at 20 °C until reaching the L4 stage.

### 4.3. Compound Exposure and RNA Extraction from C. elegans L4

Synchronized L4 larvae were collected in M9 buffer and transferred to 15 mL conical tubes. Larvae were washed three times by centrifugation (2 min at 400× *g* at room temperature), discarding the supernatant after each cycle. After the final wash, larvae were pooled into a single tube and resuspended in M9 buffer. Larval density was assessed before distribution. Counting was carried out five times, and the average number of larvae was calculated. A total of 2000 L4 larvae were then incubated at 20 °C for 3 h in M9 solution and were exposed with *cis*/*trans*-chalcone or *trans*-chalcone at a final concentration of 50 µM (corresponding to approx. 1–2 × EC_50_ after 24 h) [12], while DMSO (0.05%) served as the control. The 3 h exposure period was specifically selected to investigate primary drug-induced transcriptional changes in the absence of mortality.

After incubation, the medium was discarded, and the larvae were washed with 1 mL of Milli Q water, followed by centrifugation and removal of the supernatant. Worm pellets were resuspended in 1 mL of TRIzol reagent (Invitrogen, Waltham, MA, USA) and transferred to homogenization tubes for mechanical lysis using a gentle MACS™ Octo Dissociator (Miltenyi Biotec, North Rhine-Westphalia, Germany) for 160 s. Phase separation was performed by adding 200 µL of chloroform, followed by vigorous shaking, incubation at room temperature for 3 min and centrifugation (15 min at 14,000× *g*, 4 °C). The aqueous phase was carefully transferred to new tubes and 500 µL of isopropanol was added to precipitate the RNA. The resulting RNA pellets were washed with 1 mL of ice-cold 70% ethanol, chilled at −20 °C for 1 h, centrifuged (5 min at 8000× *g*, 4 °C). After air-drying, pellets were then resuspended in RNase-free water preheated to 55 °C. RNA concentration and purity were assessed using a NanoDrop spectrophotometer (Thermo Fisher Scientific, Waltham, MA, USA). Samples containing at least 20 ng/µL (total RNA ≥ 0.5 µg) were used for subsequent analyses. RNA was stored at −80 °C until use.

### 4.4. RNA Sequencing and Bioinformatic Analysis

RNA sequencing was performed by BMKGENE (Münster, Germany). Total RNA concentration and purity were measured using a NanoDrop 2000 spectrophotometer, while RNA integrity was confirmed using the RNA Nano 6000 Assay Kit on an Agilent 2100 Bioanalyzer (Agilent Technologies, Santa Clara, CA, USA). Libraries were prepared using 1 μg of total RNA and the NEBNext Ultra™ RNA Library Prep Kit for Illumina (NEB, Ipswich, MA, USA). Following poly(A)+ mRNA enrichment with oligo(dT) magnetic beads, mRNA was fragmented, and reverse-transcribed to cDNA. After second-strand synthesis, end repair, and adaptor ligation, libraries were size-selected (~240 bp), and PCR amplified. Validated indexed libraries were clustered on a cBot system and sequenced using an Illumina platform in paired-end 150 bp mode (PE150) (Illumina, Inc., San Diego, CA, USA).

All bioinformatic analyses were performed using the BMKCloud platform. Raw reads in FASTQ format were processed to remove adapter sequences, poly-N stretches, and low-quality reads. Quality metrics including Q20, Q30, and GC content were calculated to ensure data integrity. Each sample comprised three technical replicates, with over 96% of transcripts surpassing the Q30 threshold in all cases. The resulting clean reads were aligned to the *C. elegans* reference genome (WBcel235) using HISAT2, and transcripts were assembled with StringTie, achieving mapping ratios between 97.85% and 98.88%. Gene expression levels were quantified as FPKM (Fragments Per Kilobase of transcript per Million mapped reads) (Figures S3 and S4). Alternative splicing prediction was carried out using Asprofile (Figure S5). Differential expression analysis was conducted using DESeq2 for datasets with biological replicates, or edgeR in their absence, applying a false discovery rate (FDR) threshold of *p* < 0.01. Genes with an adjusted *p*-value < 0.01 found by DESeq2 were assigned as differentially expressed, while FDR < 0.01 and fold change ≥ 2 were set as the threshold for significantly differential expression for the edgeR analysis. Subsequently, functional annotation and enrichment analyses (GO and KEGG) were performed on differentially expressed genes to identify significantly modulated biological pathways. Quality control analysis was performed in November 2025, and DEGs analysis was performed in December 2025.

## 5. Conclusions

In conclusion, our transcriptomic data indicate that *cis*/*trans*-chalcone and *trans*-chalcone share a conserved detoxification-centered mode of action characterized by oxidative stress induction, lipid metabolic reprogramming, and suppression of structural maintenance pathways. Isomer-specific differences in peroxisomal and fatty acid catabolic regulation suggest nuanced metabolic divergence that may influence efficacy or selectivity. These findings provide a mechanistic framework to guide further functional validation studies and support the continued development of chalcone-based scaffolds as potential anthelmintic agents.

## Figures and Tables

**Table 1:** Top upregulated genes after 3 h of exposure to *cis*/*trans*-chalcone or *trans*-chalcone compared to control (DMSO) in L4 *C. elegans*. Log_2_ (FC) indicates the fold change, while −log_10_ (FDR) indicates the statistical significance. n.s. indicate non significance.

	*cis*/*trans*-Chalcone		*trans*-Chalcone		
Gene	Log_2_ (FC)	−log_10_ (FDR)	Log_2_ (FC)	−log_10_ (FDR)	Function
*gst37*	13.23	6.74 × 10^−25^	13.23	1.89 × 10^−27^	Glutathione S-transferase
CB08E8.2	11.22	1.30 × 10^−19^	11.17	1.62 × 10^−19^	Unknown function
F12E12.12	9.90	3.14 × 10^−75^	9.52	4.81 × 10^−73^	Enoyl-reductase
E02C12.10	9.88	1.91 × 10^−99^	9.89	1.34 × 10^−108^	Unknown function
*stdh-2*	9.83	2.02 × 10^−27^	9.58	1.03 × 10^−26^	Short-chain dehydrogenase
K12D9.1	8.98	2.52 × 10^−12^	8.59	7.01 × 10^−13^	Unknown function
Y73C8C.10	8.94	6.54 × 10^−160^	8.85	2.54 × 10^−157^	NADH: flavin oxidoreductase
ZK697.14	8.65	9.34 × 10^−58^	8.52	7.48 × 10^−58^	Unknown function
*cyp-14A3*	8.44	1.46 × 10^−166^	8.41	3.32 × 10^−177^	Cytochrome P450 (CYP)
*gba-2*	8.23	n.s.	8.37	3.55 × 10^−40^	Glycosylceramidases

**Table 2:** Top downregulated genes in L4 *C. elegans* following 3 h of exposure to *cis*/*trans*-chalcone or *trans*-chalcone, relative to the DMSO control. Log_2_ (FC) represents the fold change, and −log_10_ (FDR) denotes statistical significance. n.s. indicate non significance.

	*cis/trans*-Chalcone		*trans*-Chalcone		
Gene	Log_2_ (FC)	−log_10_ (FDR)	Log_2_ (FC)	−log_10_ (FDR)	Function
*col-127*	−1.77	3.54 × 10^−4^	−1.46	0.004	Collagen metabolism
*col-183*	−2.04	7.79 × 10^−5^	−0.53	n.s.	Collagen metabolism
*col-157*	−1.63	1.74 × 10^−5^	−1.50	3.58 × 10^−5^	Collagen metabolism
*col-145*	−2.37	3.09 × 10^−5^	−1.67	0.001	Collagen metabolism
*col-43*	−0.45	n.s.	−1.70	3.08 × 10^−6^	Collagen metabolism
*col-130*	−2.77	1.42 × 10^−14^	−2.25	9.20 × 10^−87^	Collagen metabolism
C54C8.4	−2.94	5.25 × 10^−16^	−2.01	2.91 × 10^−10^	Unknown function
F48C1.11	−3.24	4.73 × 10^−11^	−1.23	0.002	Unknown function
ZC168.2	−2.61	5.47 × 10^−19^	−2.67	3.33 × 10^−23^	Unknown function
C07A4.2	−2.21	4.25 × 10^−17^	−2.23	6.18 × 10^−17^	Unknown function

## Data Availability

Raw data available in the Zenodo repository: https://doi.org/10.5281/zenodo.18657646.

## Supplementary Materials

The following supporting information can be downloaded at https://www.mdpi.com/article/10.3390/molecules31091411/s1, Supplementary Figure S1. Distribution of insert lengths across each sample for each condition in triplicate. Supplementary Figure S2. Saturation test for library volume adequacy. Supplementary Figure S3. Alternative splicing was predicted using Asprofile. Supplementary Figure S4. Pearson correlation heatmap of transcriptomic profiles. Supplementary Figure S5. Gene Ontology classification of differentially expressed genes (DEGs). Supplementary Figure S6. Enriched KEGG pathways of chalcone-exposed worms. Supplementary Figure S7. Enriched KEGG pathways of *trans*-chalcone-exposed worms. Supplementary Figure S8. Enriched KEGG pathways of chalcone-exposed worms. Supplementary Figure S9. Enriched KEGG pathways of trans-chalcone-exposed worms.

## Abbreviations

MoA	Mechanism of Action
L4	Larval stage 4
PCA	Principal Component Analysis
KEGG	Kyoto Encyclopedia of Genes and Genomes
GO	Gene Ontology
CYP	Cytochrome P450
eggNOG	Evolutionary genealogy of genes: Non-supervised Orthologous Groups
ROS	Reactive Oxygen Species
RNAP	RNA Polymerase
FASN	Fatty Acid Synthase
FA	Fatty Acids
UFA	Unsaturated Fatty Acids
ECM	Extracellular Matrix
EC_50_	Effective Concentration 50
CGC	Caenorhabditis Genetics Centre
