# Supplementary material for: Chalcone and Trans-Chalcone Induce Transcriptomic Changes in Caenorhabditis elegans Compatible with a Novel Cumulative Damage Mode of Action
Source: Molecules. 2026 Apr 24;31(9):1411. doi: 10.3390/molecules31091411 (PMC13164716; doi:10.3390/molecules31091411)
Supplement: Supplementary file 1 [file molecules-31-01411-s001.zip › molecules-4232951-supplementary.pdf]

Chalcone and Trans-Chalcone Induce Transcriptomic Changes in Caenorhabsitis elegans Compatible with a Novel Cumulative Damage Mode of Action

Giulio Galli [1], Carl S. Bruun [2], Carlos García-Estrada [1,3], Rafael Balaña-Fouce [1,3,*], María Martinez-Valladares [4] and Tina V. A. Hansen [2,*]

1 Departamento de Ciencias Biomédicas, Facultad de Veterinaria, Universidad de León, Campus de Vegazana s/n, 24071 León, Spain; ggal@unileon.es (G.G.); cgare@unileon.es (C.G.-E.)
2 Department of Veterinary and Animal Sciences, University of Copenhagen, Frederiksberg C, 1870 Copenhagen, Denmark; casb@sund.ku.dk
3 Instituto de Biomedicina (IBIOMED), Universidad de León, Campus de Vegazana s/n, 24071 León, Spain
4 Departamento Sanidad Animal, Instituto de Ganadería de Montaña, Conesjo Superior de Invesitgaciones Científicas (CSIC)-Universidad de León, Grulleros, 24346 León, Spain; mmarva@csic.es

* Correspondence: rbalf@unileon.es (R.B.-F.); alstrup@sund.ku.dk (T.V.A.H.)

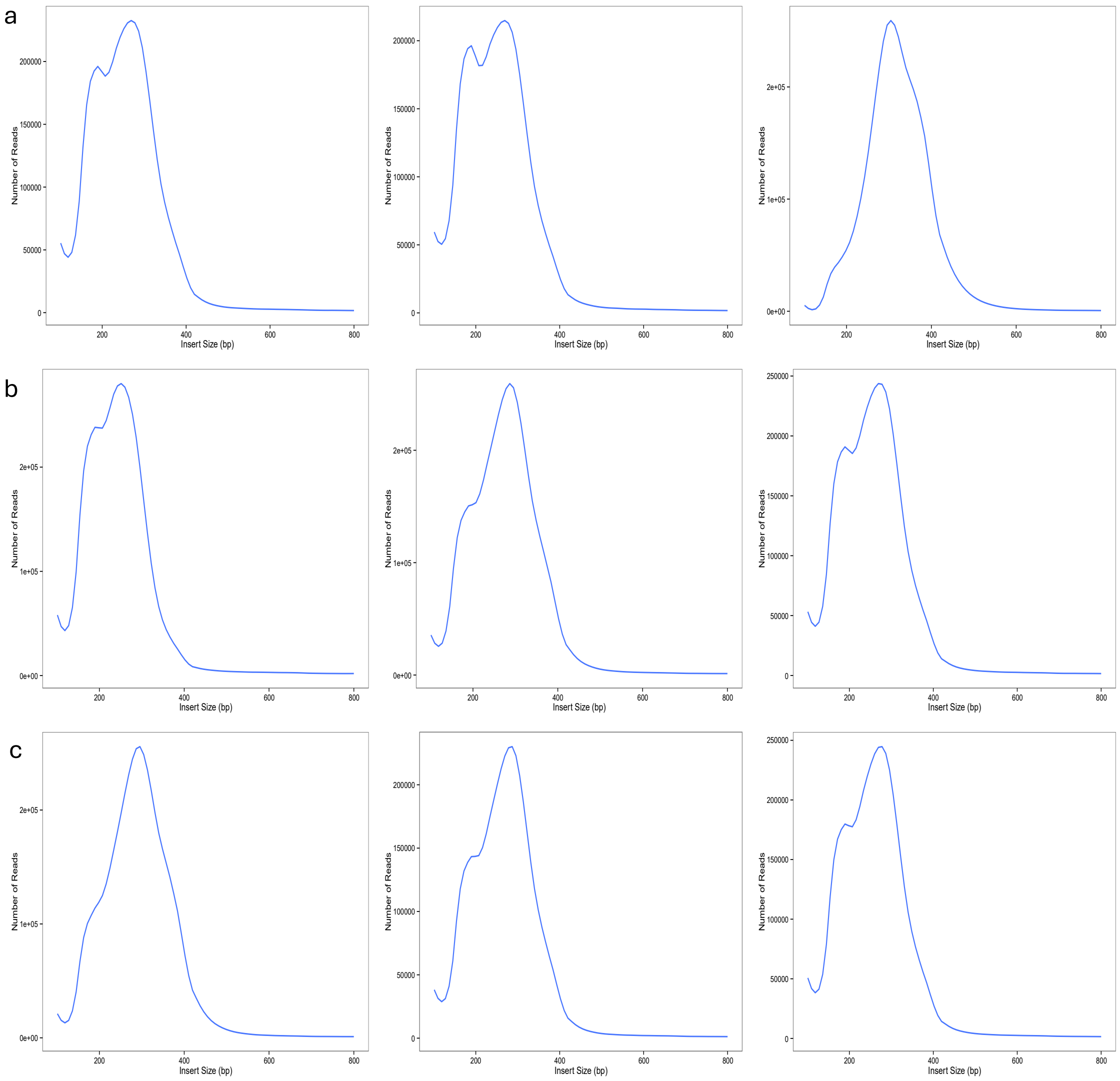

Supplementary Figure S1. Distribution of insert lengths across each sample for each condition in triplicate. The lack of multiple peaks suggests high-quality mature mRNA sequencing. a) control samples b) chalcone-exposed sample c) *trans*-chalcone-exposed sample.

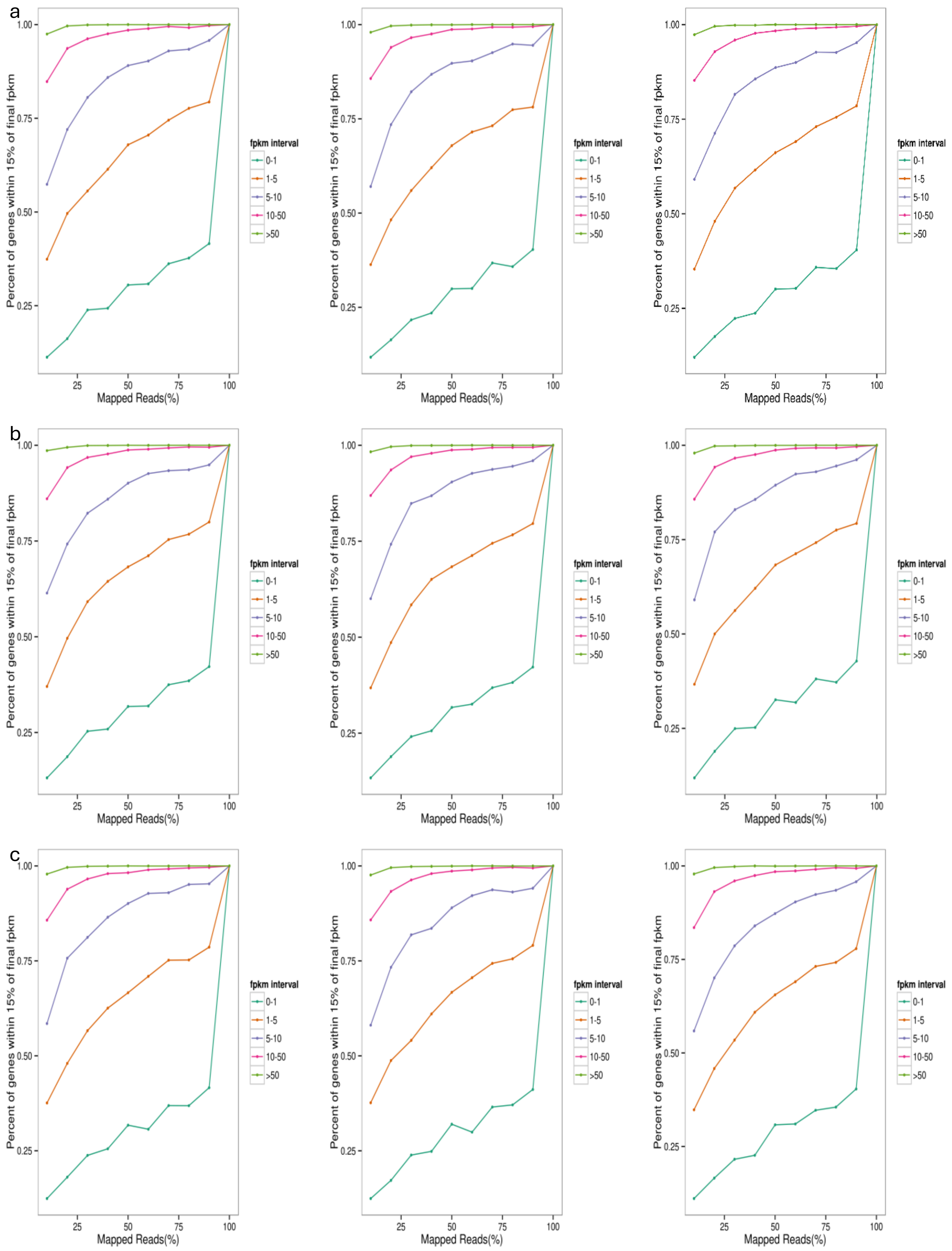

Supplementary Figure S2. Saturation test for library volume adequacy demonstrating extensive transcript coverage. a) control samples b) chalcone-exposed sample c) *trans*-chalcone-exposed sample, each line corresponding to triplicates.

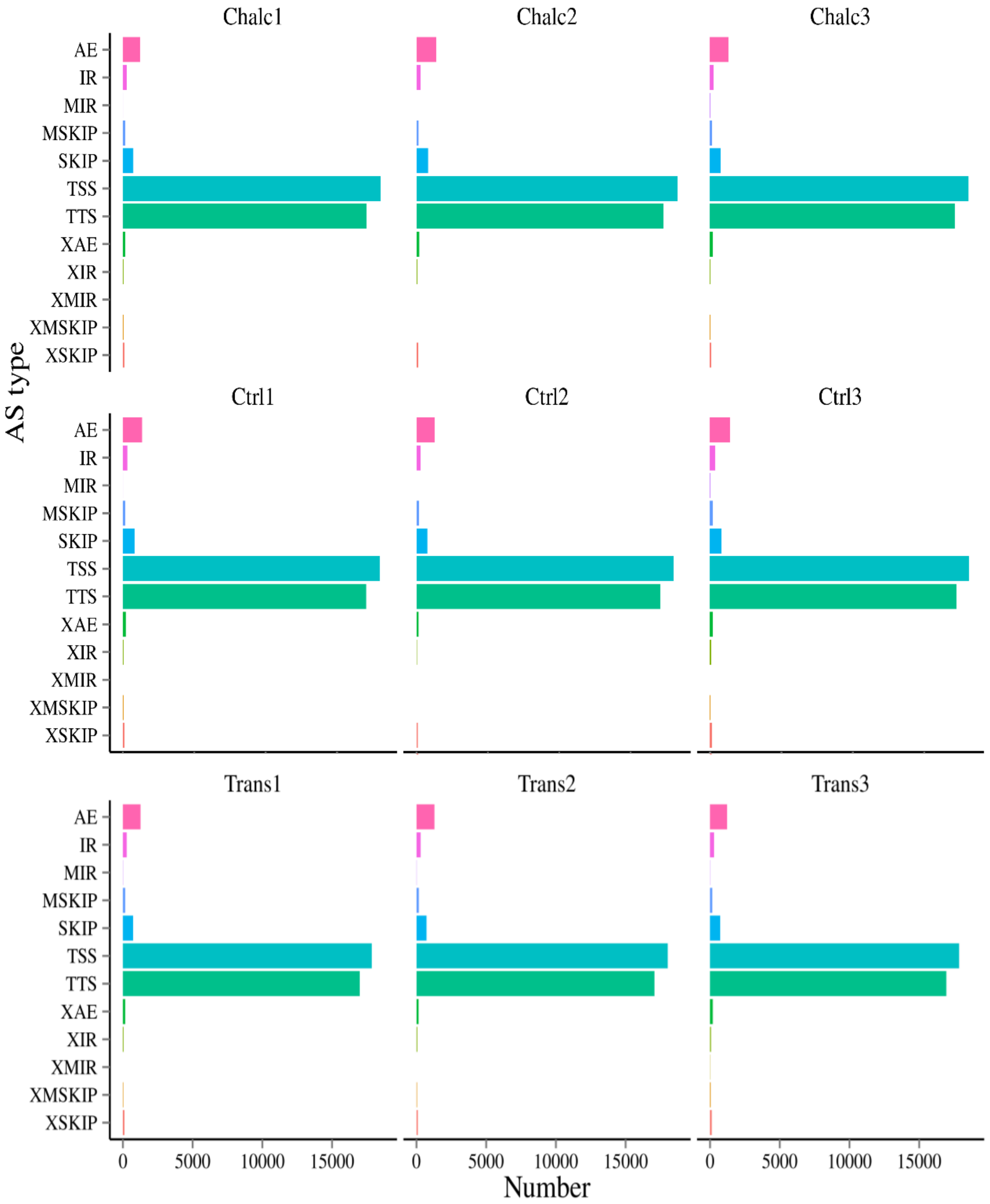

Supplementary Figure S3. Alternative splicing was predicted using Asprofile, highlighting differences among prediction models. Chalc = chalcone, ctrl = controls, trans = *trans*-chalcone, each line representing triplicates of the same sample.

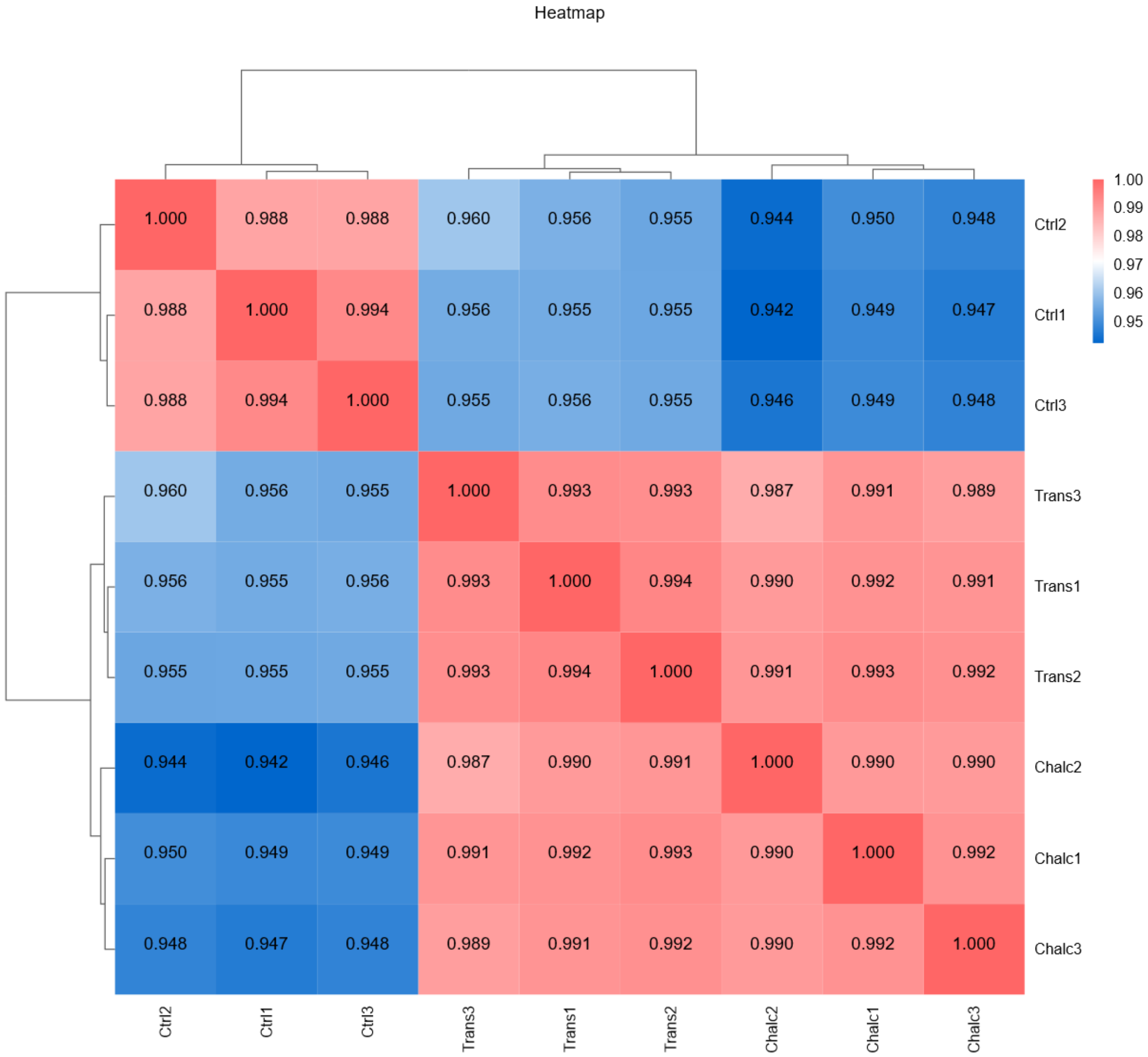

Supplementary Figure S4. Pearson correlation heatmap of transcriptomic profiles from *C. elegans* L4 larvae exposed to DMSO (controls, Ctrl), chalcone (Chalc), or *trans*-chalcone (Trans). A clear separation in transcriptomic profiles is observed between the DMSO-exposed larvae and those exposed to chalcone or *trans*-chalcone, indicating distinct global expression patterns, while more subtle differences are evident between the two chalcone isoforms.

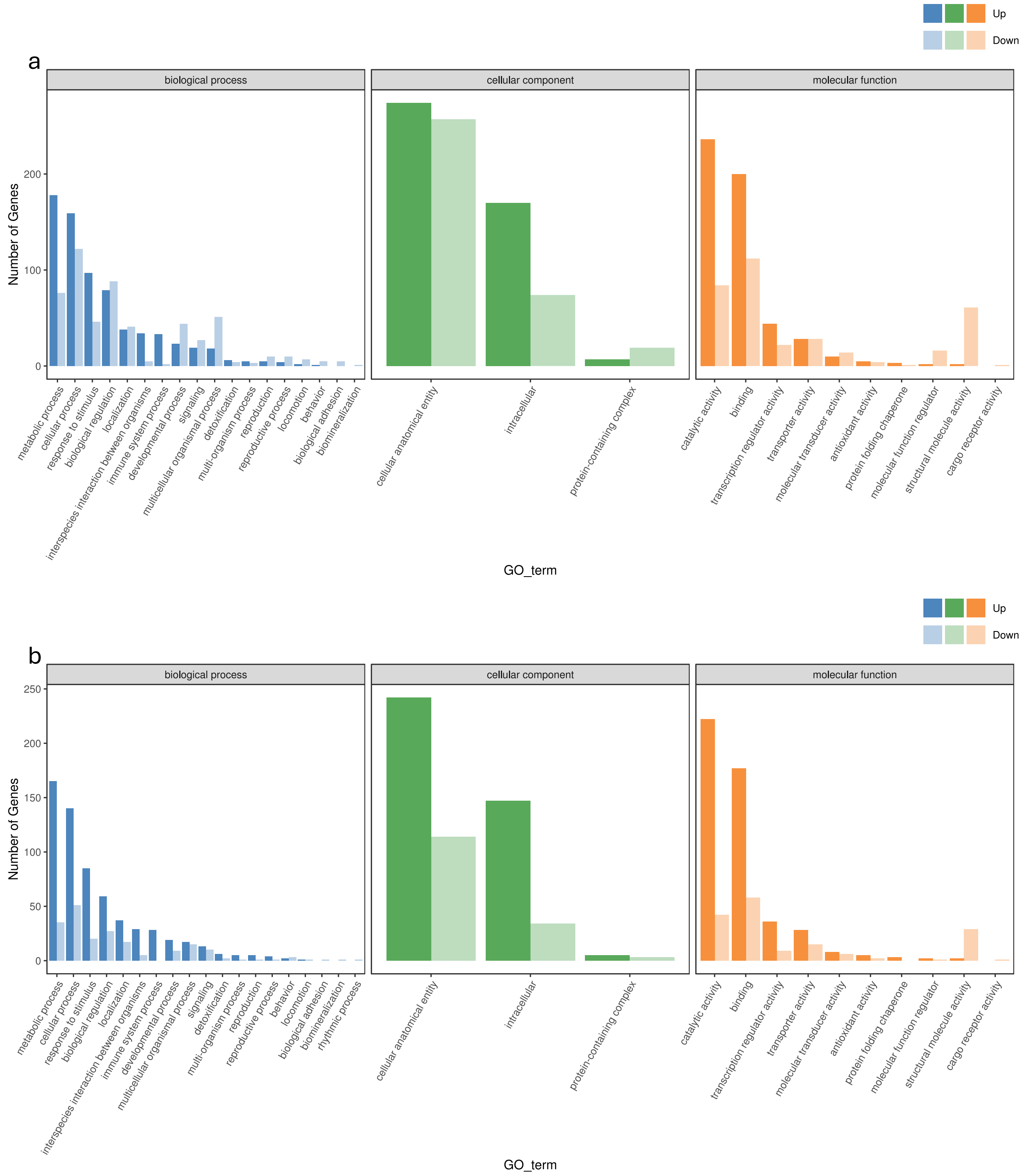

Supplementary Figure S5. Gene Ontology classification of differentially expressed genes (DEGs) in chalcone- or *trans*-chalcone-exposed *C. elegans* L4 larvae compared with DMSO-exposed control *C. elegans*, showing reduced enrichment of structural molecule activity and increased enrichment of metabolic and cellular processes. a) chalcone-exposed L4 larvae, b) *trans*-chalcone-exposed L4 larvae.

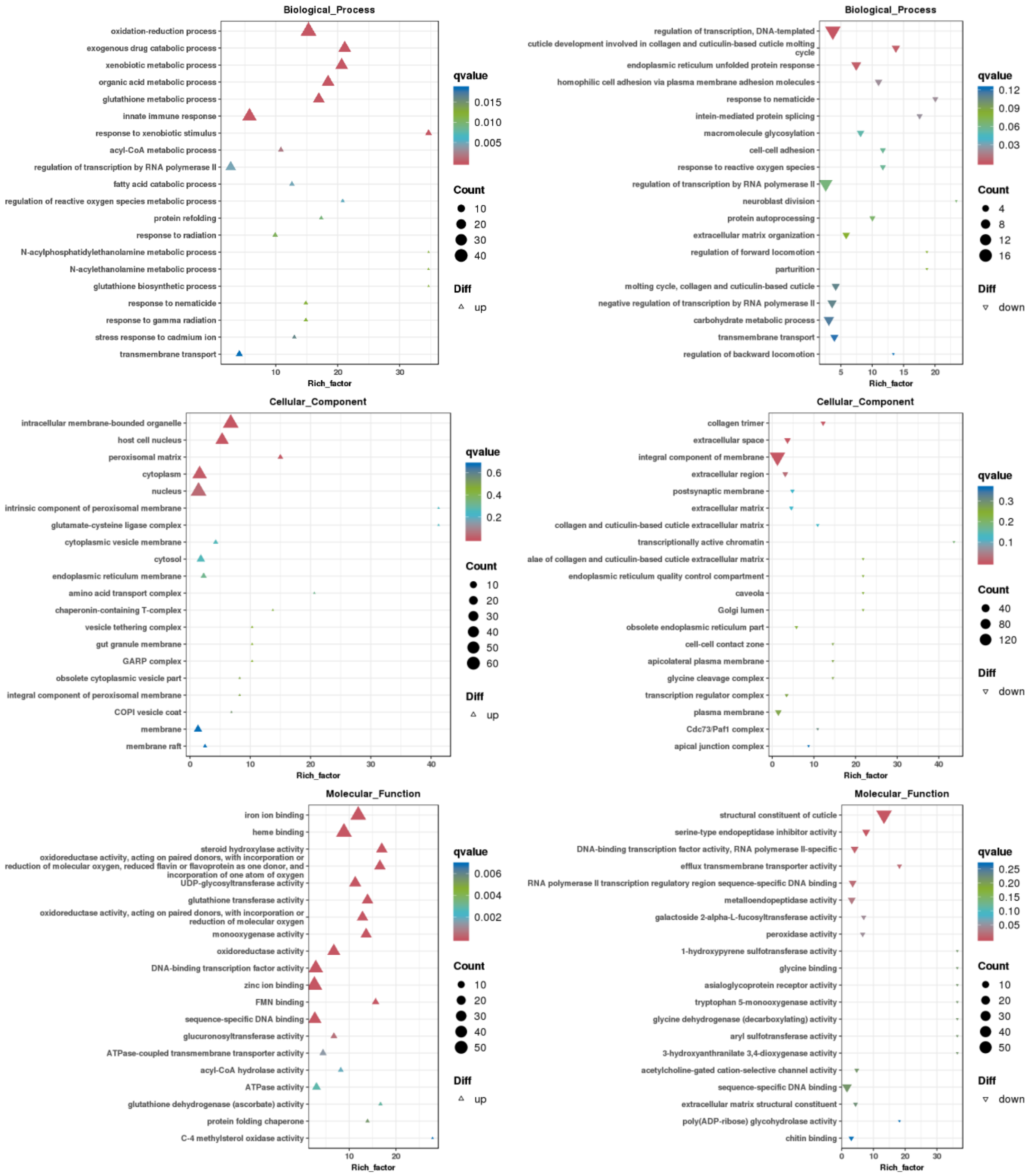

Supplementary Figure S6. Enriched KEGG pathways of chalcone-exposed *C. elegans* L4 larvae compared to DMSO-exposed control L4 larvae. Red: upregulated, green: downregulated, blue: both upregulated and downregulated.

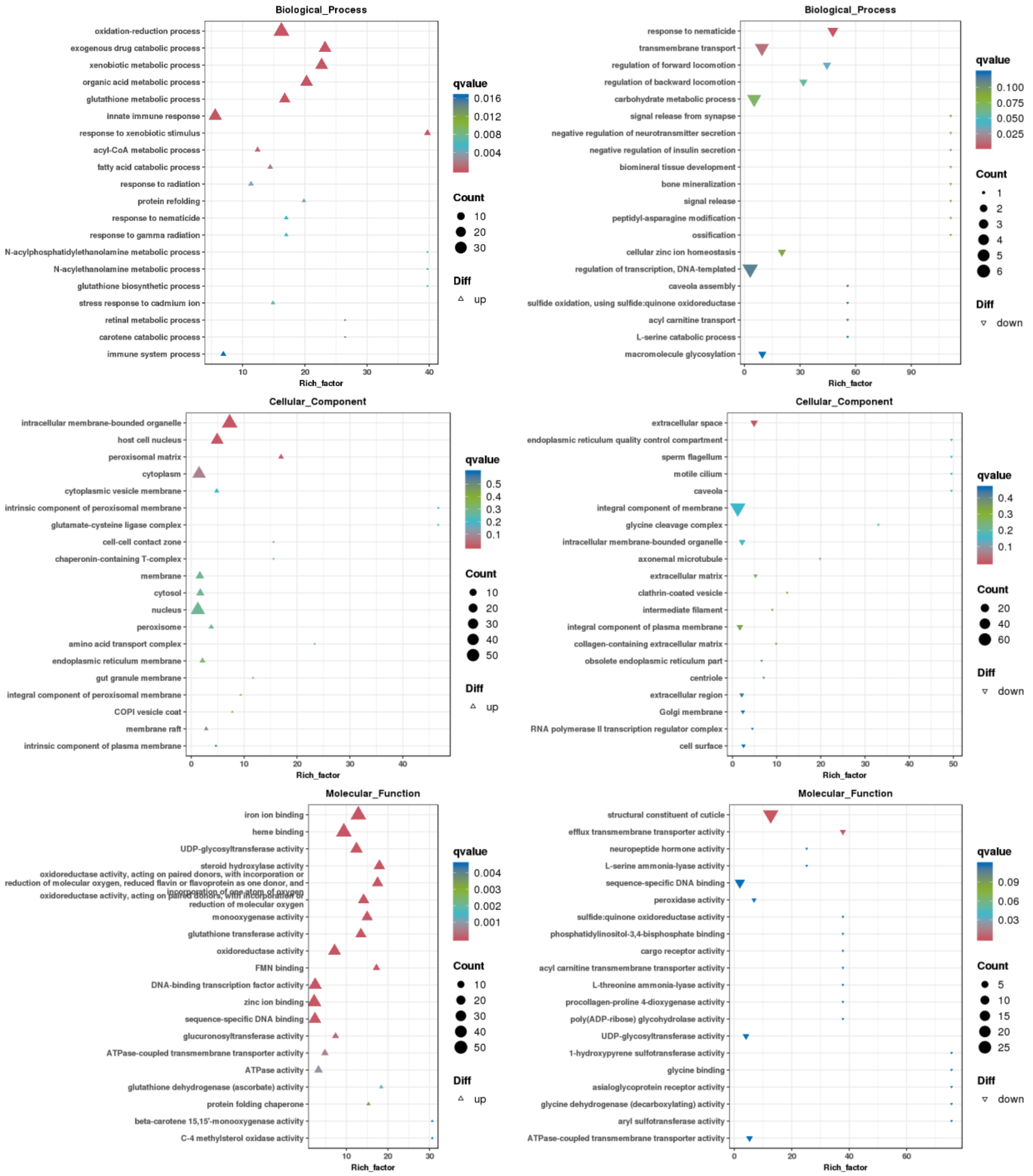

Supplementary Figure S7. Enriched KEGG pathways of *trans*-chalcone-exposed *C. elegans* L4 larvae compared to DMSO-exposed control L4 larvae. Red: upregulated, green: downregulated, blue: both upregulated and downregulated.

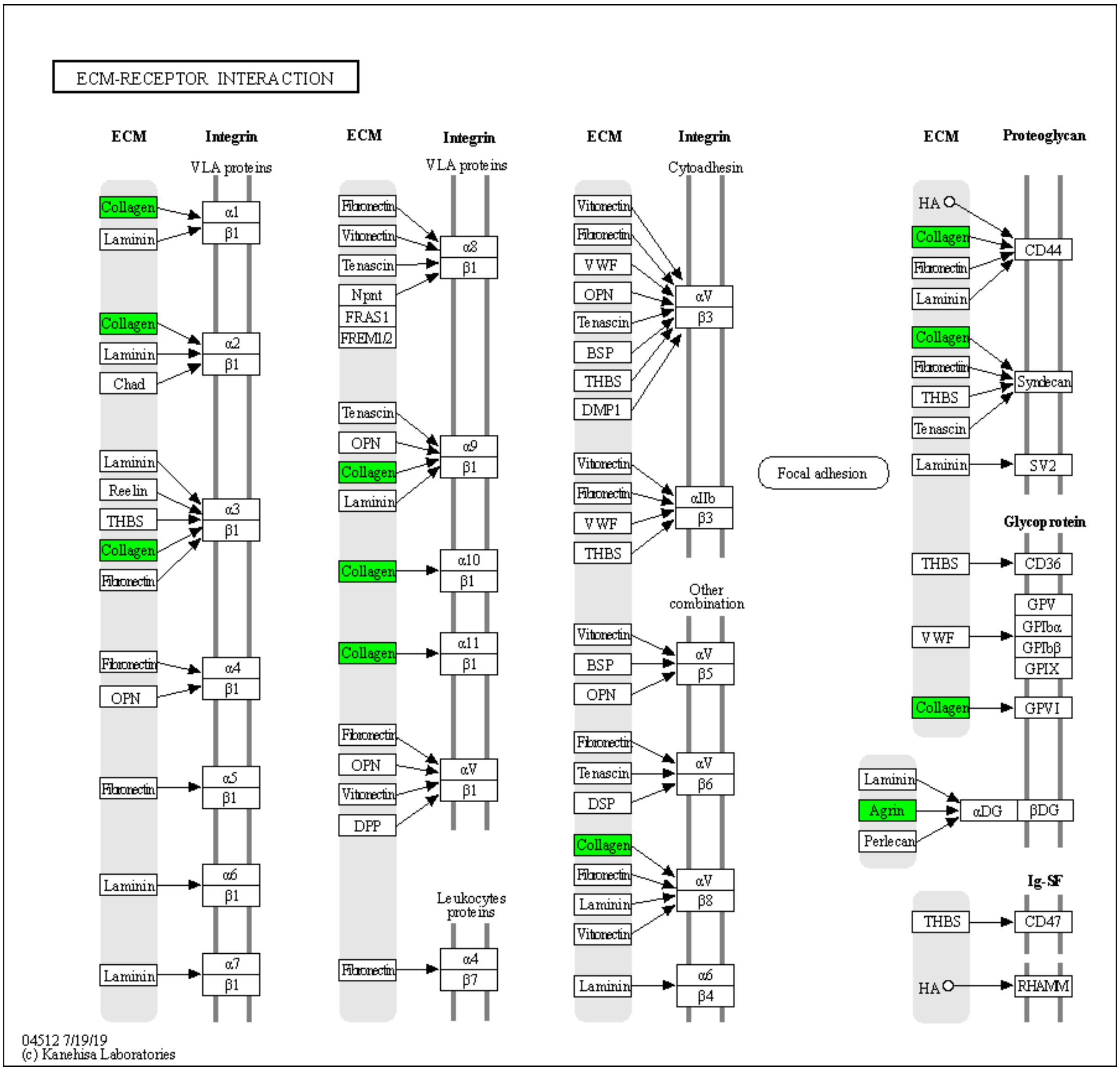

ECM-RECEPTOR INTERACTION
ECM
Integrin
VLA proteins
Collagen
Laminin
Chad
Reelin
THBS
Fibronectin
OPN
Vitronectin
Tenascin
Npnt
FRAS1
FREM1/2
DPP
Leukocytes proteins
Cytoadhesin
VWF
BSP
DMP1
Other combination
DSP
Focal adhesion
Proteoglycan
HA
CD44
Syndecan
SV2
Glycoprotein
CD36
GPV
GPIbα
GPIbβ
GPIX
GPVI
Agrin
Perlecan
αDG
βDG
Ig-SF
CD47
RHAMM
04512 7/19/19
(c) Kanehisa Laboratories

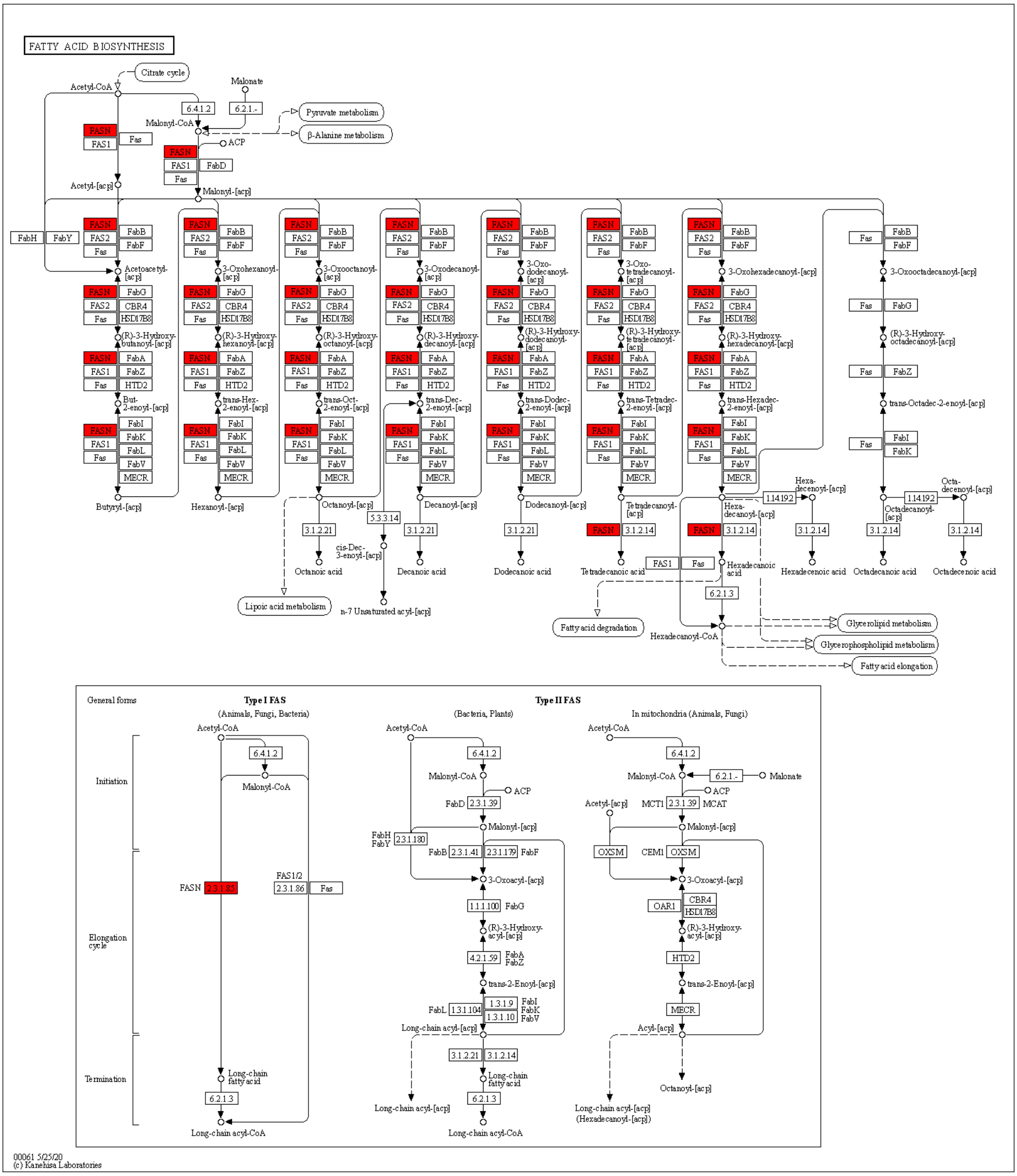

FATTY ACID BIOSYNTHESIS
Citrate cycle
Acetyl-CoA
Malonate
6.4.1.2
6.2.1.-
Pyruvate metabolism
β-Alanine metabolism
Malonyl-CoA
ACP
FASN
FAS1
Fas
FabD
Acetyl-[acp]
Malonyl-[acp]
FabH
FabY
FAS2
FabB
FabF
FabG
CBR4
HSD17B8
FabA
FabZ
HTD2
FabI
FabK
FabL
FabV
MECR
Acetoacetyl-[acp]
(R)-3-Hydroxy-butanoyl-[acp]
But-2-enoyl-[acp]
Butyryl-[acp]
3-Oxohexanoyl-[acp]
(R)-3-Hydroxy-hexanoyl-[acp]
trans-Hex-2-enoyl-[acp]
Hexanoyl-[acp]
3-Oxooctanoyl-[acp]
(R)-3-Hydroxy-octanoyl-[acp]
trans-Oct-2-enoyl-[acp]
Octanoyl-[acp]
3-Oxodecanoyl-[acp]
(R)-3-Hydroxy-decanoyl-[acp]
trans-Dec-2-enoyl-[acp]
Decanoyl-[acp]
3-Oxo-dodecanoyl-[acp]
(R)-3-Hydroxy-dodecanoyl-[acp]
trans-Dodec-2-enoyl-[acp]
Dodecanoyl-[acp]
3-Oxo-tetradecanoyl-[acp]
(R)-3-Hydroxy-tetradecanoyl-[acp]
trans-Tetradec-2-enoyl-[acp]
Tetradecanoyl-[acp]
3-Oxohexadecanoyl-[acp]
(R)-3-Hydroxy-hexadecanoyl-[acp]
trans-Hexadec-2-enoyl-[acp]
Hexa-decanoyl-[acp]
3-Oxooctadecanoyl-[acp]
(R)-3-Hydroxy-octadecanoyl-[acp]
trans-Octadec-2-enoyl-[acp]
Octadecanoyl-[acp]
Hexa-decenoyl-[acp]
Octa-decenoyl-[acp]
1.14.19.2
3.1.2.21
3.1.2.14
5.3.3.14
cis-Dec-3-enoyl-[acp]
Octanoic acid
Decanoic acid
Dodecanoic acid
Tetradecanoic acid
Hexadecanoic acid
Hexadecenoic acid
Octadecanoic acid
Octadecenoic acid
Lipoic acid metabolism
n-7 Unsaturated acyl-[acp]
6.2.1.3
Fatty acid degradation
Hexadecanoyl-CoA
Glycerolipid metabolism
Glycerophospholipid metabolism
Fatty acid elongation
General forms
Type I FAS
(Animals, Fungi, Bacteria)
Type II FAS
(Bacteria, Plants)
In mitochondria (Animals, Fungi)
Initiation
Elongation cycle
Termination
FAS1/2
2.3.1.85
2.3.1.86
Long-chain fatty acid
Long-chain acyl-CoA
FabD 2.3.1.39
FabH FabY 2.3.1.180
FabB 2.3.1.41
2.3.1.179 FabF
3-Oxoacyl-[acp]
1.1.1.100 FabG
(R)-3-Hydroxy-acyl-[acp]
4.2.1.59 FabA FabZ
trans-2-Enoyl-[acp]
FabL 1.3.1.104
1.3.1.9
1.3.1.10
Long-chain acyl-[acp]
MCT1 2.3.1.39 MCAT
OXSM
CEM1
OAR1
Acyl-[acp]
Octanoyl-[acp]
Long-chain acyl-[acp] (Hexadecanoyl-[acp])
00061 5/25/20
(c) Kanehisa Laboratories

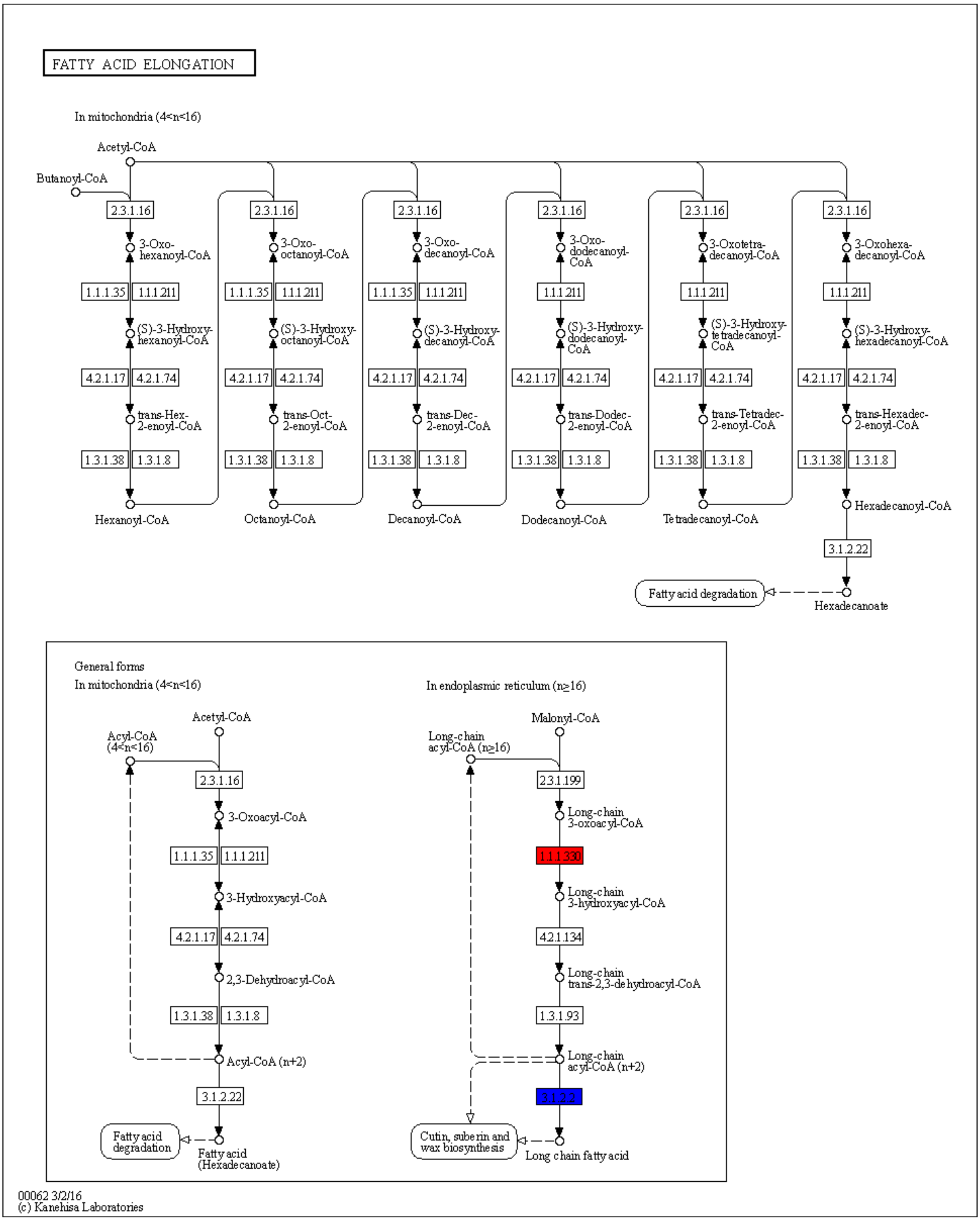
FATTY ACID ELONGATION
In mitochondria (4<n<16)
Acetyl-CoA
Butanoyl-CoA
2.3.1.16
3-Oxo-hexanoyl-CoA
3-Oxo-octanoyl-CoA
3-Oxo-decanoyl-CoA
3-Oxo-dodecanoyl-CoA
3-Oxotetra-decanoyl-CoA
3-Oxohexa-decanoyl-CoA
1.1.1.35
1.1.1.211
(S)-3-Hydroxy-hexanoyl-CoA
(S)-3-Hydroxy-octanoyl-CoA
(S)-3-Hydroxy-decanoyl-CoA
(S)-3-Hydroxy-dodecanoyl-CoA
(S)-3-Hydroxy-tetradecanoyl-CoA
(S)-3-Hydroxy-hexadecanoyl-CoA
4.2.1.17
4.2.1.74
trans-Hex-2-enoyl-CoA
trans-Oct-2-enoyl-CoA
trans-Dec-2-enoyl-CoA
trans-Dodec-2-enoyl-CoA
trans-Tetradec-2-enoyl-CoA
trans-Hexadec-2-enoyl-CoA
1.3.1.38
1.3.1.8
Hexanoyl-CoA
Octanoyl-CoA
Decanoyl-CoA
Dodecanoyl-CoA
Tetradecanoyl-CoA
Hexadecanoyl-CoA
3.1.2.22
Fatty acid degradation
Hexadecanoate
General forms
In mitochondria (4<n<16)
Acetyl-CoA
Acyl-CoA (4<n<16)
2.3.1.16
3-Oxoacyl-CoA
1.1.1.35
1.1.1.211
3-Hydroxyacyl-CoA
4.2.1.17
4.2.1.74
2,3-Dehydroacyl-CoA
1.3.1.38
1.3.1.8
Acyl-CoA (n+2)
3.1.2.22
Fatty acid degradation
Fatty acid (Hexadecanoate)
In endoplasmic reticulum (n≥16)
Malonyl-CoA
Long-chain acyl-CoA (n≥16)
2.3.1.199
Long-chain 3-oxoacyl-CoA
1.1.1.330
Long-chain 3-hydroxyacyl-CoA
4.2.1.134
Long-chain trans-2,3-dehydroacyl-CoA
1.3.1.93
Long-chain acyl-CoA (n+2)
3.1.2.2
Cutin, suberin and wax biosynthesis
Long chain fatty acid
00062 3/2/16
(c) Kanehisa Laboratories

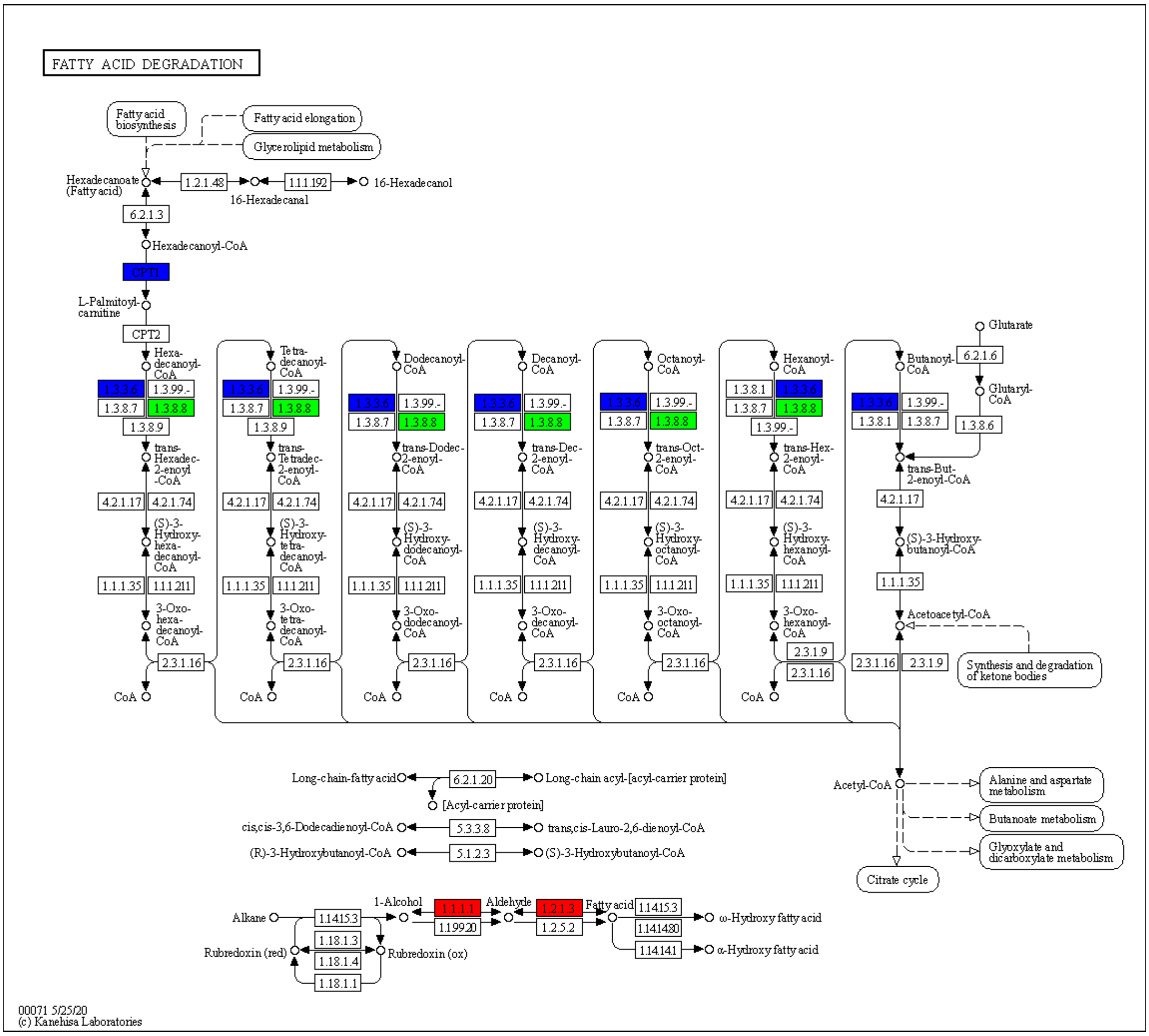
FATTY ACID DEGRADATION
Fatty acid biosynthesis
Fatty acid elongation
Glycerolipid metabolism
Hexadecanoate (Fatty acid)
1.2.1.48
16-Hexadecanal
1.1.1.192
16-Hexadecanol
6.2.1.3
Hexadecanoyl-CoA
CPT1
L-Palmitoyl-carnitine
CPT2
Hexa-decanoyl-CoA
Tetra-decanoyl-CoA
Dodecanoyl-CoA
Decanoyl-CoA
Octanoyl-CoA
Hexanoyl-CoA
Butanoyl-CoA
Glutarate
6.2.1.6
Glutaryl-CoA
1.3.8.6
1.3.3.6
1.3.99.-
1.3.8.7
1.3.8.8
1.3.8.9
1.3.8.1
trans-Hexadec-2-enoyl-CoA
trans-Tetradec-2-enoyl-CoA
trans-Dodec-2-enoyl-CoA
trans-Dec-2-enoyl-CoA
trans-Oct-2-enoyl-CoA
trans-Hex-2-enoyl-CoA
trans-But-2-enoyl-CoA
4.2.1.17
4.2.1.74
(S)-3-Hydroxy-hexa-decanoyl-CoA
(S)-3-Hydroxy-tetra-decanoyl-CoA
(S)-3-Hydroxy-dodecanoyl-CoA
(S)-3-Hydroxy-decanoyl-CoA
(S)-3-Hydroxy-octanoyl-CoA
(S)-3-Hydroxy-hexanoyl-CoA
(S)-3-Hydroxy-butanoyl-CoA
1.1.1.35
1.1.1.211
3-Oxo-hexa-decanoyl-CoA
3-Oxo-tetra-decanoyl-CoA
3-Oxo-dodecanoyl-CoA
3-Oxo-decanoyl-CoA
3-Oxo-octanoyl-CoA
3-Oxo-hexanoyl-CoA
Acetoacetyl-CoA
2.3.1.16
2.3.1.9
CoA
Synthesis and degradation of ketone bodies
Long-chain-fatty acid
6.2.1.20
Long-chain acyl-[acyl-carrier protein]
[Acyl-carrier protein]
cis,cis-3,6-Dodecadienoyl-CoA
5.3.3.8
trans,cis-Lauro-2,6-dienoyl-CoA
(R)-3-Hydroxybutanoyl-CoA
5.1.2.3
(S)-3-Hydroxybutanoyl-CoA
Acetyl-CoA
Alanine and aspartate metabolism
Butanoate metabolism
Glyoxylate and dicarboxylate metabolism
Citrate cycle
Alkane
1.14.15.3
1.18.1.3
1.18.1.4
1.18.1.1
Rubredoxin (red)
Rubredoxin (ox)
1-Alcohol
1.1.1.1
1.1.99.20
Aldehyde
1.2.1.3
1.2.5.2
Fatty acid
1.14.14.80
1.14.14.1
ω-Hydroxy fatty acid
α-Hydroxy fatty acid
00071 5/25/20
(c) Kanehisa Laboratories

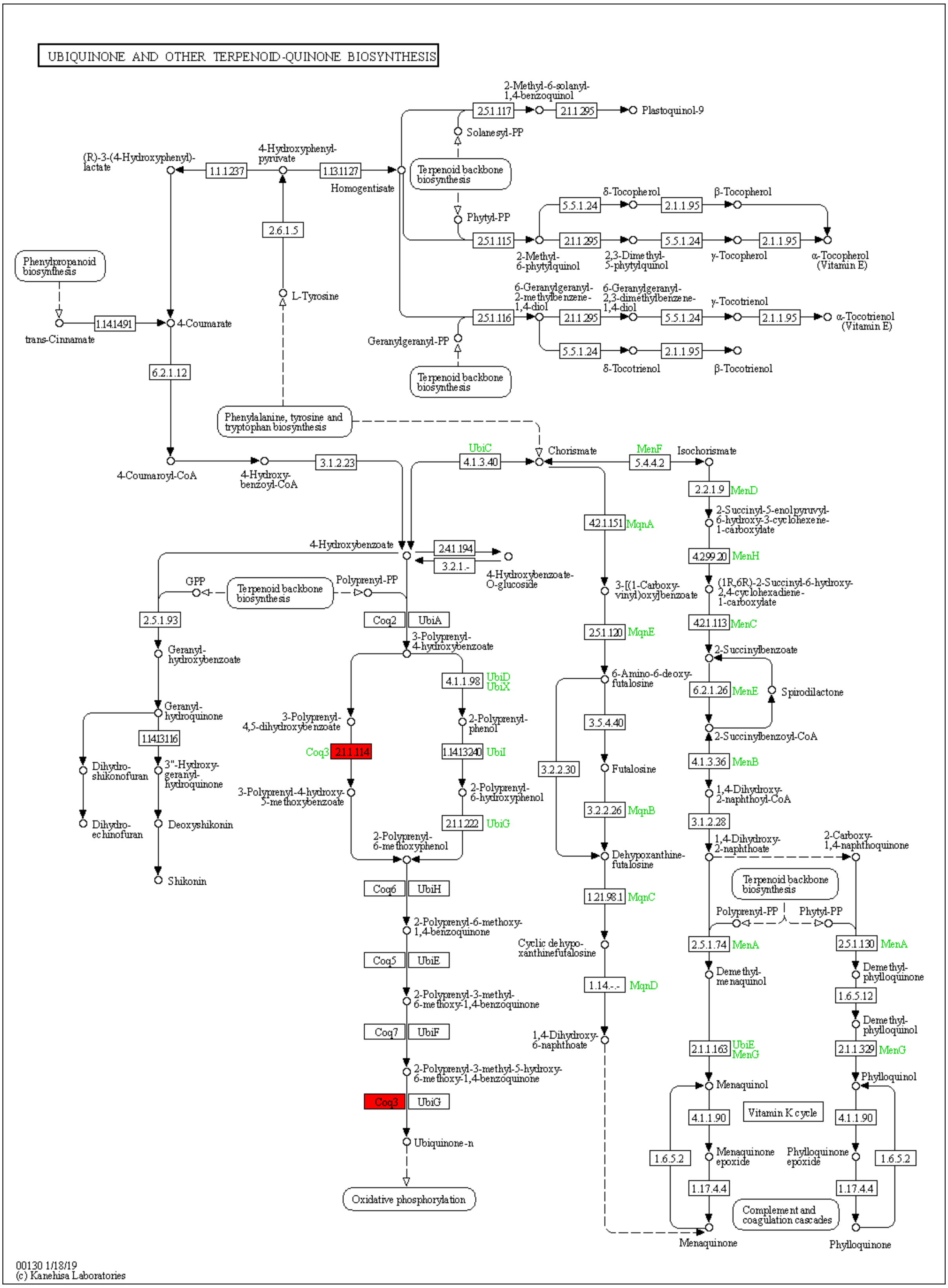
UBIQUINONE AND OTHER TERPENOID-QUINONE BIOSYNTHESIS
2-Methyl-6-solanyl-1,4-benzoquinol
2.5.1.117
2.1.1.295
Plastoquinol-9
Solanesyl-PP
(R)-3-(4-Hydroxyphenyl)-lactate
1.1.1.237
4-Hydroxyphenyl-pyruvate
1.13.11.27
Homogentisate
Terpenoid backbone biosynthesis
δ-Tocopherol
β-Tocopherol
5.5.1.24
2.1.1.95
Phytyl-PP
2.6.1.5
2.5.1.115
2.1.1.295
5.5.1.24
2.1.1.95
Phenylpropanoid biosynthesis
2-Methyl-6-phytylquinol
2,3-Dimethyl-5-phytylquinol
γ-Tocopherol
α-Tocopherol (Vitamin E)
L-Tyrosine
6-Geranylgeranyl-2-methylbenzene-1,4-diol
6-Geranylgeranyl-2,3-dimethylbenzene-1,4-diol
γ-Tocotrienol
1.14.14.91
4-Coumarate
2.5.1.116
2.1.1.295
5.5.1.24
2.1.1.95
α-Tocotrienol (Vitamin E)
trans-Cinnamate
Geranylgeranyl-PP
5.5.1.24
2.1.1.95
6.2.1.12
δ-Tocotrienol
β-Tocotrienol
Terpenoid backbone biosynthesis
Phenylalanine, tyrosine and tryptophan biosynthesis
UbiC
MenF
Chorismate
Isochorismate
4-Coumaroyl-CoA
4-Hydroxy-benzoyl-CoA
3.1.2.23
4.1.3.40
5.4.4.2
2.2.1.9
MenD
2-Succinyl-5-enolpyruvyl-6-hydroxy-3-cyclohexene-1-carboxylate
4.2.1.151
MqnA
4-Hydroxybenzoate
2.4.1.194
3.2.1.-
4-Hydroxybenzoate-O-glucoside
4.2.99.20
MenH
GPP
Terpenoid backbone biosynthesis
Polyprenyl-PP
3-[(1-Carboxy-vinyl)oxy]benzoate
(1R,6R)-2-Succinyl-6-hydroxy-2,4-cyclohexadiene-1-carboxylate
2.5.1.93
Coq2
UbiA
4.2.1.113
MenC
2.5.1.120
MqnE
3-Polyprenyl-4-hydroxybenzoate
Geranyl-hydroxybenzoate
2-Succinylbenzoate
6-Amino-6-deoxy-futalosine
4.1.1.98
UbiD
UbiX
6.2.1.26
MenE
Spirodilactone
Geranyl-hydroquinone
3-Polyprenyl-4,5-dihydroxybenzoate
2-Polyprenyl-phenol
3.5.4.40
2-Succinylbenzoyl-CoA
1.14.13.116
Coq3
2.1.1.114
1.14.13.240
UbiI
3.2.2.30
Futalosine
Dihydro-shikonofuran
3''-Hydroxy-geranyl-hydroquinone
4.1.3.36
MenB
3-Polyprenyl-4-hydroxy-5-methoxybenzoate
2-Polyprenyl-6-hydroxyphenol
1,4-Dihydroxy-2-naphthoyl-CoA
3.2.2.26
MqnB
2.1.1.222
UbiG
3.1.2.28
Dihydro-echinofuran
Deoxyshikonin
2-Polyprenyl-6-methoxyphenol
1,4-Dihydroxy-2-naphthoate
2-Carboxy-1,4-naphthoquinone
Dehypoxanthine-futalosine
Shikonin
Coq6
UbiH
Terpenoid backbone biosynthesis
1.21.98.1
MqnC
Polyprenyl-PP
Phytyl-PP
2-Polyprenyl-6-methoxy-1,4-benzoquinone
Cyclic dehypo-xanthinefutalosine
2.5.1.74
MenA
2.5.1.130
MenA
Coq5
UbiE
Demethyl-menaquinol
Demethyl-phylloquinone
1.14.-.-
MqnD
1.6.5.12
2-Polyprenyl-3-methyl-6-methoxy-1,4-benzoquinone
Demethyl-phylloquinol
Coq7
UbiF
1,4-Dihydroxy-6-naphthoate
2.1.1.163
UbiE
MenG
2.1.1.329
MenG
2-Polyprenyl-3-methyl-5-hydroxy-6-methoxy-1,4-benzoquinone
Menaquinol
Phylloquinol
Coq3
UbiG
Vitamin K cycle
4.1.1.90
4.1.1.90
Ubiquinone-n
1.6.5.2
Menaquinone epoxide
Phylloquinone epoxide
1.6.5.2
1.17.4.4
1.17.4.4
Oxidative phosphorylation
Complement and coagulation cascades
Menaquinone
Phylloquinone
00130 1/18/19
(c) Kanehisa Laboratories

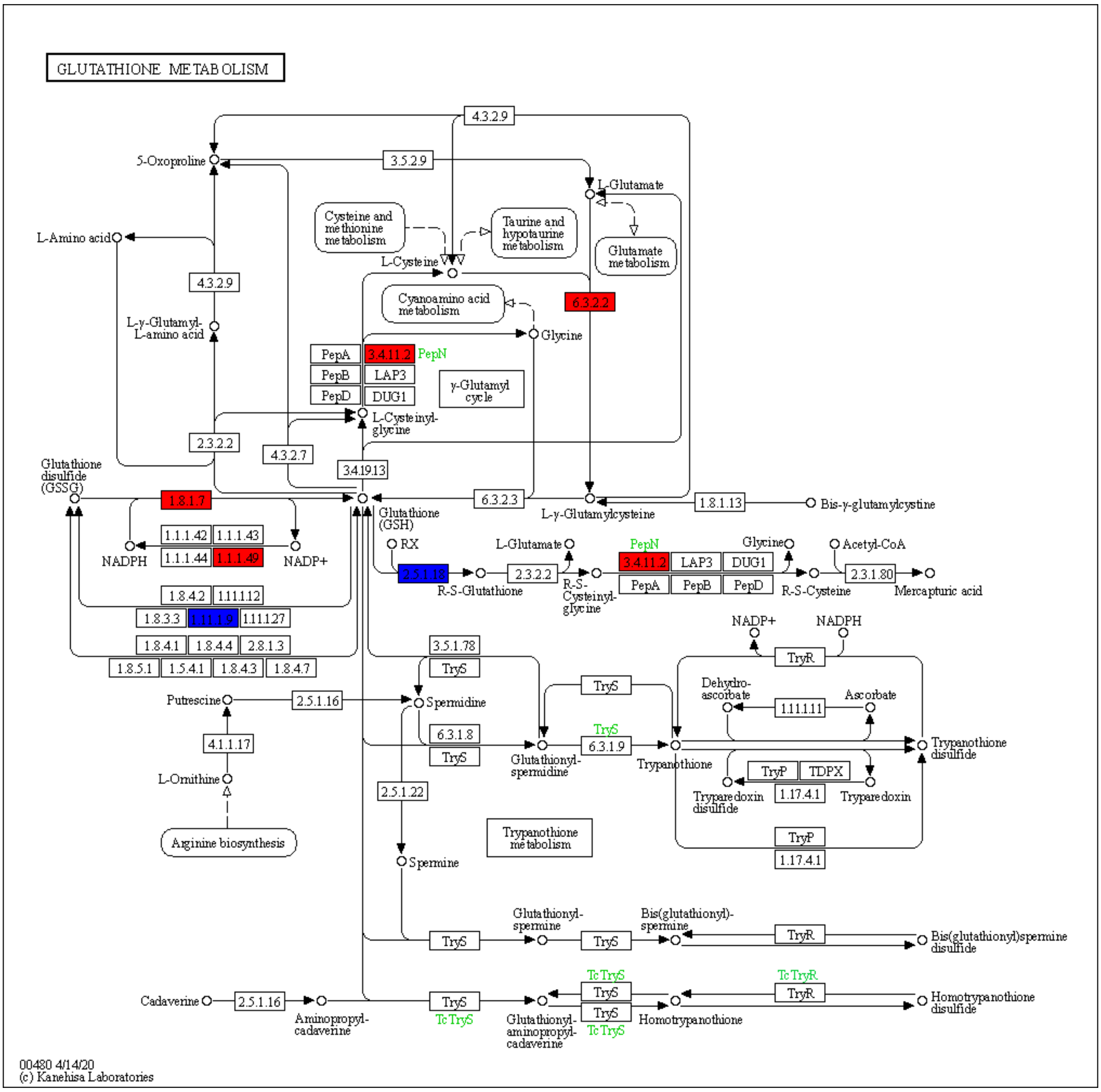
GLUTATHIONE METABOLISM
5-Oxoproline
L-Glutamate
L-Amino acid
L-Cysteine
L-γ-Glutamyl-L-amino acid
Glycine
L-Cysteinyl-glycine
Glutathione disulfide (GSSG)
Glutathione (GSH)
L-γ-Glutamylcysteine
Bis-γ-glutamylcystine
NADPH
NADP+
RX
R-S-Glutathione
R-S-Cysteinyl-glycine
R-S-Cysteine
Acetyl-CoA
Mercapturic acid
Cysteine and methionine metabolism
Taurine and hypotaurine metabolism
Glutamate metabolism
Cyanoamino acid metabolism
γ-Glutamyl cycle
Putrescine
Spermidine
Spermine
L-Ornithine
Arginine biosynthesis
Glutathionyl-spermidine
Trypanothione
Trypanothione disulfide
Dehydro-ascorbate
Ascorbate
Tryparedoxin disulfide
Tryparedoxin
Trypanothione metabolism
Glutathionyl-spermine
Bis(glutathionyl)-spermine
Bis(glutathionyl)spermine disulfide
Cadaverine
Aminopropyl-cadaverine
Glutathionyl-aminopropyl-cadaverine
Homotrypanothione
Homotrypanothione disulfide
4.3.2.9
3.5.2.9
6.3.2.2
PepA
PepB
PepD
3.4.11.2
LAP3
DUG1
PepN
2.3.2.2
4.3.2.7
3.4.19.13
6.3.2.3
1.8.1.13
1.8.1.7
1.1.1.42
1.1.1.43
1.1.1.44
1.1.1.49
2.5.1.18
2.3.1.80
1.8.4.2
1.11.1.12
1.8.3.3
1.11.1.9
1.11.1.27
1.8.4.1
1.8.4.4
2.8.1.3
1.8.5.1
1.5.4.1
1.8.4.3
1.8.4.7
3.5.1.78
TryS
2.5.1.16
4.1.1.17
6.3.1.8
6.3.1.9
2.5.1.22
TryR
1.11.1.11
TryP
TDPX
1.17.4.1
TcTryS
TcTryR
00480 4/14/20
(c) Kanehisa Laboratories

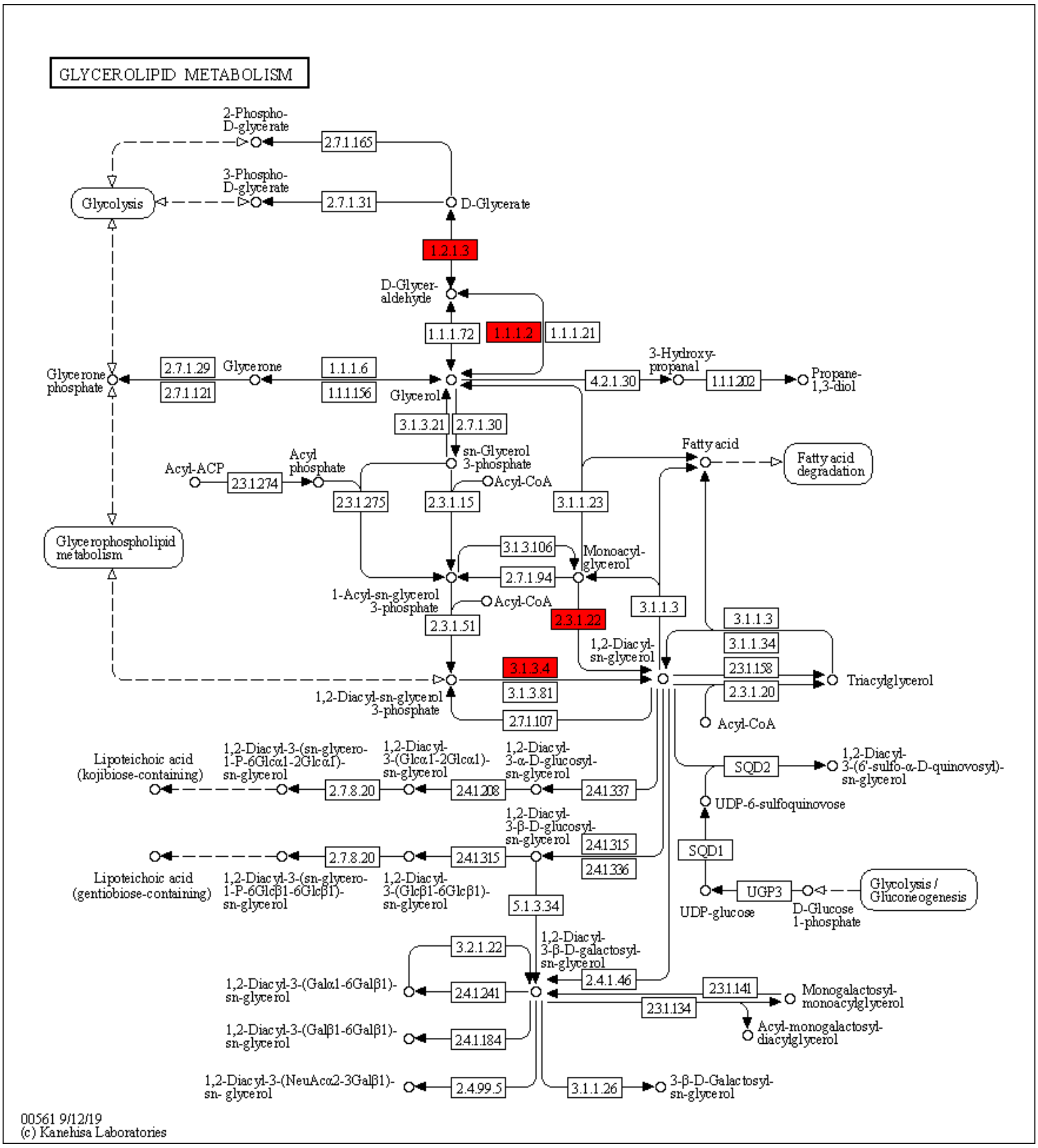
GLYCEROLIPID METABOLISM
2-Phospho-D-glycerate
2.7.1.165
3-Phospho-D-glycerate
2.7.1.31
Glycolysis
D-Glycerate
1.2.1.3
D-Glycer-aldehyde
1.1.1.72
1.1.1.2
1.1.1.21
Glycerone phosphate
2.7.1.29
2.7.1.121
Glycerone
1.1.1.6
1.1.1.156
Glycerol
4.2.1.30
3-Hydroxy-propanal
1.1.1.202
Propane-1,3-diol
3.1.3.21
2.7.1.30
sn-Glycerol 3-phosphate
Acyl-CoA
Acyl-ACP
2.3.1.274
Acyl phosphate
2.3.1.275
2.3.1.15
3.1.1.23
Fatty acid
Fatty acid degradation
Glycerophospholipid metabolism
3.1.3.106
Monoacyl-glycerol
2.7.1.94
1-Acyl-sn-glycerol 3-phosphate
Acyl-CoA
3.1.1.3
2.3.1.22
2.3.1.51
3.1.1.3
1,2-Diacyl-sn-glycerol
3.1.1.34
3.1.3.4
2.3.1.158
Triacylglycerol
3.1.3.81
2.3.1.20
1,2-Diacyl-sn-glycerol 3-phosphate
2.7.1.107
Acyl-CoA
Lipoteichoic acid (kojibiose-containing)
1,2-Diacyl-3-(sn-glycero-1-P-6Glcα1-2Glcα1)-sn-glycerol
1,2-Diacyl-3-(Glcα1-2Glcα1)-sn-glycerol
1,2-Diacyl-3-α-D-glucosyl-sn-glycerol
2.7.8.20
2.4.1.208
2.4.1.337
SQD2
1,2-Diacyl-3-(6'-sulfo-α-D-quinovosyl)-sn-glycerol
UDP-6-sulfoquinovose
1,2-Diacyl-3-β-D-glucosyl-sn-glycerol
2.4.1.315
2.4.1.336
2.7.8.20
2.4.1.315
SQD1
Lipoteichoic acid (gentiobiose-containing)
1,2-Diacyl-3-(sn-glycero-1-P-6Glcβ1-6Glcβ1)-sn-glycerol
1,2-Diacyl-3-(Glcβ1-6Glcβ1)-sn-glycerol
5.1.3.34
UGP3
Glycolysis / Gluconeogenesis
UDP-glucose
D-Glucose 1-phosphate
1,2-Diacyl-3-β-D-galactosyl-sn-glycerol
3.2.1.22
2.4.1.46
1,2-Diacyl-3-(Galα1-6Galβ1)-sn-glycerol
2.4.1.241
2.3.1.141
Monogalactosyl-monoacylglycerol
2.3.1.134
1,2-Diacyl-3-(Galβ1-6Galβ1)-sn-glycerol
2.4.1.184
Acyl-monogalactosyl-diacylglycerol
1,2-Diacyl-3-(NeuAcα2-3Galβ1)-sn- glycerol
2.4.99.5
3.1.1.26
3-β-D-Galactosyl-sn-glycerol
00561 9/12/19
(c) Kanehisa Laboratories

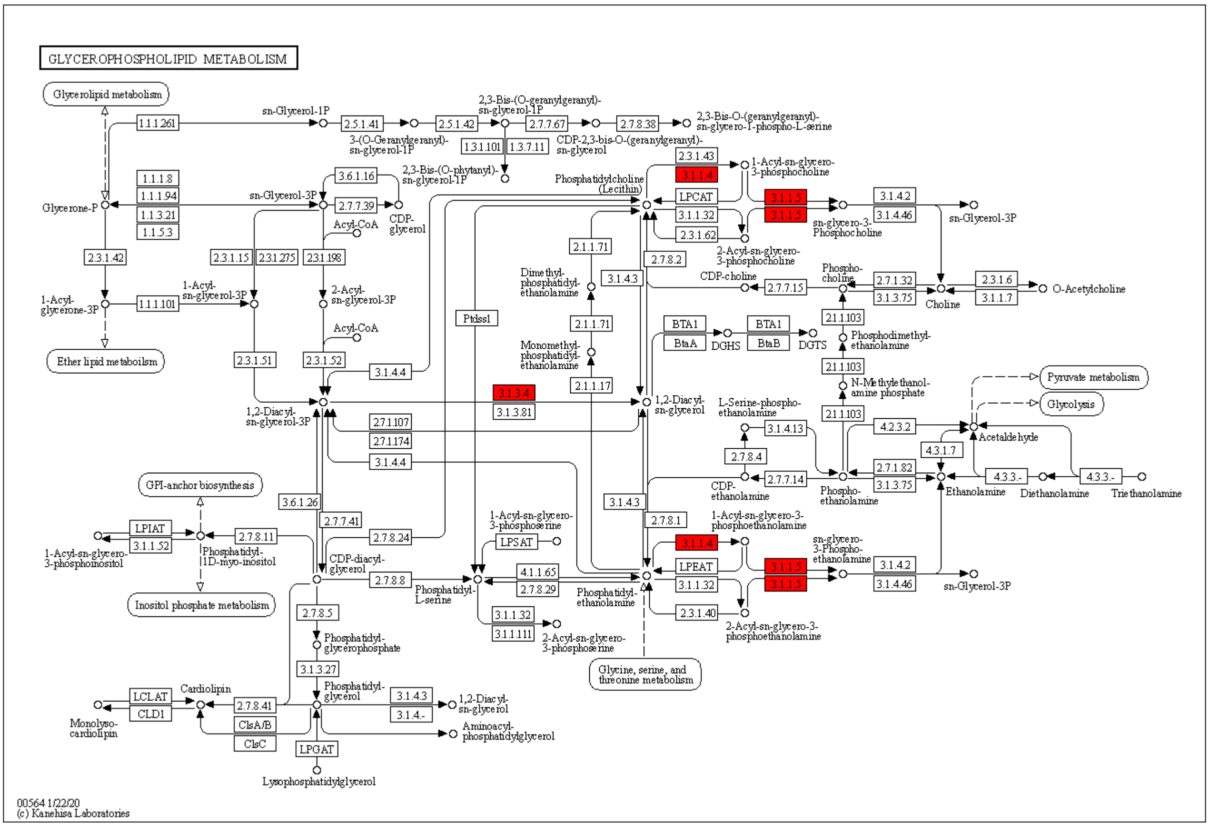
GLYCEROPHOSPHOLIPID METABOLISM
Glycerolipid metabolism
Ether lipid metaboilsm
GPI-anchor biosynthesis
Inositol phosphate metabolism
Glycine, serine, and threonine metabolism
Pyruvate metabolism
Glycolysis
Glycerone-P
1-Acyl-glycerone-3P
sn-Glycerol-1P
sn-Glycerol-3P
3-(O-Geranylgeranyl)-sn-glycerol-1P
2,3-Bis-(O-geranylgeranyl)-sn-glycerol-1P
2,3-Bis-(O-phytanyl)-sn-glycerol-1P
CDP-2,3-bis-O-(geranylgeranyl)-sn-glycerol
2,3-Bis-O-(geranylgeranyl)-sn-glycero-1-phospho-L-serine
CDP-glycerol
Acyl-CoA
1-Acyl-sn-glycerol-3P
2-Acyl-sn-glycerol-3P
1,2-Diacyl-sn-glycerol-3P
CDP-diacyl-glycerol
Phosphatidyl-glycerophosphate
Phosphatidyl-glycerol
Cardiolipin
Monolyso-cardiolipin
Lysophosphatidylglycerol
1,2-Diacyl-sn-glycerol
Aminoacyl-phosphatidylglycerol
1-Acyl-sn-glycero-3-phosphoinositol
Phosphatidyl-1D-myo-inositol
Phosphatidyl-L-serine
1-Acyl-sn-glycero-3-phosphoserine
2-Acyl-sn-glycero-3-phosphoserine
Phosphatidyl-ethanolamine
1-Acyl-sn-glycero-3-phosphoethanolamine
2-Acyl-sn-glycero-3-phosphoethanolamine
sn-glycero-3-Phospho-ethanolamine
Phosphatidylcholine (Lecithin)
1-Acyl-sn-glycero-3-phosphocholine
2-Acyl-sn-glycero-3-phosphocholine
sn-glycero-3-Phosphocholine
Dimethyl-phosphatidyl-ethanolamine
Monomethyl-phosphatidyl-ethanolamine
CDP-choline
Phospho-choline
Choline
O-Acetylcholine
DGHS
DGTS
Phosphodimethyl-ethanolamine
N-Methylethanol-amine phosphate
L-Serine-phospho-ethanolamine
CDP-ethanolamine
Phospho-ethanolamine
Ethanolamine
Diethanolamine
Triethanolamine
Acetaldehyde
1.1.1.261
1.1.1.8
1.1.1.94
1.1.3.21
1.1.5.3
2.3.1.42
1.1.1.101
2.5.1.41
2.5.1.42
2.7.7.67
2.7.8.38
1.3.1.101
1.3.7.11
3.6.1.16
2.7.7.39
2.3.1.15
2.3.1.275
2.3.1.198
2.3.1.51
2.3.1.52
3.1.4.4
3.1.3.4
3.1.3.81
2.7.1.107
2.7.1.174
3.6.1.26
2.7.7.41
2.7.8.24
2.7.8.11
LPIAT
3.1.1.52
2.7.8.8
2.7.8.5
3.1.3.27
3.1.4.3
3.1.4.-
2.7.8.41
LCLAT
CLD1
ClsA/B
ClsC
LPGAT
Ptdss1
LPSAT
4.1.1.65
2.7.8.29
3.1.1.32
3.1.1.111
2.3.1.43
3.1.1.4
LPCAT
3.1.1.62
3.1.1.5
3.1.4.2
3.1.4.46
sn-Glycerol-3P
2.1.1.71
2.1.1.17
2.7.8.2
2.7.7.15
2.7.1.32
3.1.3.75
2.3.1.6
3.1.1.7
BTA1
BtaA
BtaB
2.1.1.103
3.1.4.13
2.7.8.4
2.7.7.14
2.7.1.82
4.2.3.2
4.3.1.7
4.3.3.-
2.7.8.1
LPEAT
2.3.1.40
00564 1/22/20
(c) Kanehisa Laboratories

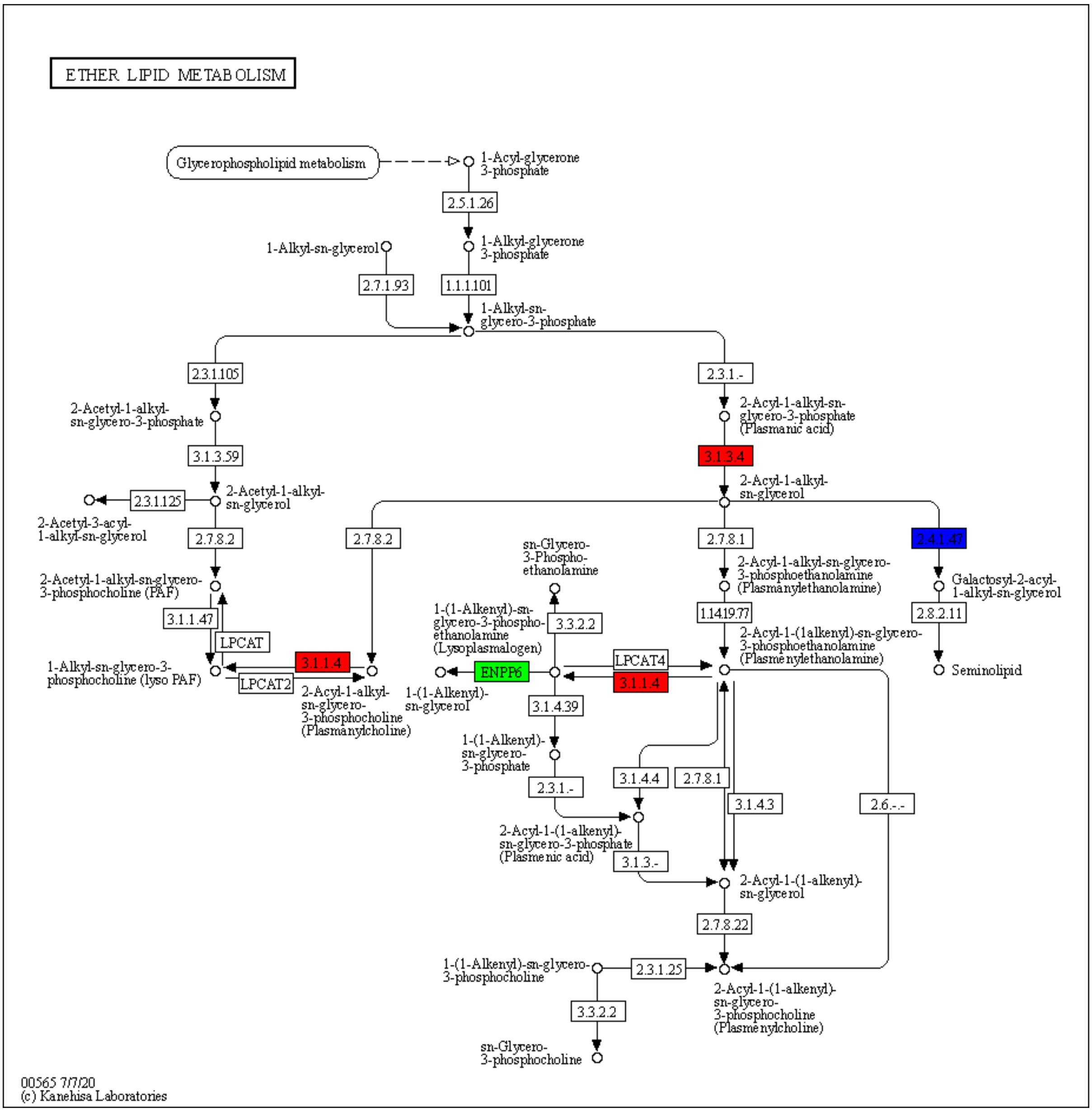
ETHER LIPID METABOLISM
Glycerophospholipid metabolism
1-Acyl-glycerone 3-phosphate
2.5.1.26
1-Alkyl-glycerone 3-phosphate
1-Alkyl-sn-glycerol
2.7.1.93
1.1.1.101
1-Alkyl-sn-glycero-3-phosphate
2.3.1.105
2.3.1.-
2-Acetyl-1-alkyl-sn-glycero-3-phosphate
2-Acyl-1-alkyl-sn-glycero-3-phosphate (Plasmanic acid)
3.1.3.59
3.1.3.4
2.3.1.125
2-Acetyl-1-alkyl-sn-glycerol
2-Acyl-1-alkyl-sn-glycerol
2-Acetyl-3-acyl-1-alkyl-sn-glycerol
2.7.8.2
2.7.8.2
2.7.8.1
2.4.1.47
sn-Glycero-3-Phospho-ethanolamine
2-Acetyl-1-alkyl-sn-glycero-3-phosphocholine (PAF)
2-Acyl-1-alkyl-sn-glycero-3-phosphoethanolamine (Plasmanylethanolamine)
Galactosyl-2-acyl-1-alkyl-sn-glycerol
1-(1-Alkenyl)-sn-glycero-3-phospho-ethanolamine (Lysoplasmalogen)
3.3.2.2
1.14.19.77
2.8.2.11
3.1.1.47
LPCAT
LPCAT4
2-Acyl-1-(1alkenyl)-sn-glycero-3-phosphoethanolamine (Plasmenylethanolamine)
1-Alkyl-sn-glycero-3-phosphocholine (lyso PAF)
3.1.1.4
ENPP6
3.1.1.4
Seminolipid
LPCAT2
2-Acyl-1-alkyl-sn-glycero-3-phosphocholine (Plasmanylcholine)
1-(1-Alkenyl)-sn-glycerol
3.1.4.39
1-(1-Alkenyl)-sn-glycero-3-phosphate
3.1.4.4
2.7.8.1
3.1.4.3
2.6.-.-
2.3.1.-
2-Acyl-1-(1-alkenyl)-sn-glycero-3-phosphate (Plasmenic acid)
3.1.3.-
2-Acyl-1-(1-alkenyl)-sn-glycerol
2.7.8.22
1-(1-Alkenyl)-sn-glycero-3-phosphocholine
2.3.1.25
2-Acyl-1-(1-alkenyl)-sn-glycero-3-phosphocholine (Plasmenylcholine)
3.3.2.2
sn-Glycero-3-phosphocholine
00565 7/7/20
(c) Kanehisa Laboratories

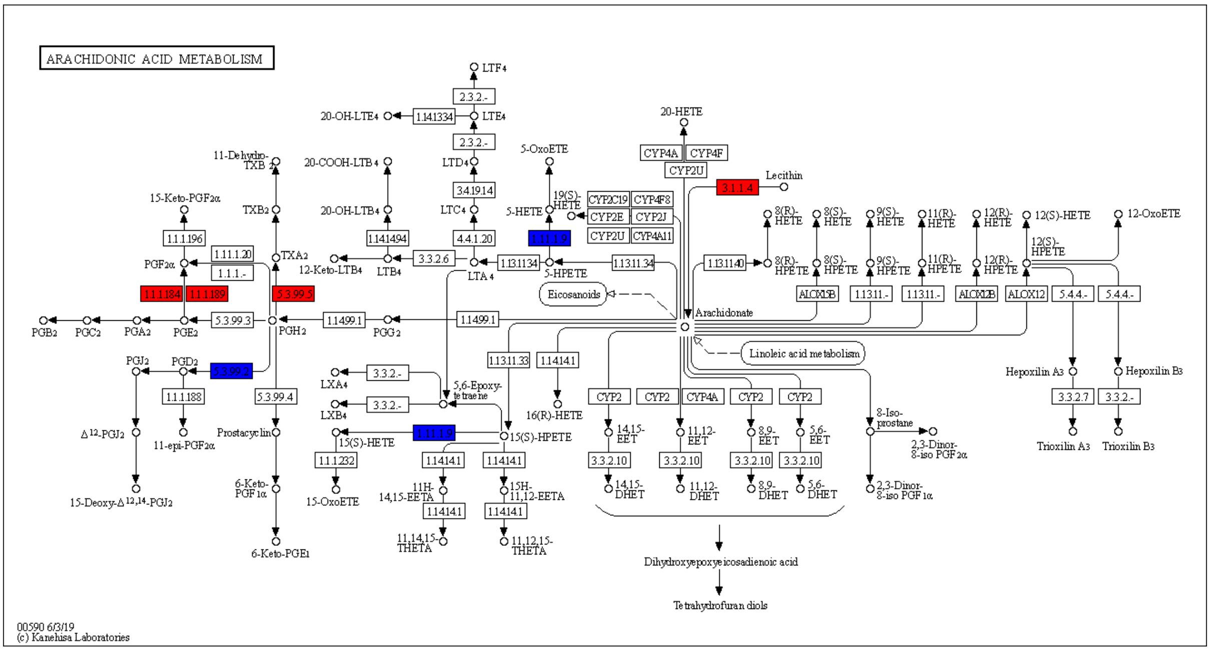
ARACHIDONIC ACID METABOLISM
LTF4
2.3.2.-
20-OH-LTE4
1.14.13.34
LTE4
2.3.2.-
20-HETE
CYP4A
CYP4F
CYP2U
11-Dehydro-TXB2
20-COOH-LTB4
LTD4
5-OxoETE
3.4.19.14
3.1.1.4
Lecithin
15-Keto-PGF2α
TXB2
20-OH-LTB4
LTC4
5-HETE
19(S)-HETE
CYP2C19
CYP4F8
CYP2E
CYP2J
CYP2U
CYP4A11
12(S)-HETE
12-OxoETE
1.1.1.196
1.14.14.94
4.4.1.20
PGF2α
1.1.1.20
1.1.1.-
TXA2
12-Keto-LTB4
3.3.2.6
LTB4
LTA4
1.13.11.34
5-HPETE
1.13.11.34
1.13.11.40
8(R)-HPETE
8(S)-HPETE
9(S)-HPETE
11(R)-HPETE
12(R)-HPETE
12(S)-HPETE
1.1.1.184
1.1.1.189
5.3.99.5
Eicosanoids
ALOX15B
1.13.11.-
1.13.11.-
ALOX12B
ALOX12
5.4.4.-
5.4.4.-
PGB2
PGC2
PGA2
PGE2
5.3.99.3
PGH2
1.14.99.1
PGG2
1.14.99.1
Arachidonate
Linoleic acid metabolism
PGJ2
PGD2
5.3.99.2
1.13.11.33
1.14.14.1
Hepoxilin A3
Hepoxilin B3
LXA4
3.3.2.-
5,6-Epoxy-tetraene
1.1.1.188
5.3.99.4
3.3.2.-
16(R)-HETE
CYP2
CYP2
CYP4A
CYP2
CYP2
3.3.2.7
3.3.2.-
8-Iso-prostane
LXB4
Δ12-PGJ2
11-epi-PGF2α
Prostacyclin
1.11.1.9
15(S)-HETE
15(S)-HPETE
14,15-EET
11,12-EET
8,9-EET
5,6-EET
2,3-Dinor-8-iso PGF2α
Trioxilin A3
Trioxilin B3
1.1.1.232
1.14.14.1
1.14.14.1
3.3.2.10
3.3.2.10
3.3.2.10
3.3.2.10
6-Keto-PGF1α
15-OxoETE
11H-14,15-EETA
15H-11,12-EETA
14,15-DHET
11,12-DHET
8,9-DHET
5,6-DHET
2,3-Dinor-8-iso PGF1α
15-Deoxy-Δ12,14-PGJ2
1.14.14.1
1.14.14.1
11,14,15-THETA
11,12,15-THETA
6-Keto-PGE1
Dihydroxyepoxyeicosadienoic acid
Tetrahydrofuran diols
00590 6/3/19
(c) Kanehisa Laboratories

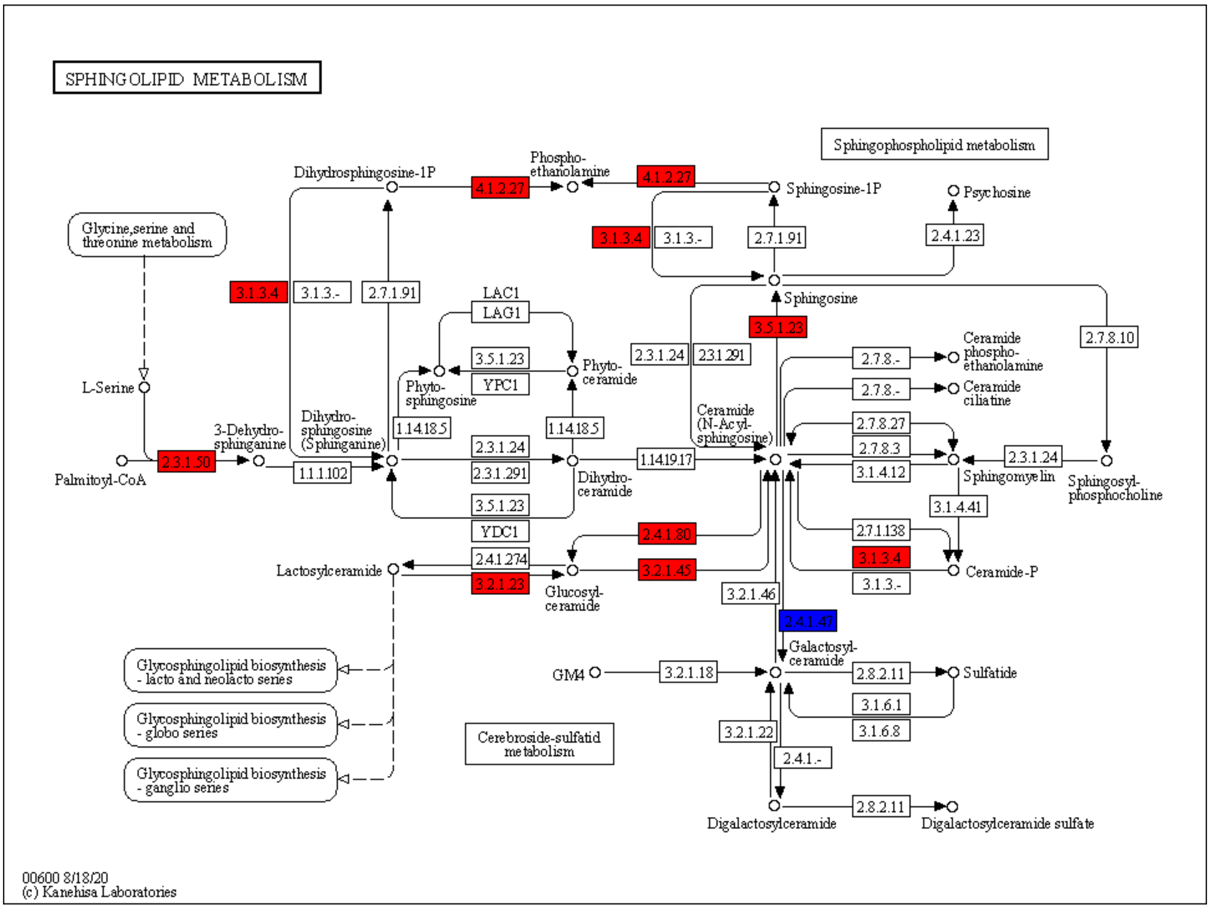
SPHINGOLIPID METABOLISM
Sphingophospholipid metabolism
Phospho-ethanolamine
Dihydrosphingosine-1P
4.1.2.27
4.1.2.27
Sphingosine-1P
Psychosine
Glycine, serine and threonine metabolism
3.1.3.4
3.1.3.-
2.7.1.91
2.4.1.23
3.1.3.4
3.1.3.-
2.7.1.91
LAC1
LAG1
Sphingosine
3.5.1.23
2.7.8.10
3.5.1.23
YPC1
Phyto-ceramide
2.3.1.24
2.3.1.291
2.7.8.-
Ceramide phospho-ethanolamine
L-Serine
Phyto-sphingosine
2.7.8.-
Ceramide ciliatine
3-Dehydro-sphinganine
Dihydro-sphingosine (Sphinganine)
1.14.18.5
1.14.18.5
Ceramide (N-Acyl-sphingosine)
2.7.8.27
2.7.8.3
Palmitoyl-CoA
2.3.1.50
1.1.1.102
2.3.1.24
2.3.1.291
1.14.19.17
3.1.4.12
2.3.1.24
Sphingomyelin
Sphingosyl-phosphocholine
3.5.1.23
Dihydro-ceramide
3.1.4.41
YDC1
2.4.1.80
2.7.1.138
Lactosylceramide
2.4.1.274
3.2.1.45
3.1.3.4
Ceramide-P
3.2.1.23
Glucosyl-ceramide
3.2.1.46
3.1.3.-
2.4.1.47
Galactosyl-ceramide
Glycosphingolipid biosynthesis - lacto and neolacto series
GM4
3.2.1.18
2.8.2.11
Sulfatide
3.1.6.1
Glycosphingolipid biosynthesis - globo series
3.1.6.8
Cerebroside-sulfatid metabolism
3.2.1.22
2.4.1.-
Glycosphingolipid biosynthesis - ganglio series
Digalactosylceramide
2.8.2.11
Digalactosylceramide sulfate
00600 8/18/20
(c) Kanehisa Laboratories

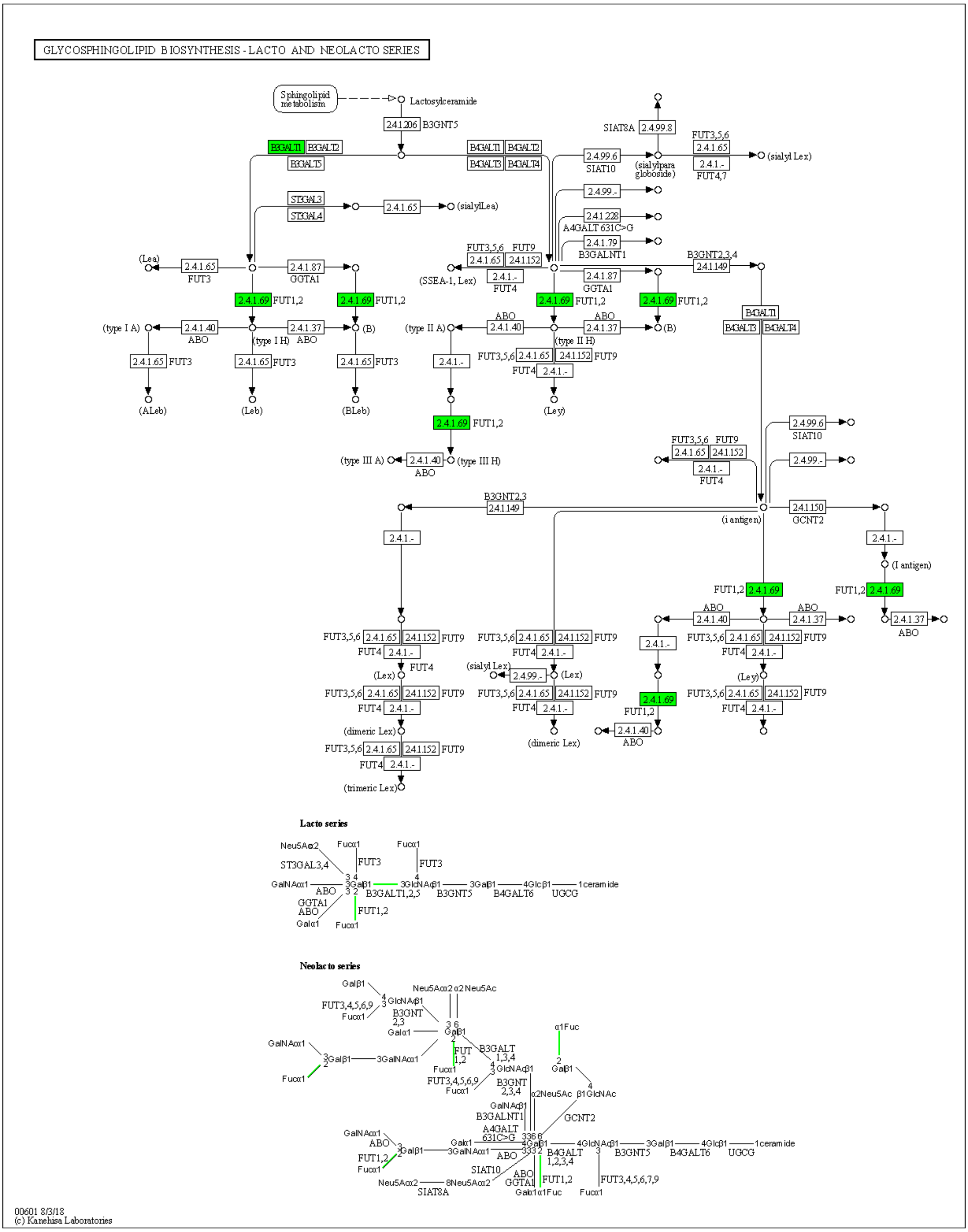
GLYCOSPHINGOLIPID BIOSYNTHESIS - LACTO AND NEOLACTO SERIES
Sphingolipid metabolism
Lactosylceramide
2.4.1.206 B3GNT5
B3GALT1 B3GALT2
B3GALT5
B4GALT1 B4GALT2
B4GALT3 B4GALT4
ST3GAL3
ST3GAL4
2.4.1.65
(sialylLea)
(Lea)
2.4.1.65 FUT3
2.4.1.87 GGTA1
2.4.1.69 FUT1,2
(type I A)
2.4.1.40 ABO
2.4.1.37 ABO
(type I H)
(B)
(ALeb)
(Leb)
(BLeb)
SIAT8A 2.4.99.8
2.4.99.6 SIAT10
(sialylparagloboside)
FUT3,5,6 2.4.1.65
2.4.1.- FUT4,7
(sialyl Lex)
2.4.99.-
2.4.1.228 A4GALT 631C>G
2.4.1.79 B3GALNT1
FUT3,5,6 FUT9
2.4.1.65 2.4.1.152
2.4.1.- FUT4
(SSEA-1, Lex)
B3GNT2,3,4
2.4.1.149
(type II A)
(type II H)
(Ley)
B4GALT1
B4GALT3 B4GALT4
2.4.1.-
(type III A)
(type III H)
SIAT10
(i antigen)
2.4.1.150 GCNT2
B3GNT2,3
(I antigen)
(Lex)
(dimeric Lex)
(trimeric Lex)
(sialyl Lex)
Lacto series
Neu5Acα2
Fucα1
ST3GAL3,4
FUT3
GalNAcα1
3Galβ1
3GlcNAcβ1
3Galβ1
4Glcβ1
1ceramide
ABO
B3GALT1,2,5
B3GNT5
B4GALT6
UGCG
GGTA1
Galα1
FUT1,2
Neolacto series
Galβ1
Neu5Acα2 α2 Neu5Ac
FUT3,4,5,6,9
B3GNT 2,3
GlcNAcβ1
Galα1
GalNAcα1
3GalNAcα1
B3GALT 1,3,4
FUT 1,2
α1Fuc
GCNT2
β1GlcNAc
α2Neu5Ac
B3GNT 2,3,4
GalNAcβ1
B3GALNT1
A4GALT 631C>G
4GlcNAcβ1
B4GALT 1,2,3,4
SIAT10
8Neu5Acα2
SIAT8A
Galα1α1Fuc
FUT3,4,5,6,7,9
00601 8/3/18
(c) Kanehisa Laboratories

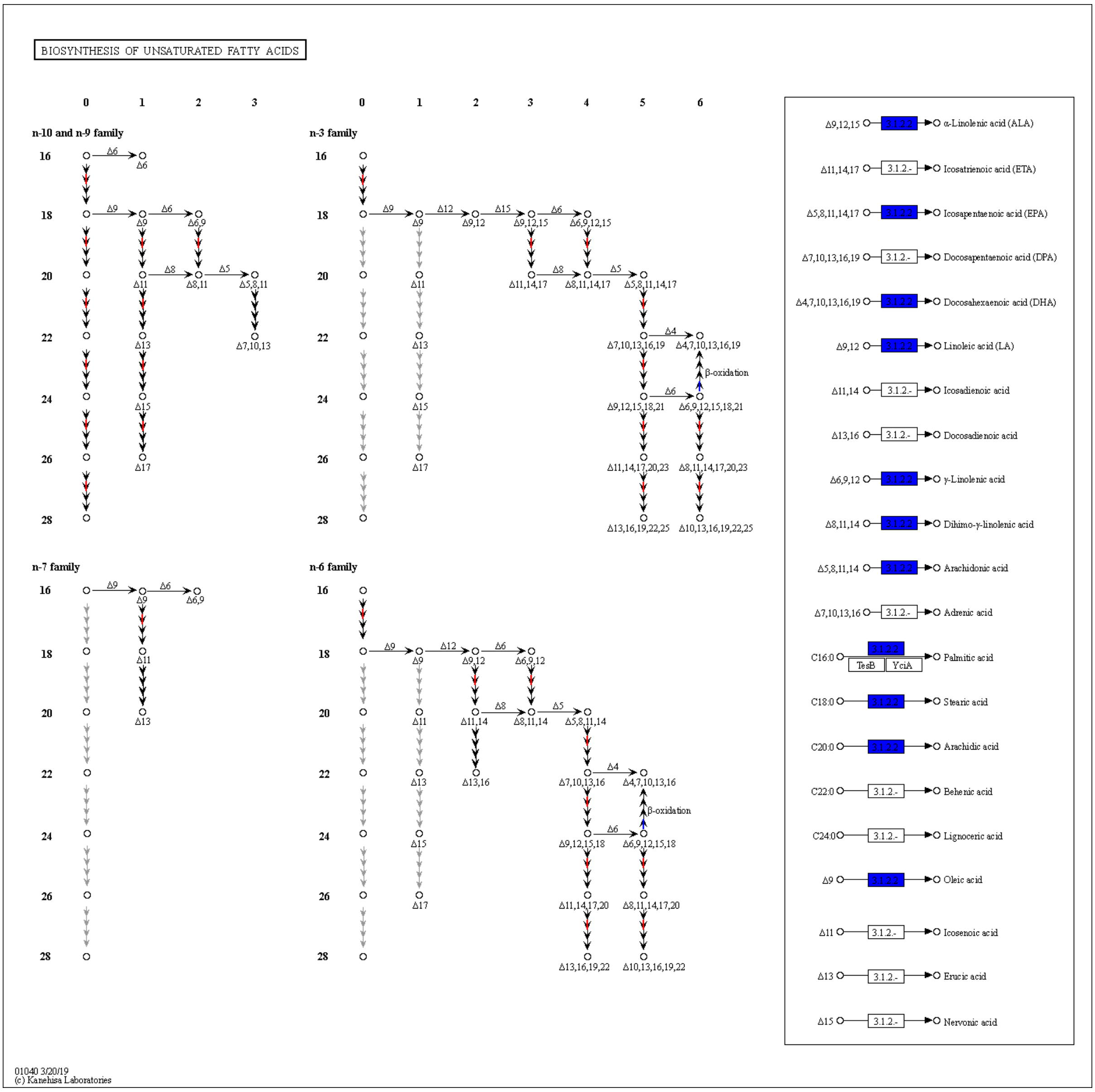
BIOSYNTHESIS OF UNSATURATED FATTY ACIDS
0 1 2 3
0 1 2 3 4 5 6
n-10 and n-9 family
16 18 20 22 24 26 28
Δ6
Δ9 Δ6 Δ6,9
Δ11 Δ8 Δ8,11 Δ5 Δ5,8,11
Δ13 Δ7,10,13
Δ15
Δ17
n-3 family
Δ9 Δ12 Δ9,12 Δ15 Δ9,12,15 Δ6 Δ6,9,12,15
Δ11,14,17 Δ8 Δ8,11,14,17 Δ5 Δ5,8,11,14,17
Δ7,10,13,16,19 Δ4 Δ4,7,10,13,16,19
β-oxidation
Δ9,12,15,18,21 Δ6 Δ6,9,12,15,18,21
Δ11,14,17,20,23 Δ8,11,14,17,20,23
Δ13,16,19,22,25 Δ10,13,16,19,22,25
n-7 family
Δ9 Δ6 Δ6,9
Δ11
Δ13
n-6 family
Δ9 Δ12 Δ9,12 Δ6 Δ6,9,12
Δ11 Δ11,14 Δ8 Δ8,11,14 Δ5 Δ5,8,11,14
Δ13 Δ13,16 Δ7,10,13,16 Δ4 Δ4,7,10,13,16
β-oxidation
Δ15 Δ9,12,15,18 Δ6 Δ6,9,12,15,18
Δ17 Δ11,14,17,20 Δ8,11,14,17,20
Δ13,16,19,22 Δ10,13,16,19,22
Δ9,12,15 3.1.2.- α-Linolenic acid (ALA)
Δ11,14,17 3.1.2.- Icosatrienoic acid (ETA)
Δ5,8,11,14,17 3.1.2.- Icosapentaenoic acid (EPA)
Δ7,10,13,16,19 3.1.2.- Docosapentaenoic acid (DPA)
Δ4,7,10,13,16,19 3.1.2.- Docosahexaenoic acid (DHA)
Δ9,12 3.1.2.- Linoleic acid (LA)
Δ11,14 3.1.2.- Icosadienoic acid
Δ13,16 3.1.2.- Docosadienoic acid
Δ6,9,12 3.1.2.- γ-Linolenic acid
Δ8,11,14 3.1.2.- Dihimo-γ-linolenic acid
Δ5,8,11,14 3.1.2.- Arachidonic acid
Δ7,10,13,16 3.1.2.- Adrenic acid
C16:0 3.1.2.- TesB YciA Palmitic acid
C18:0 3.1.2.- Stearic acid
C20:0 3.1.2.- Arachidic acid
C22:0 3.1.2.- Behenic acid
C24:0 3.1.2.- Lignoceric acid
Δ9 3.1.2.- Oleic acid
Δ11 3.1.2.- Icosenoic acid
Δ13 3.1.2.- Erucic acid
Δ15 3.1.2.- Nervonic acid
01040 3/20/19
(c) Kanehisa Laboratories

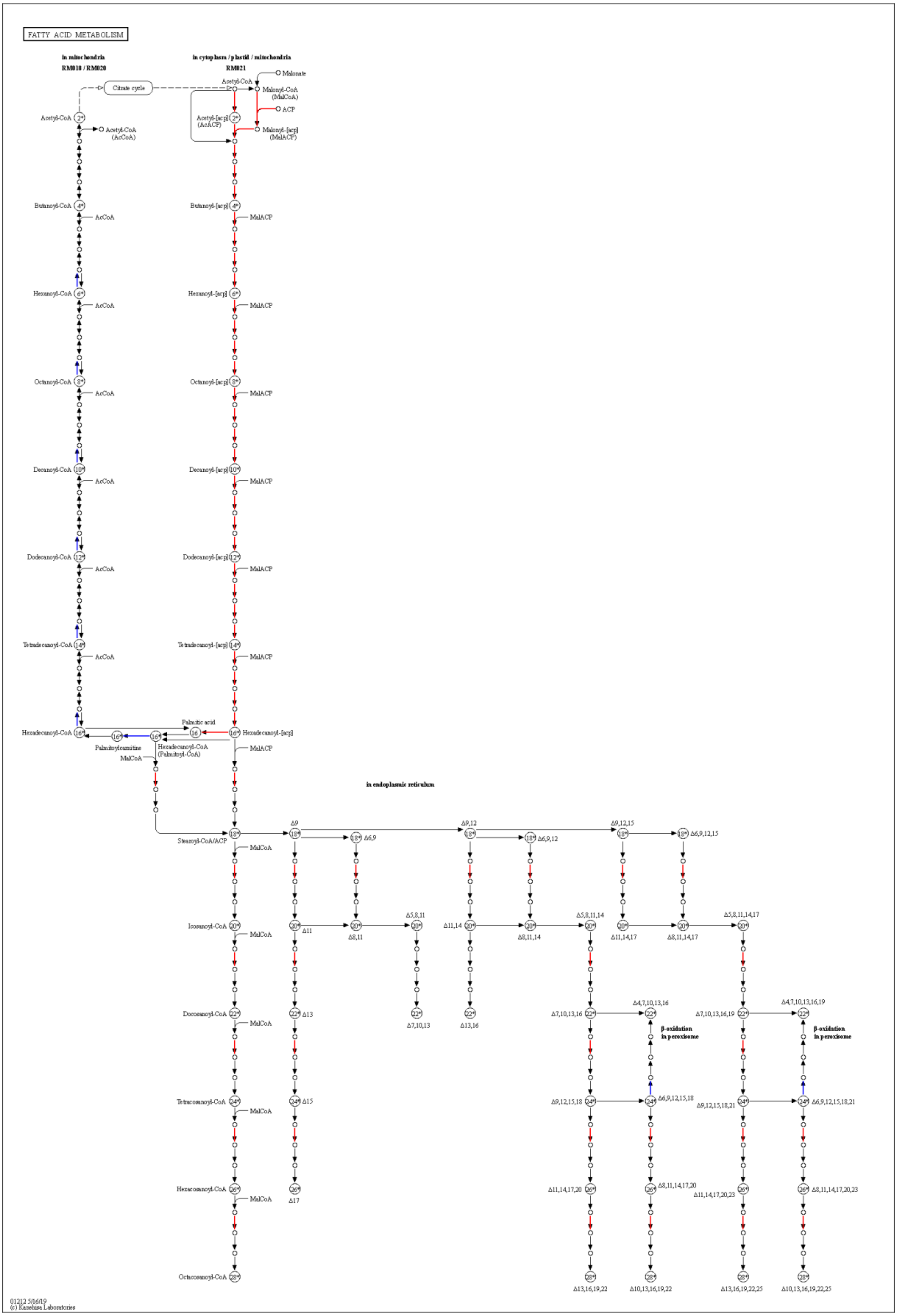

Supplementary Figure S8. Enriched KEGG pathways of chalcone-exposed worms compared to controls. Red: indicates pathways with predominantly upregulated genes, green: indicates predominantly downregulated genes, blue: indicates pathways containing both upregulated and underregulated genes.

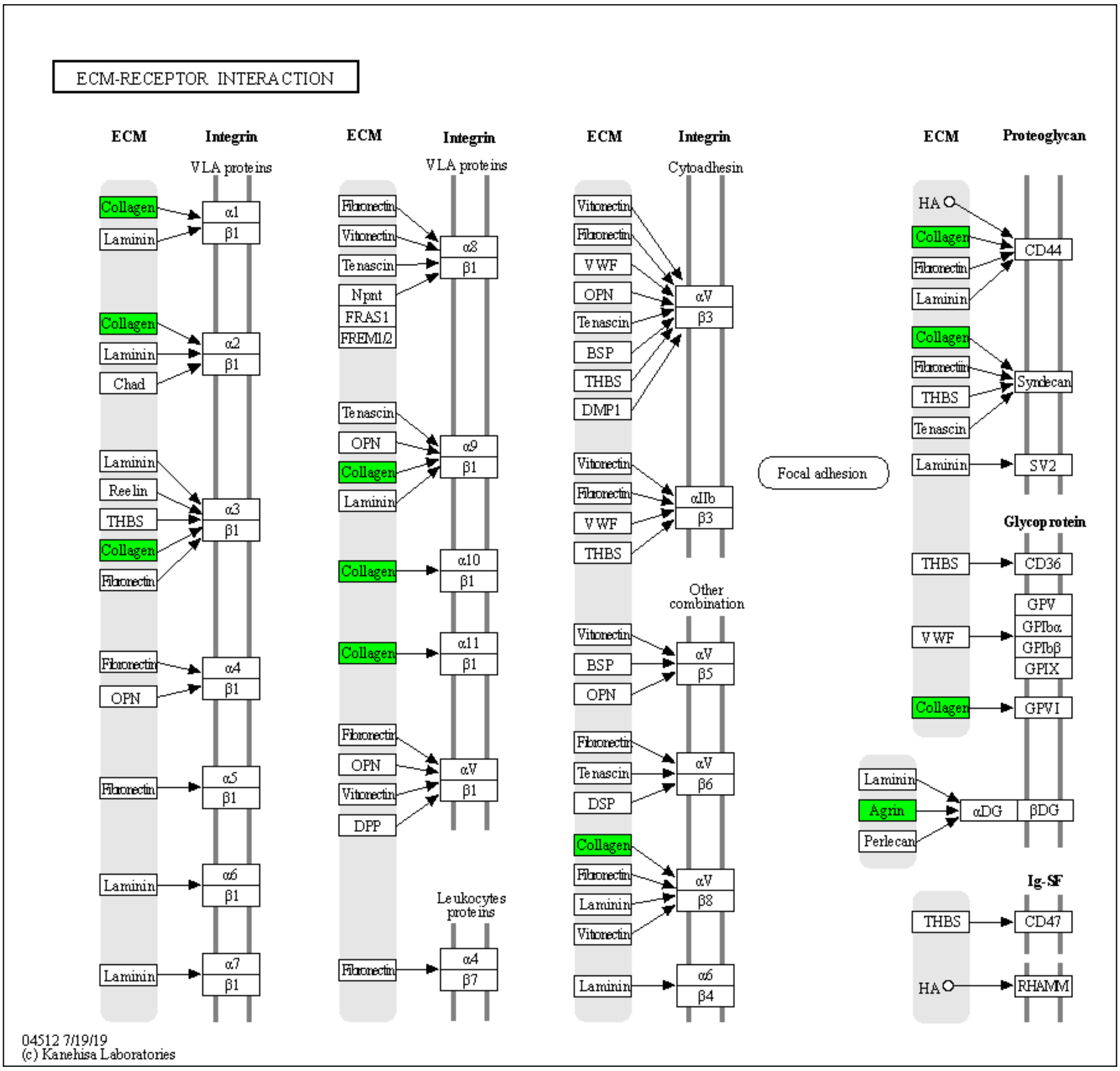
ECM-RECEPTOR INTERACTION
ECM
Integrin
VLA proteins
Cytoadhesin
Proteoglycan
Glycoprotein
Ig-SF
Other combination
Leukocytes proteins
Focal adhesion
Collagen
Laminin
Chad
Reelin
THBS
Fibronectin
OPN
Vitronectin
Tenascin
Npnt
FRAS1
FREM1/2
DPP
VWF
BSP
DMP1
DSP
HA
Agrin
Perlecan
CD44
Syndecan
SV2
CD36
GPV
GPIbα
GPIbβ
GPIX
GPVI
αDG
βDG
CD47
RHAMM
α1
α2
α3
α4
α5
α6
α7
α8
α9
α10
α11
αV
αIIb
β1
β3
β4
β5
β6
β7
β8
04512 7/19/19
(c) Kanehisa Laboratories

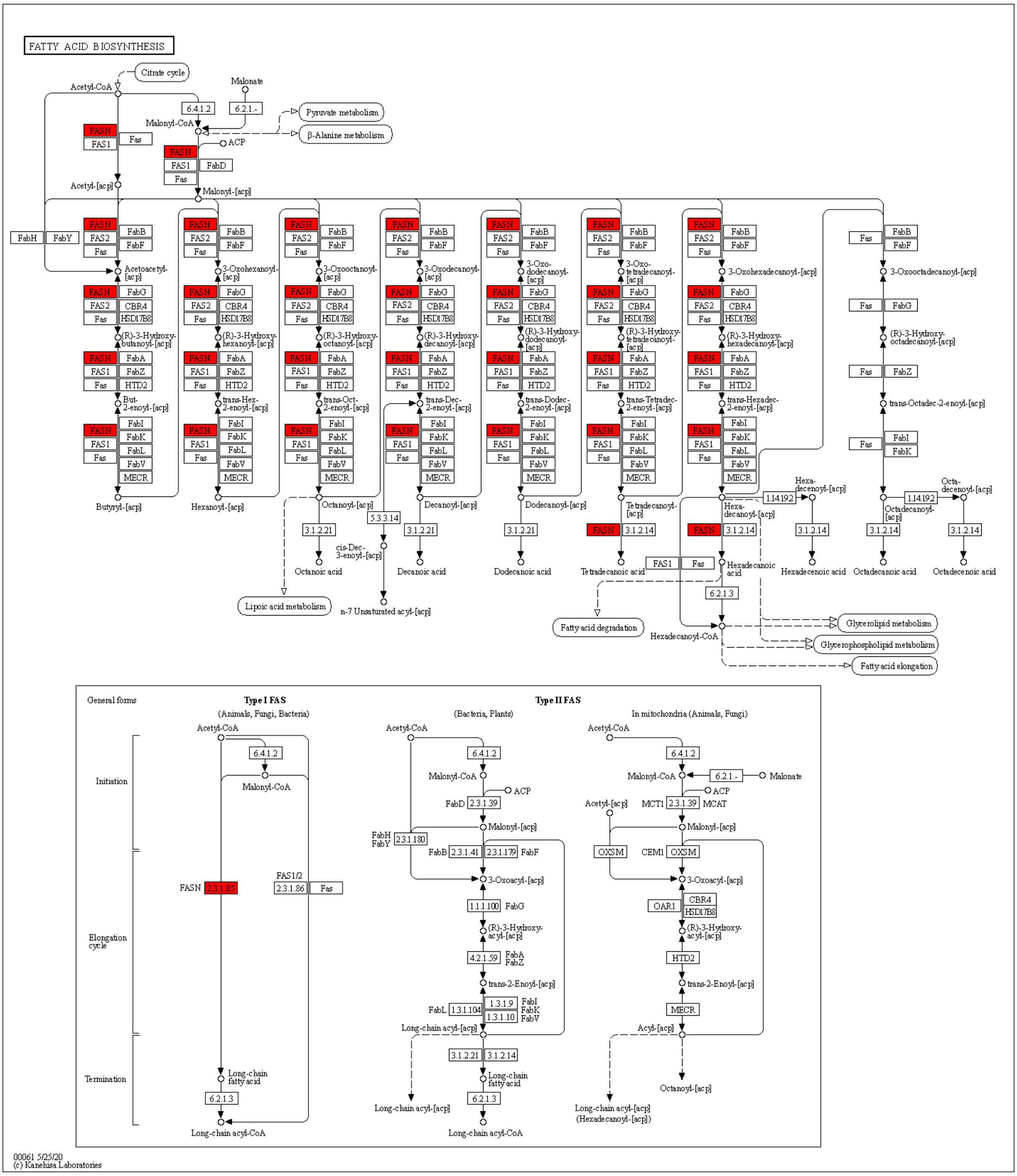
FATTY ACID BIOSYNTHESIS
Citrate cycle
Acetyl-CoA
Malonate
6.4.1.2
6.2.1.-
Pyruvate metabolism
β-Alanine metabolism
Malonyl-CoA
FASN
FAS1
Fas
ACP
FabD
Acetyl-[acp]
Malonyl-[acp]
FabH
FabY
FAS2
FabB
FabF
FabG
CBR4
HSD17B8
FabA
FabZ
HTD2
FabI
FabK
FabL
FabV
MECR
Acetoacetyl-[acp]
(R)-3-Hydroxy-butanoyl-[acp]
But-2-enoyl-[acp]
Butyryl-[acp]
3-Oxohexanoyl-[acp]
(R)-3-Hydroxy-hexanoyl-[acp]
trans-Hex-2-enoyl-[acp]
Hexanoyl-[acp]
3-Oxooctanoyl-[acp]
(R)-3-Hydroxy-octanoyl-[acp]
trans-Oct-2-enoyl-[acp]
Octanoyl-[acp]
3-Oxodecanoyl-[acp]
(R)-3-Hydroxy-decanoyl-[acp]
trans-Dec-2-enoyl-[acp]
Decanoyl-[acp]
3-Oxo-dodecanoyl-[acp]
(R)-3-Hydroxy-dodecanoyl-[acp]
trans-Dodec-2-enoyl-[acp]
Dodecanoyl-[acp]
3-Oxo-tetradecanoyl-[acp]
(R)-3-Hydroxy-tetradecanoyl-[acp]
trans-Tetradec-2-enoyl-[acp]
Tetradecanoyl-[acp]
3-Oxohexadecanoyl-[acp]
(R)-3-Hydroxy-hexadecanoyl-[acp]
trans-Hexadec-2-enoyl-[acp]
Hexadecanoyl-[acp]
3-Oxooctadecanoyl-[acp]
(R)-3-Hydroxy-octadecanoyl-[acp]
trans-Octadec-2-enoyl-[acp]
Octadecanoyl-[acp]
1.14.19.2
Hexa-decenoyl-[acp]
Octa-decenoyl-[acp]
3.1.2.21
3.1.2.14
5.3.3.14
cis-Dec-3-enoyl-[acp]
Octanoic acid
Decanoic acid
Dodecanoic acid
Tetradecanoic acid
Hexadecanoic acid
Hexadecenoic acid
Octadecanoic acid
Octadecenoic acid
Lipoic acid metabolism
n-7 Unsaturated acyl-[acp]
Fatty acid degradation
6.2.1.3
Hexadecanoyl-CoA
Glycerolipid metabolism
Glycerophospholipid metabolism
Fatty acid elongation
General forms
Type I FAS
(Animals, Fungi, Bacteria)
Type II FAS
(Bacteria, Plants)
In mitochondria (Animals, Fungi)
Initiation
Elongation cycle
Termination
2.3.1.85
FAS1/2
2.3.1.86
Long-chain fatty acid
Long-chain acyl-CoA
2.3.1.39
2.3.1.180
2.3.1.41
2.3.1.179
3-Oxoacyl-[acp]
1.1.1.100
(R)-3-Hydroxy-acyl-[acp]
4.2.1.59
trans-2-Enoyl-[acp]
1.3.1.9
1.3.1.10
1.3.1.104
Long-chain acyl-[acp]
MCT1
MCAT
OXSM
CEM1
OAR1
Acyl-[acp]
Octanoyl-[acp]
Long-chain acyl-[acp] (Hexadecanoyl-[acp])
00061 5/25/20
(c) Kanehisa Laboratories

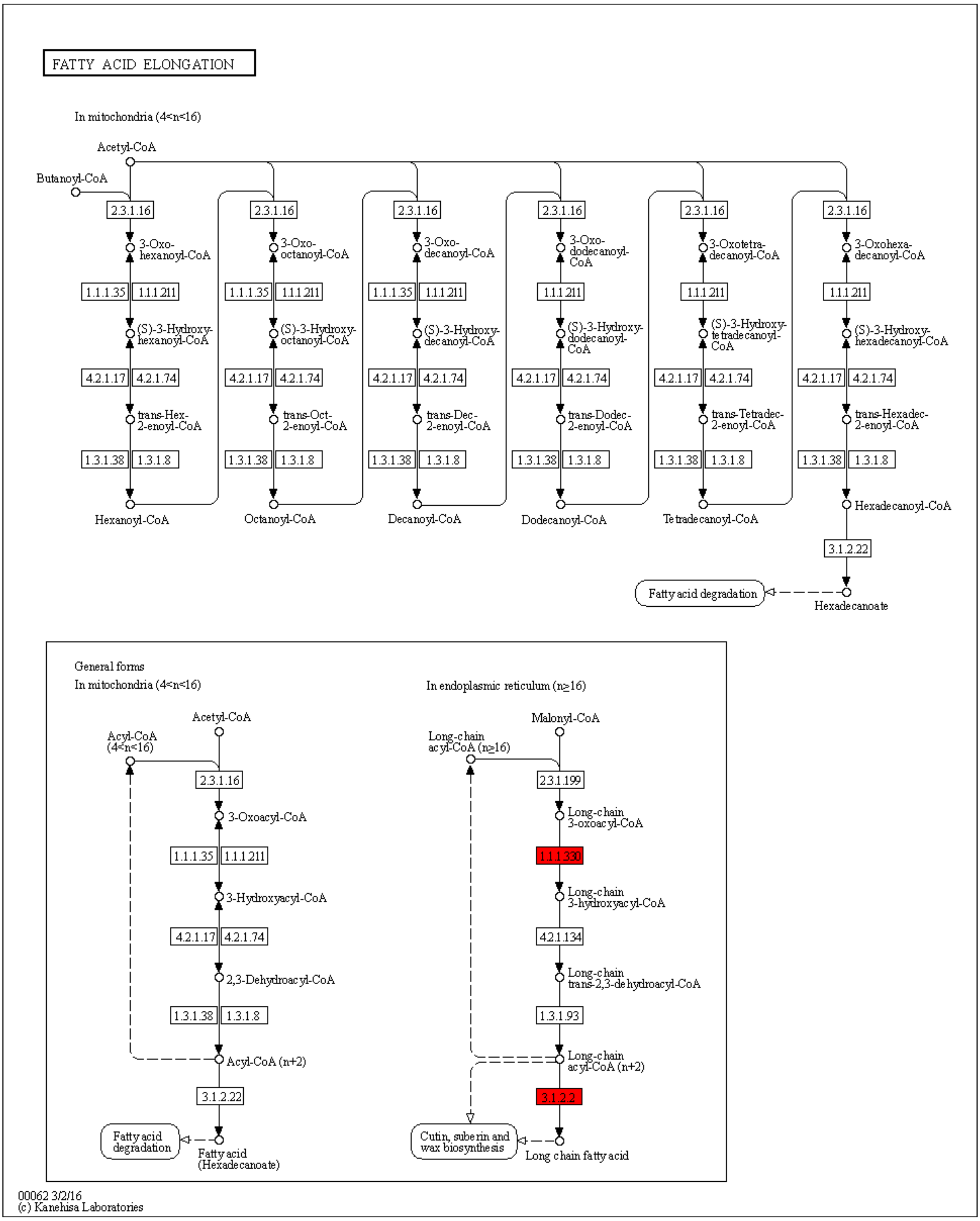
FATTY ACID ELONGATION
In mitochondria (4<n<16)
Acetyl-CoA
Butanoyl-CoA
2.3.1.16
3-Oxo-hexanoyl-CoA
1.1.1.35
1.1.1.211
(S)-3-Hydroxy-hexanoyl-CoA
4.2.1.17
4.2.1.74
trans-Hex-2-enoyl-CoA
1.3.1.38
1.3.1.8
Hexanoyl-CoA
3-Oxo-octanoyl-CoA
(S)-3-Hydroxy-octanoyl-CoA
trans-Oct-2-enoyl-CoA
Octanoyl-CoA
3-Oxo-decanoyl-CoA
(S)-3-Hydroxy-decanoyl-CoA
trans-Dec-2-enoyl-CoA
Decanoyl-CoA
3-Oxo-dodecanoyl-CoA
(S)-3-Hydroxy-dodecanoyl-CoA
trans-Dodec-2-enoyl-CoA
Dodecanoyl-CoA
3-Oxotetra-decanoyl-CoA
(S)-3-Hydroxy-tetradecanoyl-CoA
trans-Tetradec-2-enoyl-CoA
Tetradecanoyl-CoA
3-Oxohexa-decanoyl-CoA
(S)-3-Hydroxy-hexadecanoyl-CoA
trans-Hexadec-2-enoyl-CoA
Hexadecanoyl-CoA
3.1.2.22
Fatty acid degradation
Hexadecanoate
General forms
In mitochondria (4<n<16)
Acetyl-CoA
Acyl-CoA (4<n<16)
2.3.1.16
3-Oxoacyl-CoA
1.1.1.35
1.1.1.211
3-Hydroxyacyl-CoA
4.2.1.17
4.2.1.74
2,3-Dehydroacyl-CoA
1.3.1.38
1.3.1.8
Acyl-CoA (n+2)
3.1.2.22
Fatty acid degradation
Fatty acid (Hexadecanoate)
In endoplasmic reticulum (n≥16)
Malonyl-CoA
Long-chain acyl-CoA (n≥16)
2.3.1.199
Long-chain 3-oxoacyl-CoA
1.1.1.330
Long-chain 3-hydroxyacyl-CoA
4.2.1.134
Long-chain trans-2,3-dehydroacyl-CoA
1.3.1.93
Long-chain acyl-CoA (n+2)
3.1.2.2
Cutin, suberin and wax biosynthesis
Long chain fatty acid
00062 3/2/16
(c) Kanehisa Laboratories

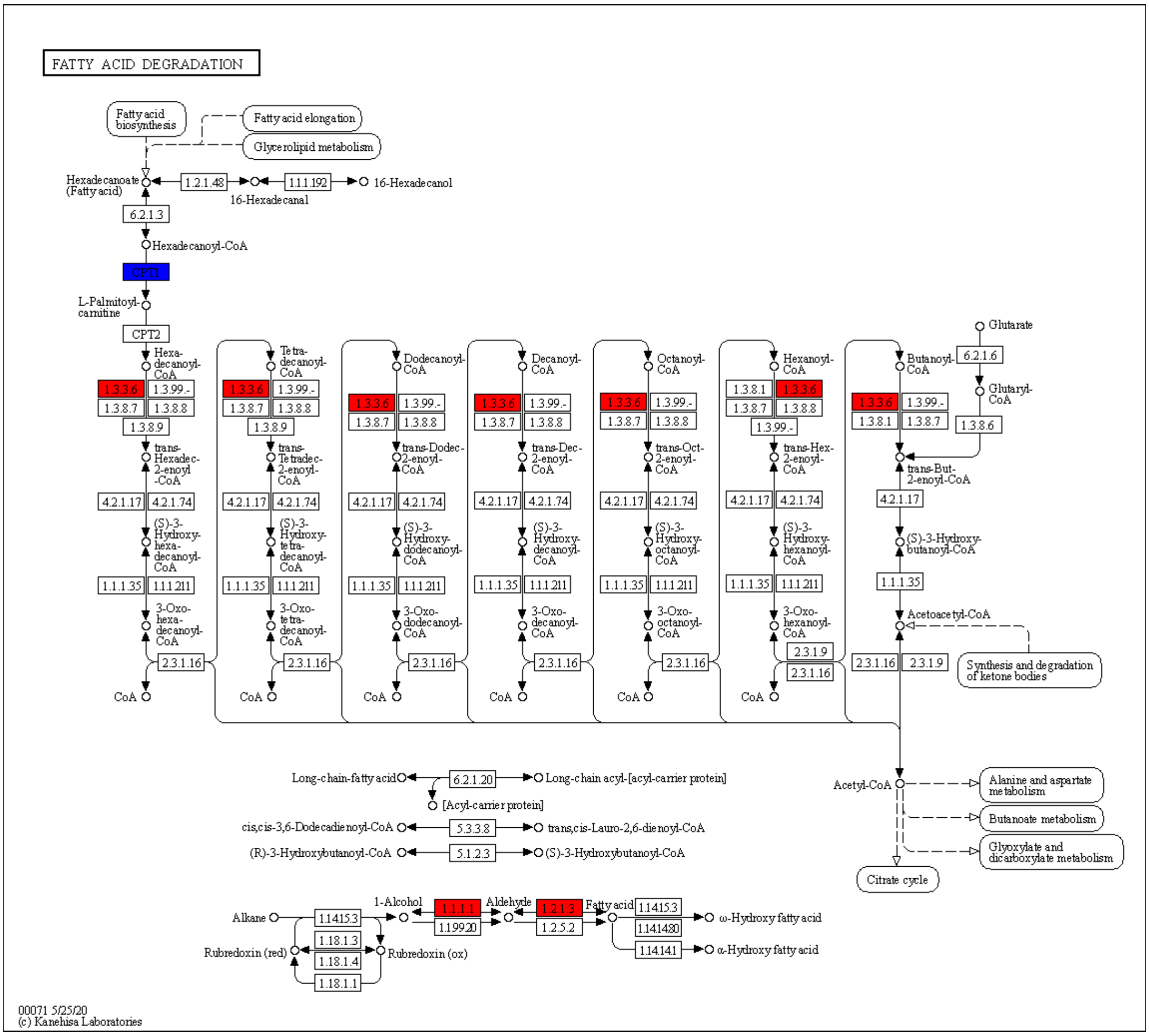
FATTY ACID DEGRADATION
Fatty acid biosynthesis
Fatty acid elongation
Glycerolipid metabolism
Hexadecanoate (Fatty acid)
1.2.1.48
1.1.1.192
16-Hexadecanol
16-Hexadecanal
6.2.1.3
Hexadecanoyl-CoA
CPT1
L-Palmitoyl-carnitine
CPT2
Glutarate
6.2.1.6
Glutaryl-CoA
1.3.8.6
Acetoacetyl-CoA
Synthesis and degradation of ketone bodies
Acetyl-CoA
Alanine and aspartate metabolism
Butanoate metabolism
Glyoxylate and dicarboxylate metabolism
Citrate cycle
Long-chain-fatty acid
6.2.1.20
Long-chain acyl-[acyl-carrier protein]
[Acyl-carrier protein]
cis,cis-3,6-Dodecadienoyl-CoA
5.3.3.8
trans,cis-Lauro-2,6-dienoyl-CoA
(R)-3-Hydroxybutanoyl-CoA
5.1.2.3
(S)-3-Hydroxybutanoyl-CoA
Alkane
1.14.15.3
1.18.1.3
1.18.1.4
1.18.1.1
Rubredoxin (red)
Rubredoxin (ox)
1-Alcohol
1.1.1.1
1.1.99.20
Aldehyde
1.2.1.3
1.2.5.2
Fatty acid
1.14.14.80
1.14.14.1
ω-Hydroxy fatty acid
α-Hydroxy fatty acid
00071 5/25/20
(c) Kanehisa Laboratories

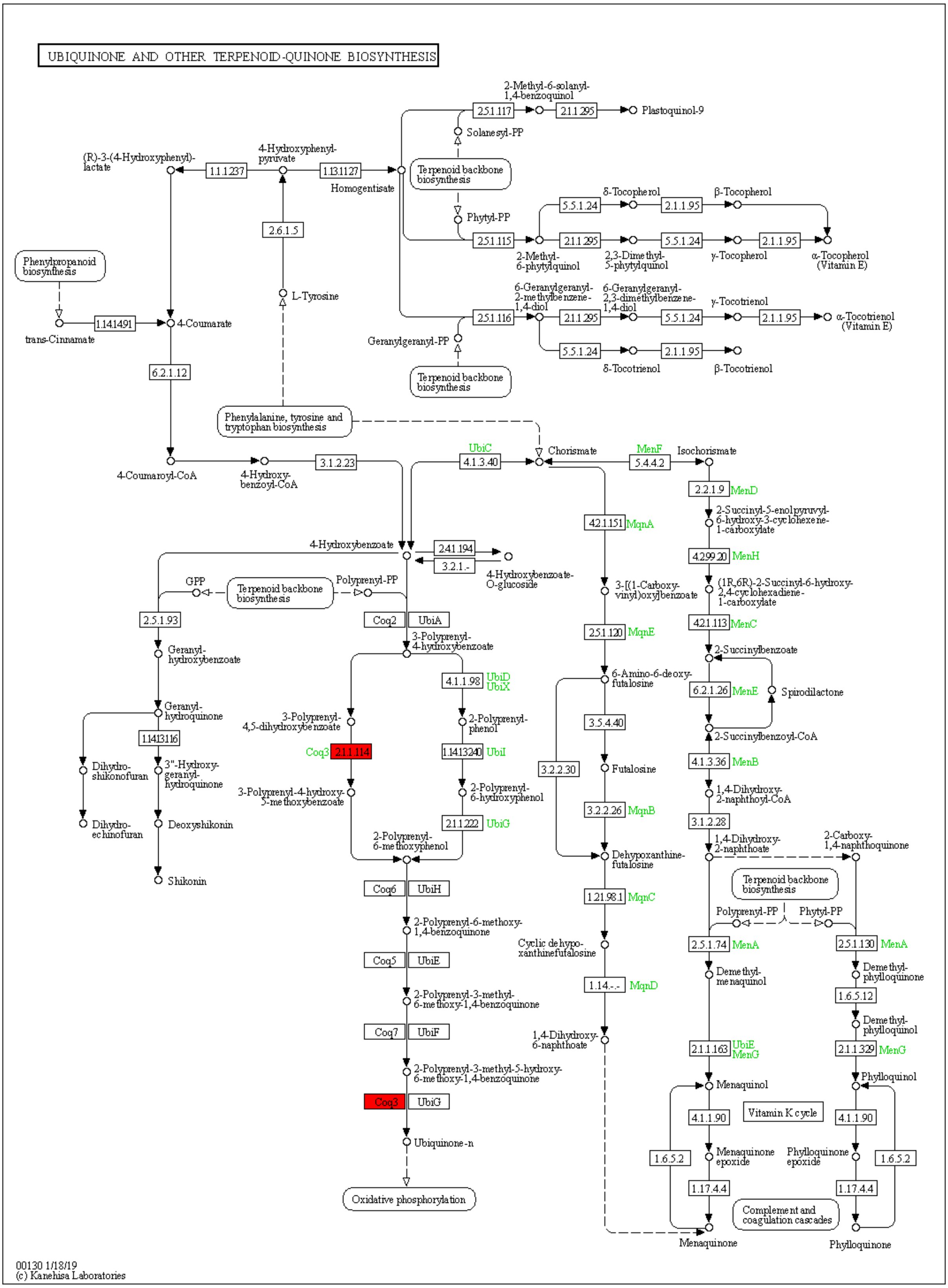
UBIQUINONE AND OTHER TERPENOID-QUINONE BIOSYNTHESIS
2-Methyl-6-solanyl-1,4-benzoquinol
2.5.1.117
2.1.1.295
Plastoquinol-9
Solanesyl-PP
(R)-3-(4-Hydroxyphenyl)-lactate
1.1.1.237
4-Hydroxyphenyl-pyruvate
1.13.11.27
Homogentisate
Terpenoid backbone biosynthesis
δ-Tocopherol
β-Tocopherol
5.5.1.24
2.1.1.95
Phytyl-PP
2.6.1.5
2.5.1.115
2.1.1.295
5.5.1.24
2.1.1.95
2-Methyl-6-phytylquinol
2,3-Dimethyl-5-phytylquinol
γ-Tocopherol
α-Tocopherol (Vitamin E)
Phenylpropanoid biosynthesis
L-Tyrosine
6-Geranylgeranyl-2-methylbenzene-1,4-diol
6-Geranylgeranyl-2,3-dimethylbenzene-1,4-diol
γ-Tocotrienol
1.14.14.91
4-Coumarate
2.5.1.116
2.1.1.295
5.5.1.24
2.1.1.95
α-Tocotrienol (Vitamin E)
trans-Cinnamate
Geranylgeranyl-PP
6.2.1.12
5.5.1.24
2.1.1.95
δ-Tocotrienol
β-Tocotrienol
Terpenoid backbone biosynthesis
Phenylalanine, tyrosine and tryptophan biosynthesis
UbiC
MenF
Chorismate
Isochorismate
4.1.3.40
5.4.4.2
4-Coumaroyl-CoA
4-Hydroxy-benzoyl-CoA
3.1.2.23
2.2.1.9
MenD
2-Succinyl-5-enolpyruvyl-6-hydroxy-3-cyclohexene-1-carboxylate
4.2.1.151
MqnA
4-Hydroxybenzoate
2.4.1.194
3.2.1.-
4-Hydroxybenzoate-O-glucoside
4.2.99.20
MenH
GPP
Terpenoid backbone biosynthesis
Polyprenyl-PP
3-[(1-Carboxy-vinyl)oxy]benzoate
(1R,6R)-2-Succinyl-6-hydroxy-2,4-cyclohexadiene-1-carboxylate
2.5.1.93
Coq2
UbiA
4.2.1.113
MenC
3-Polyprenyl-4-hydroxybenzoate
2.5.1.120
MqnE
Geranyl-hydroxybenzoate
2-Succinylbenzoate
6-Amino-6-deoxy-futalosine
4.1.1.98
UbiD
UbiX
6.2.1.26
MenE
Spirodilactone
Geranyl-hydroquinone
3-Polyprenyl-4,5-dihydroxybenzoate
2-Polyprenyl-phenol
3.5.4.40
2-Succinylbenzoyl-CoA
1.14.13.116
Coq3
2.1.1.114
1.14.13.240
UbiI
Dihydro-shikonofuran
3''-Hydroxy-geranyl-hydroquinone
3.2.2.30
Futalosine
4.1.3.36
MenB
3-Polyprenyl-4-hydroxy-5-methoxybenzoate
2-Polyprenyl-6-hydroxyphenol
1,4-Dihydroxy-2-naphthoyl-CoA
3.2.2.26
MqnB
Dihydro-echinofuran
Deoxyshikonin
2.1.1.222
UbiG
3.1.2.28
2-Polyprenyl-6-methoxyphenol
1,4-Dihydroxy-2-naphthoate
2-Carboxy-1,4-naphthoquinone
Dehypoxanthine-futalosine
Shikonin
Coq6
UbiH
Terpenoid backbone biosynthesis
1.21.98.1
MqnC
Polyprenyl-PP
Phytyl-PP
2-Polyprenyl-6-methoxy-1,4-benzoquinone
Cyclic dehypo-xanthinefutalosine
2.5.1.74
MenA
2.5.1.130
MenA
Coq5
UbiE
Demethyl-menaquinol
Demethyl-phylloquinone
1.14.-.-
MqnD
2-Polyprenyl-3-methyl-6-methoxy-1,4-benzoquinone
1.6.5.12
Demethyl-phylloquinol
Coq7
UbiF
1,4-Dihydroxy-6-naphthoate
2.1.1.163
UbiE
MenG
2.1.1.329
MenG
2-Polyprenyl-3-methyl-5-hydroxy-6-methoxy-1,4-benzoquinone
Menaquinol
Phylloquinol
Coq3
UbiG
Vitamin K cycle
4.1.1.90
4.1.1.90
Ubiquinone-n
1.6.5.2
Menaquinone epoxide
Phylloquinone epoxide
1.6.5.2
1.17.4.4
1.17.4.4
Oxidative phosphorylation
Complement and coagulation cascades
Menaquinone
Phylloquinone
00130 1/18/19
(c) Kanehisa Laboratories

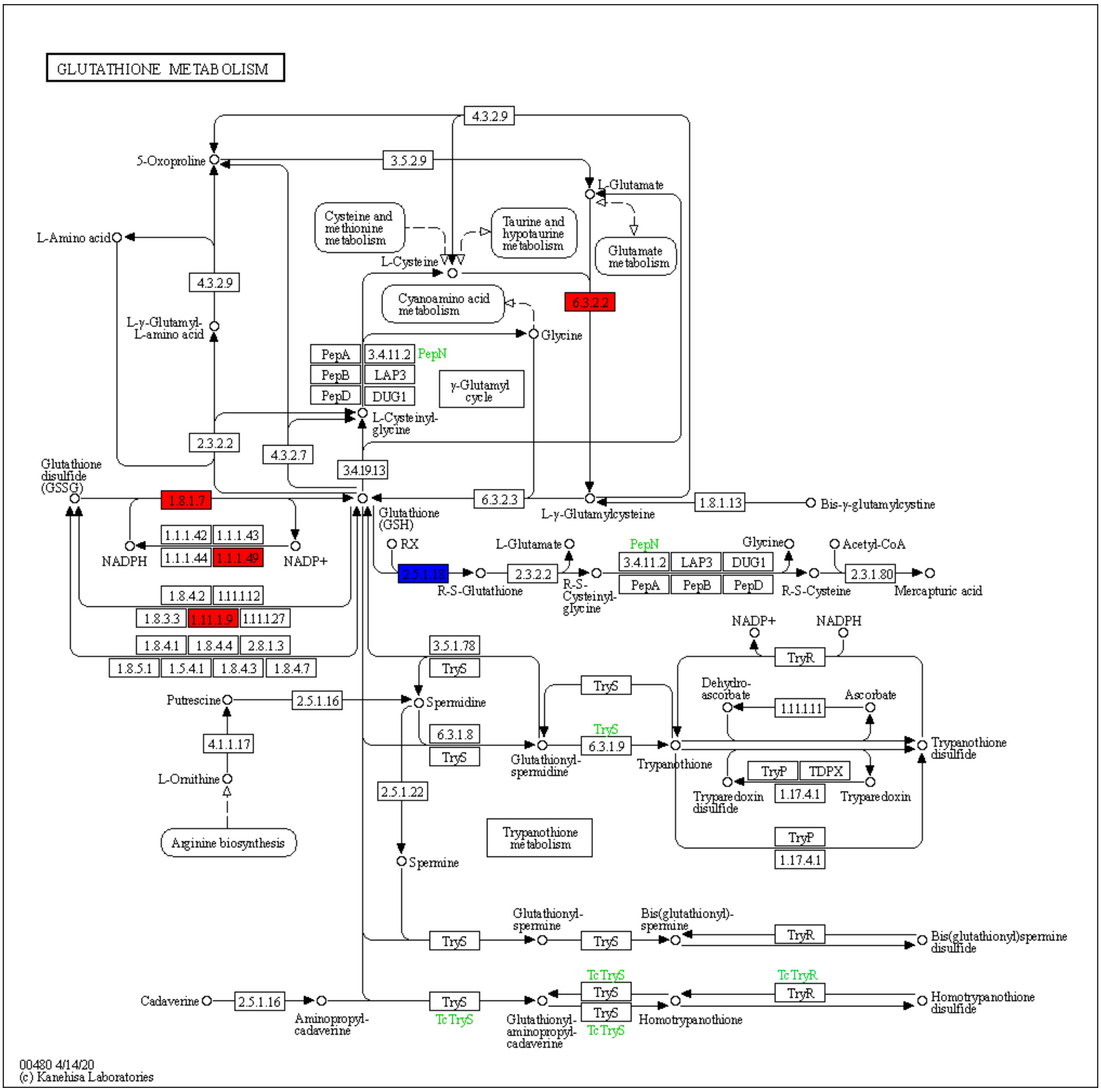
GLUTATHIONE METABOLISM
4.3.2.9
5-Oxoproline
3.5.2.9
L-Glutamate
Cysteine and methionine metabolism
Taurine and hypotaurine metabolism
Glutamate metabolism
L-Amino acid
4.3.2.9
L-Cysteine
Cyanoamino acid metabolism
6.3.2.2
L-γ-Glutamyl-L-amino acid
Glycine
PepA
3.4.11.2
PepN
PepB
LAP3
γ-Glutamyl cycle
PepD
DUG1
L-Cysteinyl-glycine
2.3.2.2
4.3.2.7
Glutathione disulfide (GSSG)
3.4.19.13
6.3.2.3
1.8.1.7
Glutathione (GSH)
L-γ-Glutamylcysteine
1.8.1.13
Bis-γ-glutamylcystine
1.1.1.42
1.1.1.43
NADPH
1.1.1.44
1.1.1.49
NADP+
RX
L-Glutamate
PepN
Glycine
Acetyl-CoA
2.5.1.18
2.3.2.2
3.4.11.2
LAP3
DUG1
2.3.1.80
R-S-Glutathione
R-S-Cysteinyl-glycine
PepA
PepB
PepD
R-S-Cysteine
Mercapturic acid
1.8.4.2
1.11.1.12
1.8.3.3
1.11.1.9
1.11.1.27
NADP+
NADPH
1.8.4.1
1.8.4.4
2.8.1.3
3.5.1.78
TryR
1.8.5.1
1.5.4.1
1.8.4.3
1.8.4.7
TryS
TryS
Dehydro-ascorbate
Ascorbate
Putrescine
2.5.1.16
Spermidine
1.11.1.11
4.1.1.17
TryS
6.3.1.8
6.3.1.9
Trypanothione disulfide
TryS
Glutathionyl-spermidine
Trypanothione
L-Ornithine
TryP
TDPX
2.5.1.22
Tryparedoxin disulfide
1.17.4.1
Tryparedoxin
Arginine biosynthesis
Trypanothione metabolism
TryP
1.17.4.1
Spermine
Glutathionyl-spermine
Bis(glutathionyl)-spermine
TryS
TryS
TryR
Bis(glutathionyl)spermine disulfide
TcTryS
TcTryR
Cadaverine
2.5.1.16
TryS
TryS
TryR
Homotrypanothione disulfide
TryS
Aminopropyl-cadaverine
TcTryS
Glutathionyl-aminopropyl-cadaverine
TcTryS
Homotrypanothione
00480 4/14/20
(c) Kanehisa Laboratories

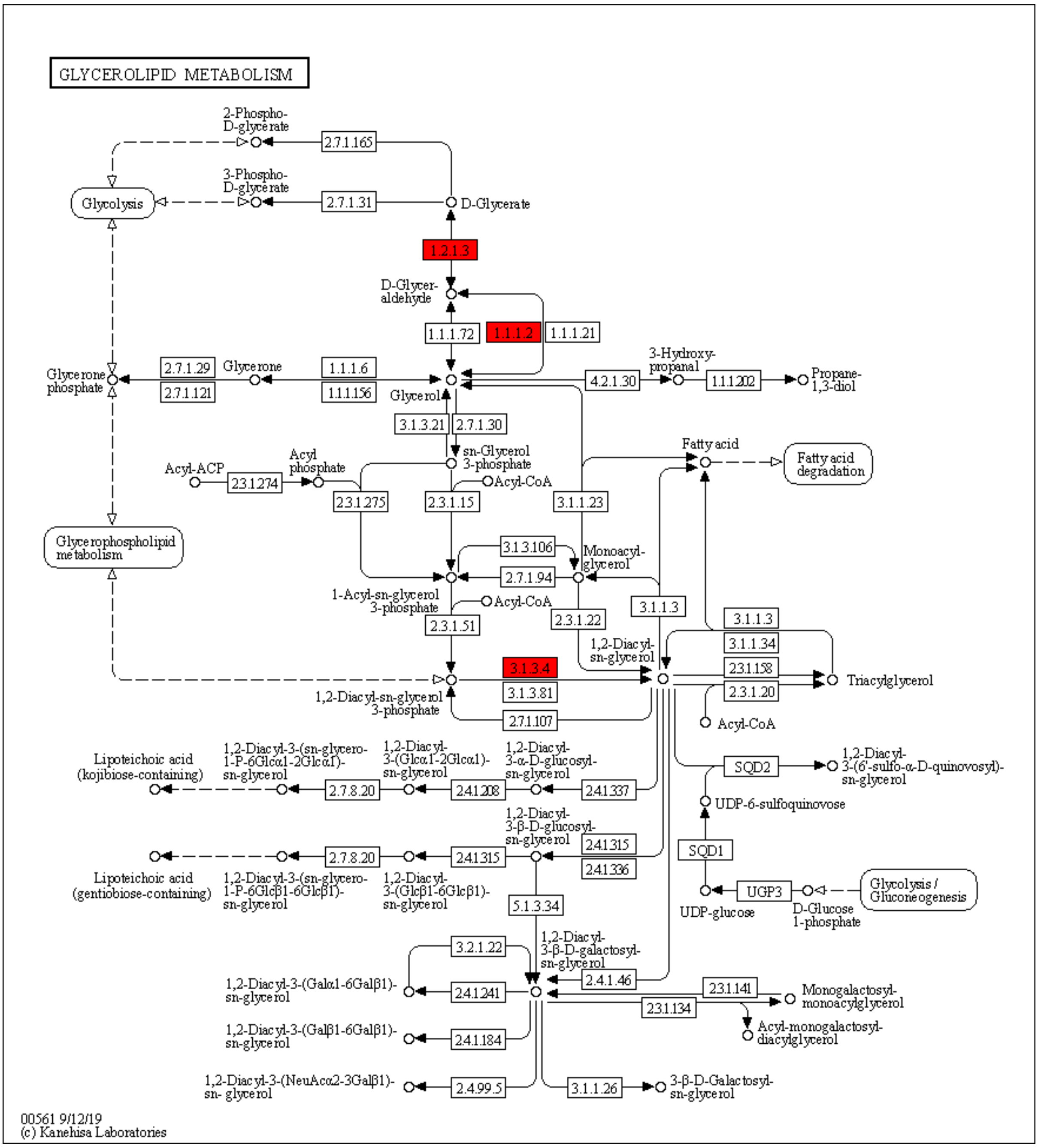
GLYCEROLIPID METABOLISM
2-Phospho-D-glycerate
2.7.1.165
3-Phospho-D-glycerate
Glycolysis
2.7.1.31
D-Glycerate
1.2.1.3
D-Glycer-aldehyde
1.1.1.72
1.1.1.2
1.1.1.21
3-Hydroxy-propanal
Glycerone phosphate
2.7.1.29
2.7.1.121
Glycerone
1.1.1.6
1.1.1.156
Glycerol
4.2.1.30
1.1.1.202
Propane-1,3-diol
3.1.3.21
2.7.1.30
sn-Glycerol 3-phosphate
Acyl-ACP
2.3.1.274
Acyl phosphate
2.3.1.275
2.3.1.15
Acyl-CoA
3.1.1.23
Fatty acid
Fatty acid degradation
Glycerophospholipid metabolism
3.1.3.106
Monoacyl-glycerol
2.7.1.94
1-Acyl-sn-glycerol 3-phosphate
Acyl-CoA
3.1.1.3
2.3.1.22
2.3.1.51
3.1.1.3
1,2-Diacyl-sn-glycerol
3.1.1.34
3.1.3.4
2.3.1.158
Triacylglycerol
3.1.3.81
2.3.1.20
1,2-Diacyl-sn-glycerol 3-phosphate
2.7.1.107
Acyl-CoA
Lipoteichoic acid (kojibiose-containing)
1,2-Diacyl-3-(sn-glycero-1-P-6Glcα1-2Glcα1)-sn-glycerol
1,2-Diacyl-3-(Glcα1-2Glcα1)-sn-glycerol
1,2-Diacyl-3-α-D-glucosyl-sn-glycerol
2.7.8.20
2.4.1.208
2.4.1.337
SQD2
1,2-Diacyl-3-(6'-sulfo-α-D-quinovosyl)-sn-glycerol
UDP-6-sulfoquinovose
1,2-Diacyl-3-β-D-glucosyl-sn-glycerol
2.4.1.315
2.7.8.20
2.4.1.315
2.4.1.336
SQD1
Lipoteichoic acid (gentiobiose-containing)
1,2-Diacyl-3-(sn-glycero-1-P-6Glcβ1-6Glcβ1)-sn-glycerol
1,2-Diacyl-3-(Glcβ1-6Glcβ1)-sn-glycerol
UGP3
Glycolysis / Gluconeogenesis
5.1.3.34
UDP-glucose
D-Glucose 1-phosphate
3.2.1.22
1,2-Diacyl-3-β-D-galactosyl-sn-glycerol
1,2-Diacyl-3-(Galα1-6Galβ1)-sn-glycerol
2.4.1.241
2.4.1.46
2.3.1.141
Monogalactosyl-monoacylglycerol
2.3.1.134
Acyl-monogalactosyl-diacylglycerol
1,2-Diacyl-3-(Galβ1-6Galβ1)-sn-glycerol
2.4.1.184
1,2-Diacyl-3-(NeuAcα2-3Galβ1)-sn- glycerol
2.4.99.5
3.1.1.26
3-β-D-Galactosyl-sn-glycerol
00561 9/12/19
(c) Kanehisa Laboratories

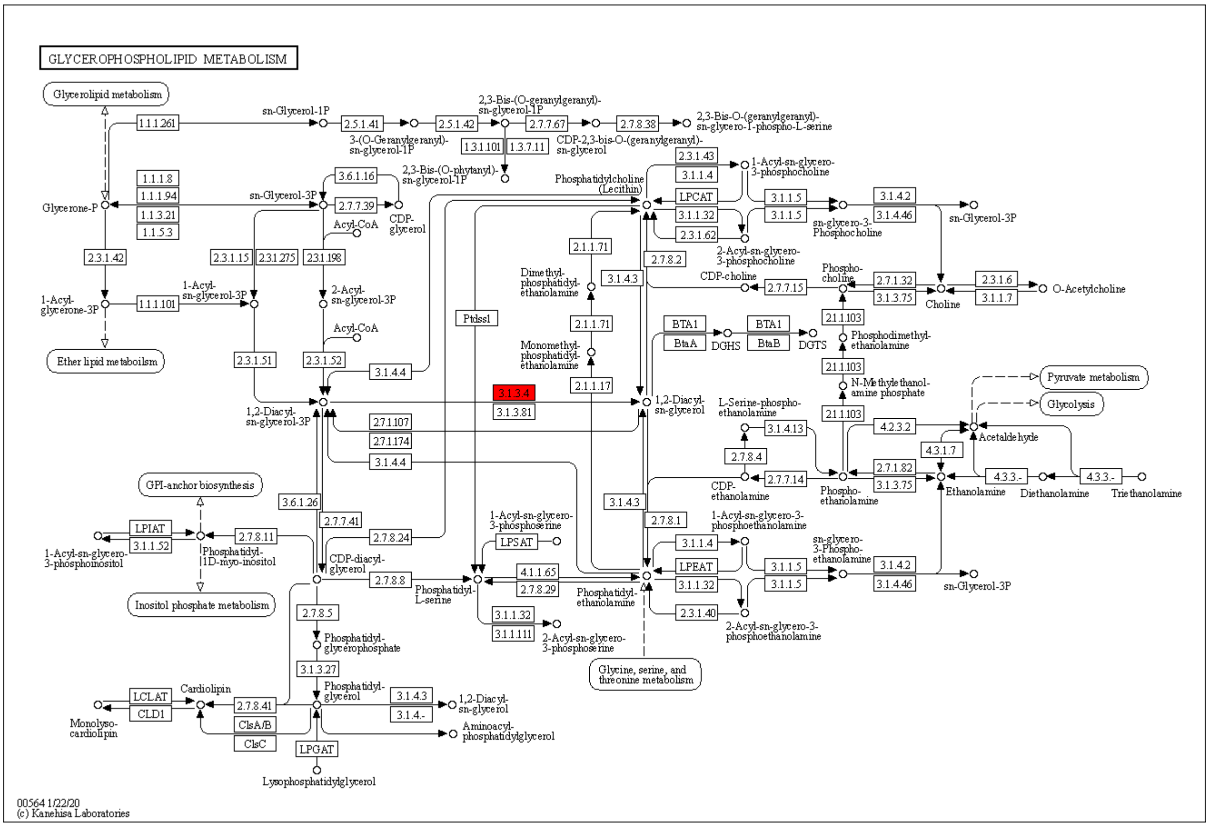
GLYCEROPHOSPHOLIPID METABOLISM
Glycerolipid metabolism
Ether lipid metaboilsm
GPI-anchor biosynthesis
Inositol phosphate metabolism
Glycine, serine, and threonine metabolism
Pyruvate metabolism
Glycolysis
sn-Glycerol-1P
2,3-Bis-(O-geranylgeranyl)-sn-glycerol-1P
2,3-Bis-O-(geranylgeranyl)-sn-glycero-1-phospho-L-serine
3-(O-Geranylgeranyl)-sn-glycerol-1P
CDP-2,3-bis-O-(geranylgeranyl)-sn-glycerol
2,3-Bis-(O-phytanyl)-sn-glycerol-1P
Glycerone-P
sn-Glycerol-3P
CDP-glycerol
Acyl-CoA
1-Acyl-glycerone-3P
1-Acyl-sn-glycerol-3P
2-Acyl-sn-glycerol-3P
1,2-Diacyl-sn-glycerol-3P
Phosphatidylcholine (Lecithin)
1-Acyl-sn-glycero-3-phosphocholine
sn-glycero-3-Phosphocholine
2-Acyl-sn-glycero-3-phosphocholine
sn-Glycerol-3P
CDP-choline
Phospho-choline
Choline
O-Acetylcholine
Dimethyl-phosphatidyl-ethanolamine
Monomethyl-phosphatidyl-ethanolamine
DGHS
DGTS
Phosphodimethyl-ethanolamine
N-Methylethanol-amine phosphate
1,2-Diacyl-sn-glycerol
L-Serine-phospho-ethanolamine
Acetaldehyde
CDP-ethanolamine
Phospho-ethanolamine
Ethanolamine
Diethanolamine
Triethanolamine
1-Acyl-sn-glycero-3-phosphoserine
Phosphatidyl-L-serine
2-Acyl-sn-glycero-3-phosphoserine
Phosphatidyl-ethanolamine
1-Acyl-sn-glycero-3-phosphoethanolamine
sn-glycero-3-Phospho-ethanolamine
2-Acyl-sn-glycero-3-phosphoethanolamine
1-Acyl-sn-glycero-3-phosphoinositol
Phosphatidyl-1D-myo-inositol
CDP-diacyl-glycerol
Phosphatidyl-glycerophosphate
Phosphatidyl-glycerol
Cardiolipin
Monolyso-cardiolipin
1,2-Diacyl-sn-glycerol
Aminoacyl-phosphatidylglycerol
Lysophosphatidylglycerol
1.1.1.261
2.5.1.41
2.5.1.42
2.7.7.67
2.7.8.38
1.3.1.101
1.3.7.11
1.1.1.8
1.1.1.94
1.1.3.21
1.1.5.3
3.6.1.16
2.7.7.39
2.3.1.42
2.3.1.15
2.3.1.275
2.3.1.198
1.1.1.101
2.3.1.51
2.3.1.52
3.1.4.4
Ptdss1
3.1.3.4
3.1.3.81
2.7.1.107
2.7.1.174
3.6.1.26
2.7.7.41
2.7.8.24
2.7.8.11
LPIAT
3.1.1.52
2.7.8.8
2.7.8.5
3.1.3.27
2.7.8.41
LCLAT
CLD1
ClsA/B
ClsC
LPGAT
3.1.4.3
3.1.4.-
2.3.1.43
3.1.1.4
LPCAT
3.1.1.32
2.3.1.62
3.1.1.5
3.1.4.2
3.1.4.46
2.1.1.71
2.7.8.2
2.7.7.15
2.7.1.32
3.1.3.75
2.3.1.6
3.1.1.7
BTA1
BtaA
BtaB
2.1.1.103
2.1.1.17
3.1.4.13
2.7.8.4
2.7.7.14
4.2.3.2
4.3.1.7
2.7.1.82
4.3.3.-
2.7.8.1
LPSAT
4.1.1.65
2.7.8.29
3.1.1.111
LPEAT
2.3.1.40
00564 1/22/20
(c) Kanehisa Laboratories

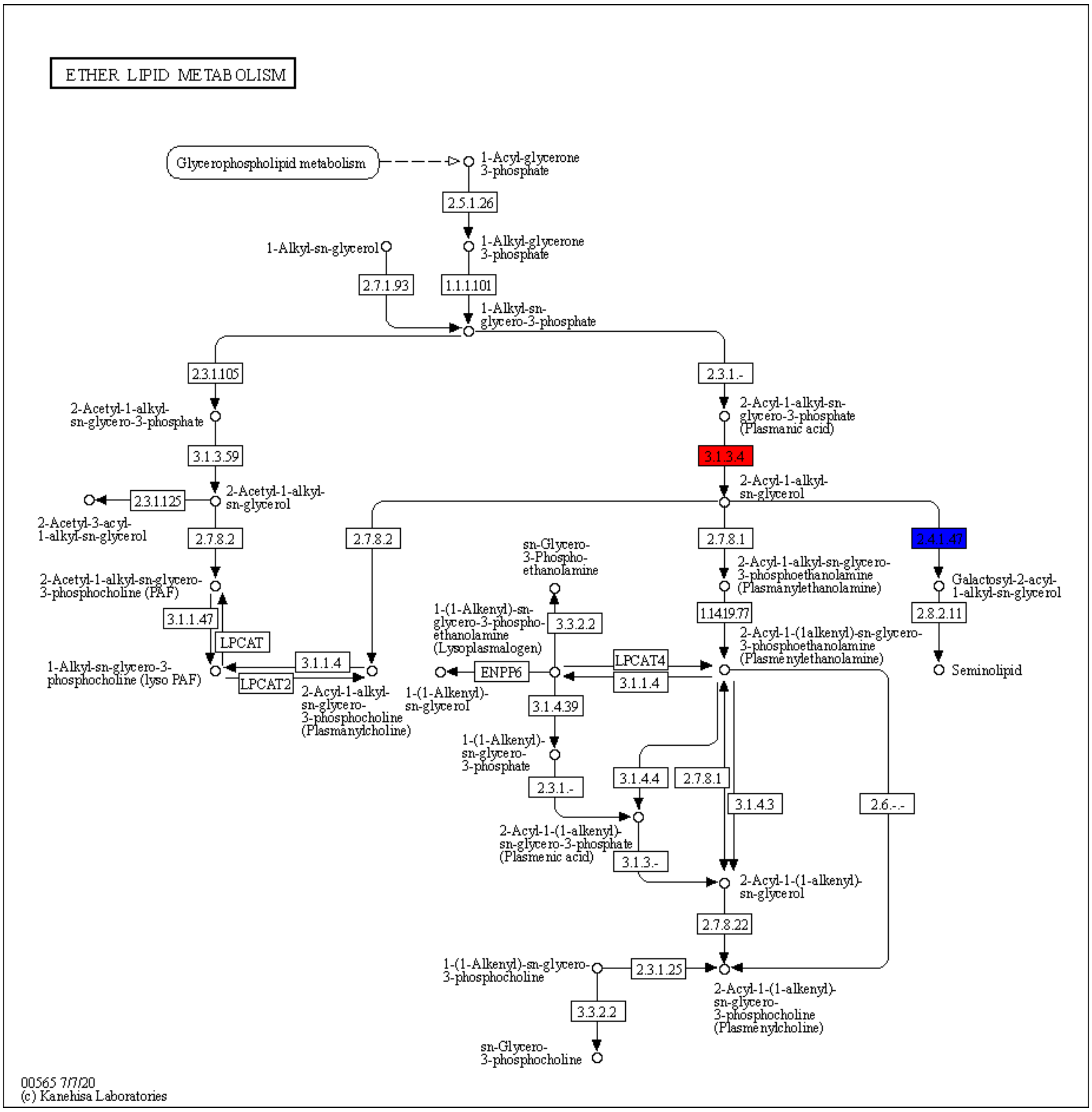
ETHER LIPID METABOLISM
Glycerophospholipid metabolism
1-Acyl-glycerone 3-phosphate
2.5.1.26
1-Alkyl-sn-glycerol
1-Alkyl-glycerone 3-phosphate
2.7.1.93
1.1.1.101
1-Alkyl-sn-glycero-3-phosphate
2.3.1.105
2.3.1.-
2-Acetyl-1-alkyl-sn-glycero-3-phosphate
2-Acyl-1-alkyl-sn-glycero-3-phosphate (Plasmanic acid)
3.1.3.59
3.1.3.4
2.3.1.125
2-Acetyl-1-alkyl-sn-glycerol
2-Acyl-1-alkyl-sn-glycerol
2-Acetyl-3-acyl-1-alkyl-sn-glycerol
2.7.8.2
2.7.8.2
2.7.8.1
2.4.1.47
sn-Glycero-3-Phospho-ethanolamine
2-Acyl-1-alkyl-sn-glycero-3-phosphoethanolamine (Plasmanylethanolamine)
Galactosyl-2-acyl-1-alkyl-sn-glycerol
2-Acetyl-1-alkyl-sn-glycero-3-phosphocholine (PAF)
1-(1-Alkenyl)-sn-glycero-3-phospho-ethanolamine (Lysoplasmalogen)
3.3.2.2
1.14.19.77
2.8.2.11
3.1.1.47
LPCAT
2-Acyl-1-(1alkenyl)-sn-glycero-3-phosphoethanolamine (Plasmenylethanolamine)
LPCAT4
1-Alkyl-sn-glycero-3-phosphocholine (lyso PAF)
3.1.1.4
ENPP6
3.1.1.4
Seminolipid
LPCAT2
2-Acyl-1-alkyl-sn-glycero-3-phosphocholine (Plasmanylcholine)
1-(1-Alkenyl)-sn-glycerol
3.1.4.39
1-(1-Alkenyl)-sn-glycero-3-phosphate
3.1.4.4
2.7.8.1
3.1.4.3
2.6.-.-
2.3.1.-
2-Acyl-1-(1-alkenyl)-sn-glycero-3-phosphate (Plasmenic acid)
3.1.3.-
2-Acyl-1-(1-alkenyl)-sn-glycerol
2.7.8.22
1-(1-Alkenyl)-sn-glycero-3-phosphocholine
2.3.1.25
2-Acyl-1-(1-alkenyl)-sn-glycero-3-phosphocholine (Plasmenylcholine)
3.3.2.2
sn-Glycero-3-phosphocholine
00565 7/7/20
(c) Kanehisa Laboratories

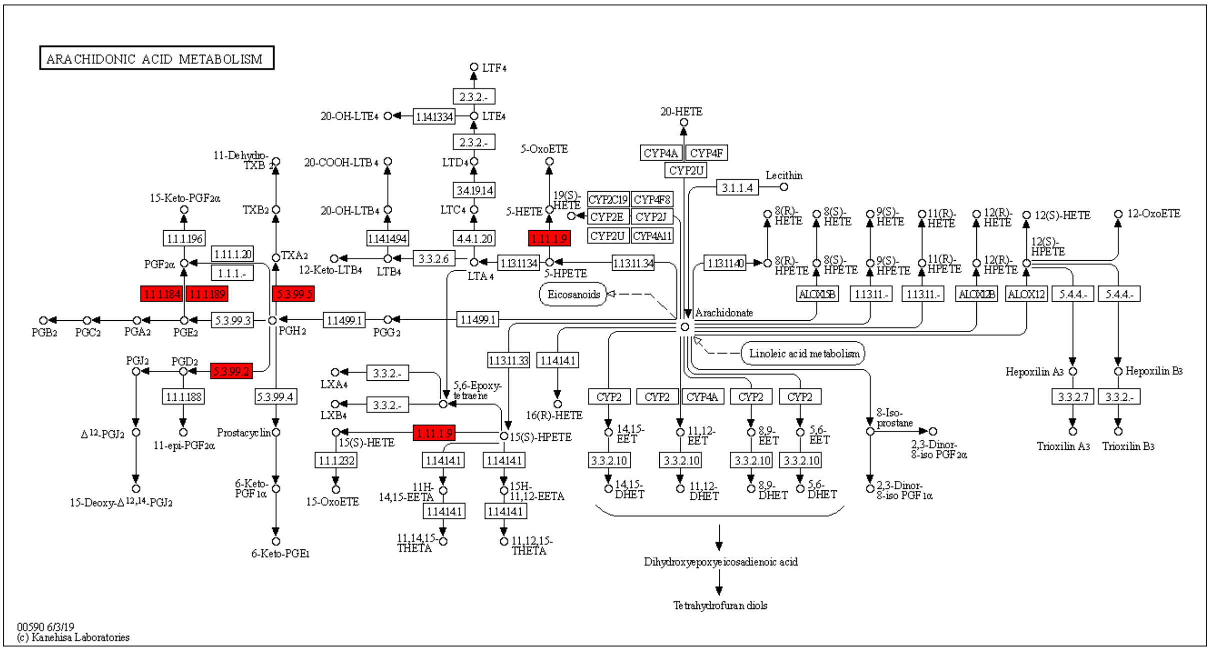
ARACHIDONIC ACID METABOLISM
LTF4
2.3.2.-
20-OH-LTE4
1.14.13.34
LTE4
2.3.2.-
20-HETE
CYP4A
CYP4F
CYP2U
11-Dehydro-TXB2
20-COOH-LTB4
LTD4
5-OxoETE
3.1.1.4
Lecithin
15-Keto-PGF2α
TXB2
20-OH-LTB4
3.4.19.14
LTC4
5-HETE
19(S)-HETE
CYP2C19
CYP4F8
CYP2E
CYP2J
CYP2U
CYP4A11
8(R)-HETE
8(S)-HETE
9(S)-HETE
11(R)-HETE
12(R)-HETE
12(S)-HETE
12-OxoETE
1.1.1.196
1.1.1.20
1.14.14.94
4.4.1.20
1.11.1.9
TXA2
PGF2α
1.1.1.-
3.3.2.6
12-Keto-LTB4
LTB4
1.13.11.34
5-HPETE
LTA4
1.13.11.34
1.13.11.40
8(R)-HPETE
8(S)-HPETE
9(S)-HPETE
11(R)-HPETE
12(R)-HPETE
12(S)-HPETE
1.11.1.184
1.11.1.189
5.3.99.5
Eicosanoids
ALOX15B
1.13.11.-
1.13.11.-
ALOX12B
ALOX12
5.4.4.-
5.4.4.-
PGB2
PGC2
PGA2
PGE2
5.3.99.3
PGH2
1.14.99.1
PGG2
1.14.99.1
Arachidonate
1.13.11.33
1.14.14.1
Linoleic acid metabolism
PGJ2
PGD2
5.3.99.2
3.3.2.-
LXA4
5,6-Epoxy-tetraene
Hepoxilin A3
Hepoxilin B3
1.1.1.188
5.3.99.4
3.3.2.-
CYP2
CYP2
CYP4A
CYP2
CYP2
3.3.2.7
3.3.2.-
Δ12-PGJ2
11-epi-PGF2α
Prostacyclin
LXB4
1.11.1.9
15(S)-HPETE
16(R)-HETE
14,15-EET
11,12-EET
8,9-EET
5,6-EET
8-Iso-prostane
2,3-Dinor-8-iso PGF2α
Trioxilin A3
Trioxilin B3
15(S)-HETE
1.1.1.232
1.14.14.1
1.14.14.1
3.3.2.10
3.3.2.10
3.3.2.10
3.3.2.10
6-Keto-PGF1α
15-OxoETE
11H-14,15-EETA
15H-11,12-EETA
14,15-DHET
11,12-DHET
8,9-DHET
5,6-DHET
2,3-Dinor-8-iso PGF1α
15-Deoxy-Δ12,14-PGJ2
1.14.14.1
1.14.14.1
11,14,15-THETA
11,12,15-THETA
6-Keto-PGE1
Dihydroxyepoxyeicosadienoic acid
Tetrahydrofuran diols
00590 6/3/19
(c) Kanehisa Laboratories

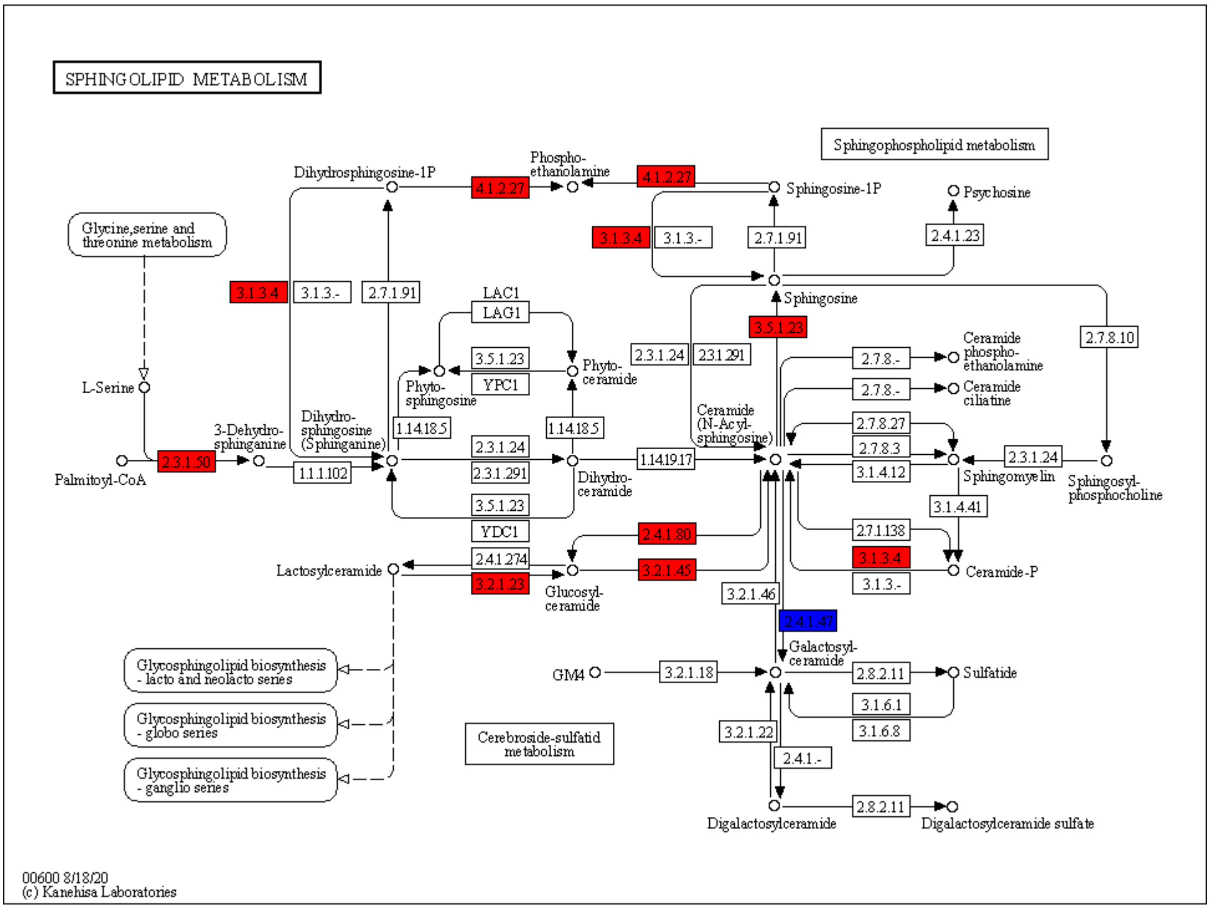
SPHINGOLIPID METABOLISM
Sphingophospholipid metabolism
Dihydrosphingosine-1P
Phospho-ethanolamine
4.1.2.27
4.1.2.27
Sphingosine-1P
Psychosine
Glycine, serine and threonine metabolism
3.1.3.4
3.1.3.-
2.7.1.91
2.4.1.23
3.1.3.4
3.1.3.-
2.7.1.91
LAC1
LAG1
Sphingosine
3.5.1.23
2.7.8.10
3.5.1.23
Phyto-ceramide
2.3.1.24
2.3.1.291
2.7.8.-
Ceramide phospho-ethanolamine
L-Serine
YPC1
Phyto-sphingosine
2.7.8.-
Ceramide ciliatine
Ceramide (N-Acyl-sphingosine)
3-Dehydro-sphinganine
Dihydro-sphingosine (Sphinganine)
1.14.18.5
1.14.18.5
2.7.8.27
2.7.8.3
Palmitoyl-CoA
2.3.1.50
1.1.1.102
2.3.1.24
2.3.1.291
1.14.19.17
3.1.4.12
2.3.1.24
Sphingomyelin
Sphingosyl-phosphocholine
Dihydro-ceramide
3.5.1.23
3.1.4.41
YDC1
2.4.1.80
2.7.1.138
Lactosylceramide
2.4.1.274
3.2.1.45
3.1.3.4
Ceramide-P
3.2.1.23
Glucosyl-ceramide
3.2.1.46
3.1.3.-
2.4.1.47
Galactosyl-ceramide
Glycosphingolipid biosynthesis - lacto and neolacto series
GM4
3.2.1.18
2.8.2.11
Sulfatide
3.1.6.1
Glycosphingolipid biosynthesis - globo series
Cerebroside-sulfatid metabolism
3.2.1.22
3.1.6.8
2.4.1.-
Glycosphingolipid biosynthesis - ganglio series
Digalactosylceramide
2.8.2.11
Digalactosylceramide sulfate
00600 8/18/20
(c) Kanehisa Laboratories

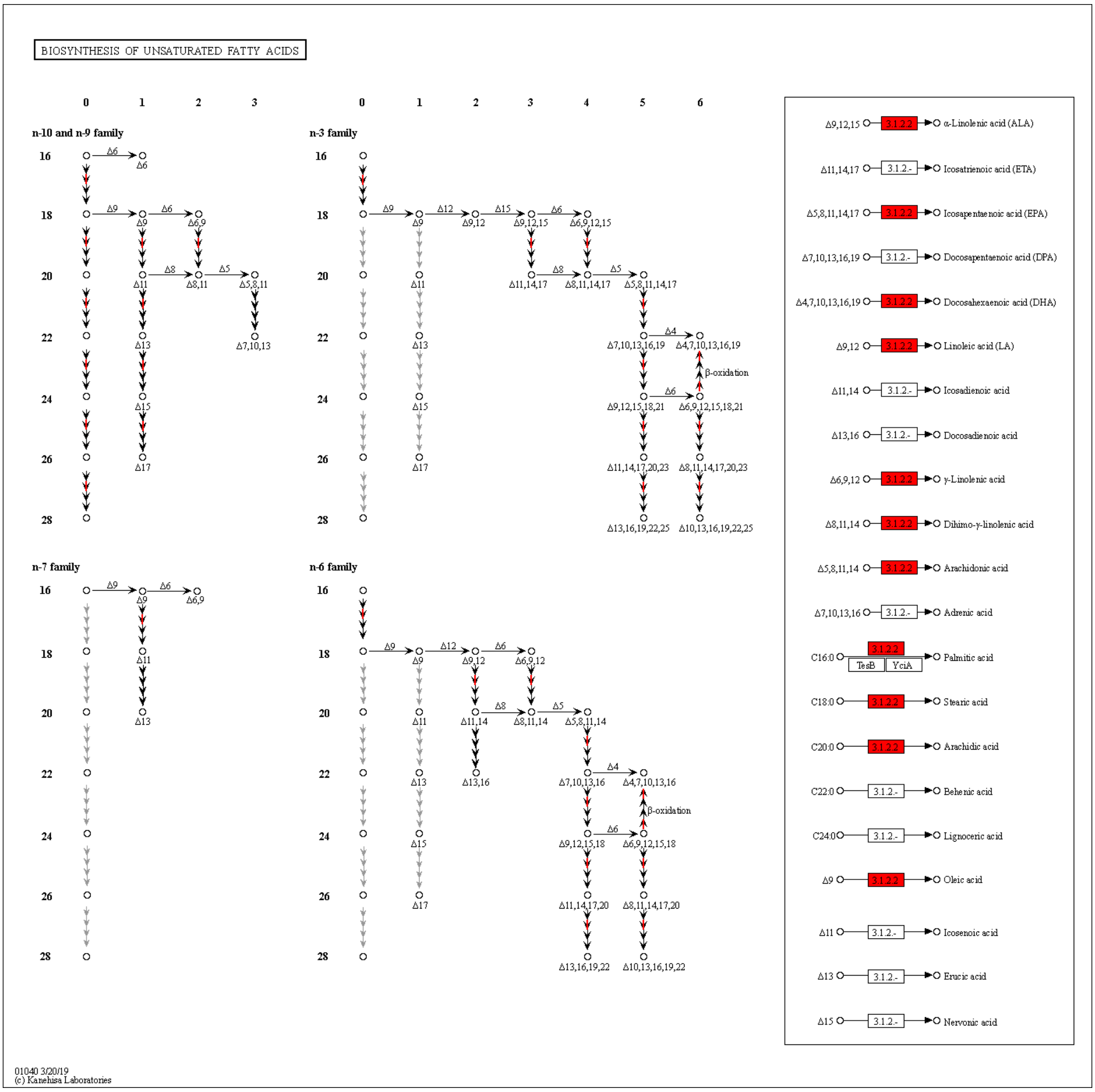
BIOSYNTHESIS OF UNSATURATED FATTY ACIDS
n-10 and n-9 family
n-3 family
n-7 family
n-6 family
β-oxidation
Δ9,12,15 3.1.2.2 α-Linolenic acid (ALA)
Δ11,14,17 3.1.2.- Icosatrienoic acid (ETA)
Δ5,8,11,14,17 3.1.2.2 Icosapentaenoic acid (EPA)
Δ7,10,13,16,19 3.1.2.- Docosapentaenoic acid (DPA)
Δ4,7,10,13,16,19 3.1.2.2 Docosahexaenoic acid (DHA)
Δ9,12 3.1.2.2 Linoleic acid (LA)
Δ11,14 3.1.2.- Icosadienoic acid
Δ13,16 3.1.2.- Docosadienoic acid
Δ6,9,12 3.1.2.2 γ-Linolenic acid
Δ8,11,14 3.1.2.2 Dihimo-γ-linolenic acid
Δ5,8,11,14 3.1.2.2 Arachidonic acid
Δ7,10,13,16 3.1.2.- Adrenic acid
C16:0 3.1.2.2 TesB YciA Palmitic acid
C18:0 3.1.2.2 Stearic acid
C20:0 3.1.2.2 Arachidic acid
C22:0 3.1.2.- Behenic acid
C24:0 3.1.2.- Lignoceric acid
Δ9 3.1.2.2 Oleic acid
Δ11 3.1.2.- Icosenoic acid
Δ13 3.1.2.- Erucic acid
Δ15 3.1.2.- Nervonic acid
01040 3/20/19
(c) Kanehisa Laboratories

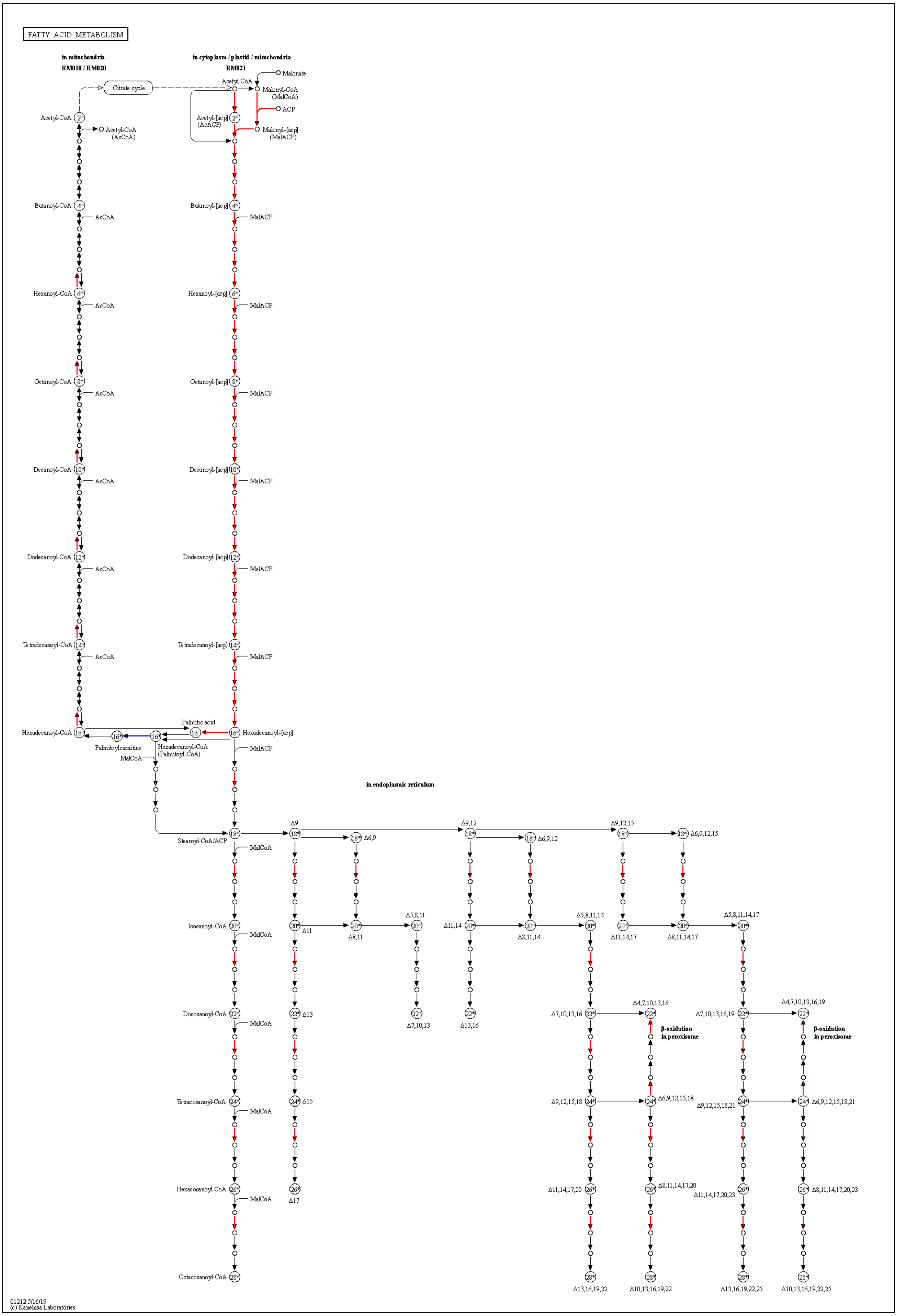
FATTY ACID METABOLISM
in mitochondria
RM018 / RM020
in cytoplasm / plastid / mitochondria
RM021
Citrate cycle
Acetyl-CoA
Malonate
Malonyl-CoA (MalCoA)
ACP
Malonyl-[acp] (MalACP)
Acetyl-[acp] (AcACP)
Acetyl-CoA (AcCoA)
Butanoyl-CoA
Butanoyl-[acp]
Hexanoyl-CoA
Hexanoyl-[acp]
Octanoyl-CoA
Octanoyl-[acp]
Decanoyl-CoA
Decanoyl-[acp]
Dodecanoyl-CoA
Dodecanoyl-[acp]
Tetradecanoyl-CoA
Tetradecanoyl-[acp]
Hexadecanoyl-CoA
Palmitic acid
Hexadecanoyl-[acp]
Palmitoylcarnitine
Hexadecanoyl-CoA (Palmitoyl-CoA)
AcCoA
MalACP
MalCoA
in endoplasmic reticulum
Stearoyl-CoA/ACP
Icosanoyl-CoA
Docosanoyl-CoA
Tetracosanoyl-CoA
Hexacosanoyl-CoA
Octacosanoyl-CoA
Δ9
Δ6,9
Δ9,12
Δ6,9,12
Δ9,12,15
Δ6,9,12,15
Δ11
Δ8,11
Δ5,8,11
Δ11,14
Δ8,11,14
Δ5,8,11,14
Δ11,14,17
Δ8,11,14,17
Δ5,8,11,14,17
Δ13
Δ7,10,13
Δ13,16
Δ7,10,13,16
Δ4,7,10,13,16
Δ7,10,13,16,19
Δ4,7,10,13,16,19
β-oxidation in peroxisome
Δ15
Δ9,12,15,18
Δ6,9,12,15,18
Δ9,12,15,18,21
Δ6,9,12,15,18,21
Δ17
Δ11,14,17,20
Δ8,11,14,17,20
Δ11,14,17,20,23
Δ8,11,14,17,20,23
Δ13,16,19,22
Δ10,13,16,19,22
Δ13,16,19,22,25
Δ10,13,16,19,22,25
01212 5/16/19
(c) Kanehisa Laboratories

Supplementary Figure S9. Enriched KEGG pathways of trans-chalcone-exposed worms compared to controls. Red: indicates pathways with predominantly upregulated genes, green: indicates predominantly downregulated genes, blue: indicates pathways containing both upregulated and underregulated genes.
